# Supplementary material for: Programmable meroterpene synthesis
Source: Nat Commun. 2020 Jan 24;11:508. doi: 10.1038/s41467-020-14354-5 (PMC6981159; doi:10.1038/s41467-020-14354-5)
Supplement: Supplementary file 2 — Supplementary Information [file 41467_2020_14354_MOESM2_ESM.pdf]

Supplementary Information

Programmable Meroterpene Synthesis

Shen et al.

Supplementary Note 1. Procedure to prepare diketene from acetyl chloride.

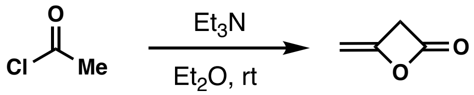

A flame-dried 500mL three-neck flask with a fritted side neck (see Supplementary Figure 1, image **A**, below) was charged with acetyl chloride (3 mL, 42 mmol, 1.2 equiv) and dry Et_2O (100 mL) under nitrogen. Freshly distilled triethylamine (5 mL, 36 mmol, 1 equiv.) was added dropwise and the resulting solution stirred vigorously at room temperature for 15 hr. Under positive nitrogen pressure, the white salts were filtered off and the solution collected in a dry three-neck flask (see Supplementary Figure 1, image **B**, below). The salts were washed with additional Et_2O (3 x 50mL) and the combined ether solution concentrated *in vacuo* using a rotary evaporator with an ice-water cooling bath. Upon removal of diethyl ether, the flask was back-filled with nitrogen and a Hickman distillation head with a side port and cooling jacket was attached (see Supplementary Figure 1, image **C**, below). The cooling jacket was cooled to –78 °C (dry ice and acetone) and the diketene was distilled at room temperature using high vacuum wherein a frozen white solid formed in the neck of the Hickman head. The high vacuum was replaced by a nitrogen atmosphere and the dry ice/acetone cooling jacket warmed to room temperature resulting in diketene as a colorless liquid (0.34 mL, 4.4 mmol 24% yield). Spectroscopic data agreed with the literature and a commercial sample from Sigma Aldrich.

a

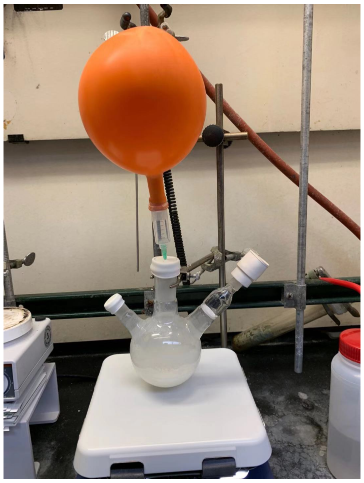

(reaction set-up)

b

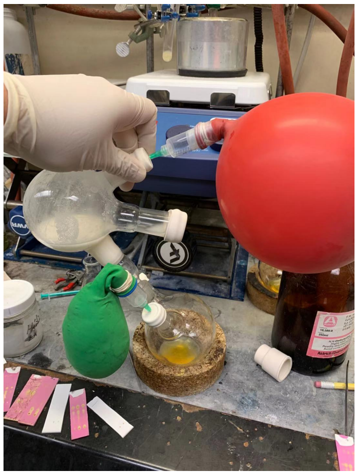

(filtration under N_2)

c

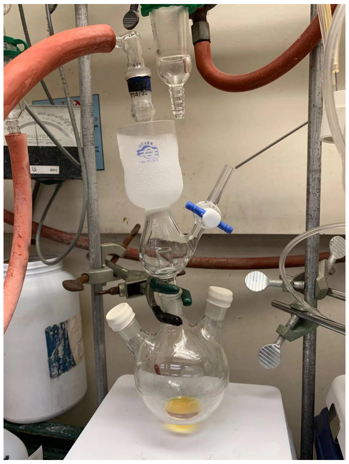

(Hickman distillation)

Supplementary Figure 1. Diketene synthesis reaction steps. **a** set-up of the reaction **b** filtration of salts under nitrogen atmosphere **c** purification of the product by Hickman distillation.

Supplementary Note 2. Procedure for the diketene annulation of Ketone **16**: formation of **17-19**.

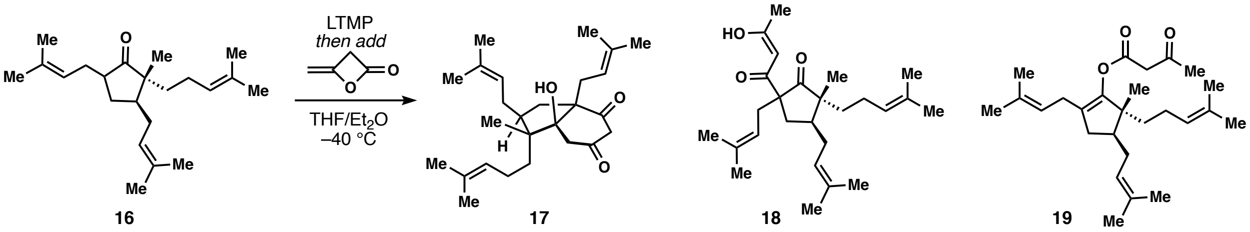

A 20 mL flame-dried reaction tube was charged with ketone **16** (100 mg, 0.32 mmol, 1.0 equiv). The reaction vessel was evacuated and backfilled with nitrogen (three times in total) followed by the addition of degassed THF (5 mL) and Et_2O (5 mL). The reaction vessel was cooled to –78 °C and freshly prepared lithium 2,2,6,6-tetramethylpiperidide (0.45 M in THF, 0.80 mL, 0.36 mmol, 1.2 equiv) was added dropwise resulting in a light-yellow colored solution. The reaction mixture was stirred for 30 minutes at –78 °C and then 60 minutes at 0 °C. After this period, the reaction mixture was cooled to –40 °C and freshly distilled diketene (30 μL, 0.38 mmol, 1.2 equiv) was rapidly in one portion resulting in a bright yellow solution. The reaction vessel was maintained at this temperature for 90 minutes and then quenched with saturated *aq*. NH_4Cl (20 mL) at this temperature. The reaction mixture was extracted with EtOAc (3 x 25 mL) and the combined organic layers were washed with brine, dried over $MgSO_4$, and concentrated *in vacuo*. The crude residue was purified by column chromatography (5% EtOAc in hexanes → 15% EtOAc in hexanes) to afford the annulated product **17** (44 mg, 35% yield) as a red/orange colored oil and recovered starting material (22 mg, 22% yield). The remaining mixed fractions were concentrated and re-chromatographed (2% Et_2O in hexanes → 10% Et_2O in hexanes) to afford **18** (8 mg, 6%) and **19** (17 mg, 14%). Compound **18** (enol tautomer): colorless oil; ^{1}H NMR (500 MHz, $CDCl_3$) δ 15.17 (s, 1H), 5.84 (s, 1H), 5.27 – 5.17 (m, 1H), 5.03 – 4.96 (m, 1H), 4.94 – 4.85 (m, 1H), 2.75 (dd, *J* = 12.8, 5.9 Hz, 1H), 2.55 (dd, *J* = 14.5, 8.0 Hz, 1H), 2.34 (dd, *J* = 14.6,7.0 Hz, 1H), 2.16 – 2.07 (m, 1H), 2.02 (s, 3H), 2.00 – 1.96 (m, 2H), 1.96 – 1.79 (m, 3H), 1.77 – 1.71 (s, 3H), 1.69 – 1.65 (s, 3H), 1.65 – 1.62 (m, 6H), 1.59 – 1.57 (m, 3H), 1.53 – 1.49 (m, 3H), 1.37 – 1.24 (m, 3H), 0.85 (s, 3H); ^{13}C NMR (150 MHz, $CDCl_3$) δ 220.9,194.5, 185.5, 135.3, 132.3, 131.6, 124.1, 122.4, 118.6, 97.8, 65.5, 52.5, 40.1, 36.8, 36.0, 32.4, 28.7, 25.8, 25.8, 25.5, 23.2, 18.7, 17.9, 17.8, 17.4. Compound **19** (mixture of enol and keto tautomers): colorless oil; ^{1}H NMR (400 MHz, $CDCl_3$) δ 12.00 (enol tautomer, s, 1H), 5.24 – 4.96 (m, 3H), 3.55 (keto tautomer, s, 2H), 2.69 – 2.52 (m, 2H), 2.30 (s, 10H), 2.15 – 1.81 (m, 20H), 1.69 (dd, *J* = 3.2, 1.5 Hz, 18H), 1.68 – 1.66 (m, 15H), 1.61 (d, *J* = 1.4 Hz, 10H), 1.59 (d, *J* = 1.3 Hz, 6H), 1.59 – 1.56 (m, 15H), 1.40 – 1.25 (m, 6H), 0.97 (td, *J* = 7.5, 5.8 Hz, 3H), 0.90 (d, *J* = 1.8 Hz, 9H); ^{13}C NMR (100 MHz, $CDCl_3$) δ 200.0, 176.5, 170.7, 164.7, 147.1, 147.0, 133.0, 132.8, 132.0, 131.9, 131.2, 131.1, 125.4, 125.0, 124.9, 124.9, 123.3, 123.2, 120.4, 120.3, 89.2, 49.9, 48.7, 48.6, 42.8, 42.4, 38.6, 38.5, 35.7, 35.7, 30.2, 29.2, 26.5, 26.4, 25.8, 25.7, 23.3, 21.3, 19.0, 17.9, 17.7, 17.7, 17.6, 17.5. Compound **17**: white solid (m.p. = 99-101 °C); ^{1}H NMR (600 MHz, $CDCl_3$) δ 5.11 – 5.00 (m, 2H), 4.95 – 4.89 (m, 1H), 3.66 (d, *J* = 18.0 Hz, 1H), 3.11 (d, *J* = 18.0 Hz, 1H), 2.79 – 2.68 (m, 2H), 2.49 (dd, *J* = 15.2, 7.0 Hz, 1H), 2.46 – 2.36 (m, 2H), 2.17 – 2.06 (m, 2H), 2.06 – 1.97 (m, 1H), 1.94 (s, 1H), 1.80 – 1.72 (m, 1H), 1.68 (s, 9H), 1.62 (s, 3H), 1.59 (s, 6H), 1.41 – 1.30 (m, 2H), 1.26 (t, *J* = 12.9 Hz, 1H), 1.17

(td, J = 13.2, 4.6 Hz, 1H), 1.02 (s, 3H); ^{13}C NMR (150 MHz, $CDCl_3$) δ 206.4, 203.8, 135.2, 132.5, 132.3, 124.5, 122.8, 118.4, 84.3, 61.2, 52.0, 50.5, 50.4, 45.0, 37.5, 35.2, 34.7, 29.2, 26.2, 26.0, 25.8, 22.9, 18.3, 18.0, 18.0, 14.9; IR (thin film) vmax 3520, 3375, 2968, 2923, 2878, 1733 cm^{-1}; HRMS (ESI$^-$) *m/z* calcd for $C_{26}H_{39}O_3$ [M-H]$^-$: 399.2905, found 399.2904.

Supplementary Note 3. General Procedure for the annulation of lithium enolates with diketene employing enolates generated by ketone deprotonation with Lithium 2,2,6,6-tetramethylpiperidide (LTMP).

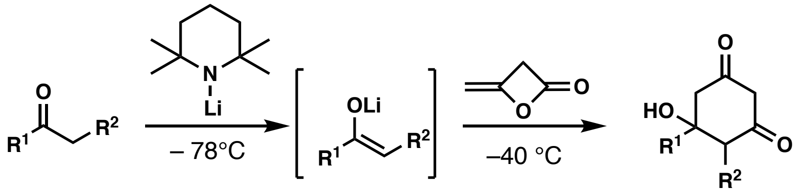

A 20 mL flame-dried reaction tube was charged with 2,2,6,6-tetramethylpiperidine (0.19 mL, 1.1 mmol, 1.1 equiv). The reaction vessel was evacuated and backfilled with nitrogen three times followed by the addition of Et_2O (2.5 mL) or THF (2.5 mL). After cooling the reaction vessel to –78°C, *n*-BuLi (2.5 M in hexanes, 0.42 mL, 1.05 mmol was added dropwise resulting in a light-yellow solution. The reaction mixture was stirred for 30 minutes at -78°C and then 15 minutes at 0 °C. After re-cooling the reaction vessel to –78 °C, the ketone (1.0 mmol, 1.1 equiv) was added dropwise as a solution in Et_2O (2.5 mL). The reaction mixture was stirred for 30 minutes at –78°C and then 30 minutes at 0°C. The reaction mixture was cooled to –40°C and freshly distilled diketene (84 µL, 1.1 mmol, 1.1 equiv) was added rapidly in one portion resulting in the formation of white precipitate. The reaction vessel was maintained at this temperature for 60 minutes then quenched with 1 M HCl (20 mL). The reaction mixture was extracted with EtOAc (3 x 25 mL) and the combined organic layers were washed with brine, dried over $MgSO_4$, and concentrated *in vacuo*. The crude residue was purified by column chromatography to afford the annulated product.

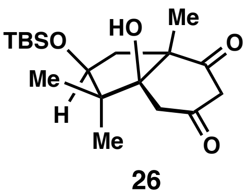

26

Diketone **26**. Following the general procedure (0.1 mmol scale) and using Et_2O/THF (*v*:*v* = 1:1) as solvent, the title compound was obtained after column chromatography (20% EtOAc in hexanes) as a white solid (14.3 mg, 41% yield): ^{1}H NMR (700 MHz, $CDCl_3$) δ 4.33 (d, J = 1.0 Hz, 1H), 3.74 (d, J = 1.1 Hz, 1H), 3.72 (d, J = 5.8 Hz, 1H), 3.26 (dd, J = 17.0, 2.4 Hz, 1H), 3.13 (dd, J = 15.4, 5.9 Hz, 1H), 2.84 (dd, J = 16.1, 2.4 Hz, 1H), 2.67 (d, J = 16.1 Hz, 1H), 1.57 (d, J = 15.4 Hz, 1H), 1.48 (s, 3H), 1.06 (s, 3H), 0.91 (s, 9H), 0.49 (s, 3H), 0.10 (s, 4H), 0.08 (s, 4H); ^{13}C NMR (151 MHz, $CDCl_3$) δ 208.7, 202.0, 83.8, 81.8, 59.8, 54.4, 51.0, 46.6, 42.1, 25.8, 24.1, 22.1, 18.0, 17.9, -4.7, -5.0; IR (thin film) vmax 3464, 2956, 2929, 2884, 1727, 1601 cm^{-1}; HRMS (ESI-) *m/z* calcd. for $C_{18}H_{31}O_4Si$ [M-H]$^-$: 339.1997, found: 339.1997.

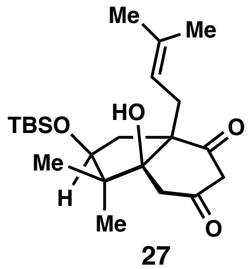
27

Diketone **27**. Following the general procedure (0.1 mmol scale) and using Et_2O/THF (*v:v* = 1:1) as solvent, the title compound was obtained after column chromatography (20% EtOAc in hexanes) as a white solid (19.1 mg, 49% yield): ^{1}H NMR (600 MHz, CD_2Cl_2) δ 5.07 (td, *J* = 6.9, 3.2 Hz, 1H), 4.29 (s, 1H), 3.75 (d, *J* = 5.7 Hz, 1H), 3.59 (d, *J* = 16.9 Hz, 1H), 3.19 (dd, *J* = 16.9, 2.2 Hz, 1H), 2.96 (dd, *J* = 15.5, 5.8 Hz, 1H), 2.78 (dd, *J* = 16.1, 2.2 Hz, 1H), 2.74 – 2.65 (m, 2H), 2.59 (dd, *J* = 15.0, 6.9 Hz, 1H), 1.70 (d, *J* = 1.5 Hz, 3H), 1.64-1.60 (m, 4H), 1.04 (s, 3H), 0.91 (s, 9H), 0.47 (s, 3H), 0.11 (s, 3H), 0.09 (s, 3H); ^{13}C NMR (151 MHz, CD_2Cl_2) δ 207.9, 202.4, 135.7, 119.5, 84.2, 82.3, 64.0, 56.4, 51.40, 47.7, 40.5, 36.3, 26.1, 25.9, 22.2, 18.1, 18.1, 17.9, -4.7, -5.1; IR (thin film) vmax 3468, 2955, 2929, 2929, 2857, 1736, 1606 cm^{-1}; HRMS (ESI-) *m/z* calcd. for $C_{22}H_{37}O_4Si$ [M-H]- : 393.2467, found: 393.2467.

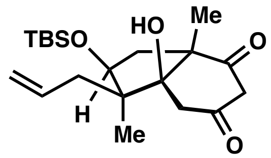
28

Diketone **28**. Following the general procedure (0.1 mmol scale) and using Et_2O/THF (*v:v* = 1:1) as solvent, the title compound was obtained after column chromatography (50% EtOAc in hexanes) as a light yellow solid (11 mg, 31% yield): ^{1}H NMR (600 MHz, $CDCl_3$) δ 5.75 (dt, *J* = 9.8, 5.9 Hz, 1H), 5.21 – 5.07 (m, 2H), 4.40 (s, 1H), 3.87 (d, *J* = 5.5 Hz, 1H), 3.79 – 3.68 (m, 1H), 3.28 (dd, *J* = 17.3, 2.2 Hz, 1H), 3.07 (dd, *J* = 15.6, 5.6 Hz, 1H), 2.92 (dd, *J* = 15.9, 2.2 Hz, 1H), 2.69 (d, *J* = 15.9 Hz, 1H), 2.64 (dd, *J* = 14.1, 5.4 Hz, 1H), 2.11 (dd, *J* = 14.1, 9.2 Hz, 1H), 1.64 (d, *J* = 15.5 Hz, 1H), 0.94 (s, 9H), 0.47 (s, 3H), 0.17 (s, 3H), 0.13 (s, 3H); ^{13}C NMR (151 MHz, $CDCl_3$) δ 208.7, 202.0, 134.2, 118.6, 84.3, 80.3, 60.0, 54.2, 53.3, 47.1, 41.7, 35.0, 25.9, 24.0, 18.8, 17.9, -4.0, -5.0; IR (thin film) vmax 3458, 2952, 2929, 2857, 1592, 1462, 1408, 1390 cm^{-1}; HRMS (ESI-) *m/z* calcd. for $C_{20}H_{33}O_4Si$ [M-H]-: 365.2154, found: 365.2155.

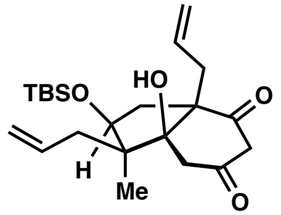
29

Diketone **29**. Following the general procedure (0.1 mmol scale) and using Et_2O/THF (*v:v* = 1:1) as solvent, the title compound was obtained after column chromatography (50% EtOAc in hexanes) as a light yellow solid (11 mg, 31% yield): ^{1}H NMR (600 MHz, $CDCl_3$) δ 5.95 – 5.60 (m, 2H), 5.31 – 4.98 (m, 4H), 4.48 (s, 1H), 3.90 (d, *J* = 5.5 Hz, 1H), 3.59 (d, *J* = 17.3 Hz, 1H), 3.40 – 3.23 (m, 1H), 2.93 (ddd, *J* = 15.9, 5.8, 1.6 Hz, 2H), 2.76 – 2.67 (m, 3H), 2.65 – 2.59 (m, 1H), 2.10 (dd, *J* = 14.1, 9.1 Hz, 1H), 1.75 (d, *J* = 16.1 Hz, 1H), 0.95 (s, 9H), 0.49 (s, 3H), 0.17 (s, 3H), 0.14 (s, 3H); ^{13}C NMR (151 MHz, $CDCl_3$) δ 207.0, 201.8, 134.0, 133.3, 118.8, 118.5, 84.5, 80.3, 63.9, 55.7, 53.3, 47.7, 41.7, 39.4, 34.8, 25.8, 18.7, 17.8, -4.1, -5.2; IR (thin film) vmax 3459, 3075, 1952, 1929, 2857, 1736, 1599, 1453, 1435 cm^{-1}; HRMS (ESI-) *m/z* calcd. for $C_{22}H_{35}O_4Si$ [M-H]-: 391.2310, found: 391.2309.

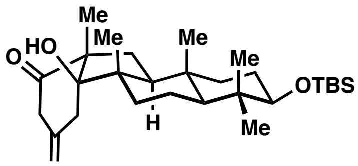
30

Diketone **30**: Following the general procedure (0.13 mmol scale) and using Et_2O/THF (*v:v* = 1:1) as solvent, the title compound was isolated by silica gel column chromatography (2% EtOAc in hexanes → 20% EtOAc in hexanes) as a white solid (18.0 mg, 30% yield, 47% BRSM):

m.p. = 205-207 C; ^{1}H NMR (600 MHz, $CDCl_3$) δ 3.68 (d, *J* = 18.7 Hz, 1H), 3.14 (dd, *J* = 11.5, 4.6 Hz, 1H), 3.11 (d, *J* = 18.5 Hz, 1H), 2.63 (d, *J* = 15.0 Hz, 1H), 2.58 (dd, *J* = 12.9, 6.3 Hz, 1H), 2.43 (d, *J* = 14.9 Hz, 1H), 1.89 (s, 1H), 1.68-1.56 (m, 2H), 1.54-1.42 (m, 4H), 1.40-1.35 (m, 1H), 1.34 (s, 3H), 1.22-1.14 (m, 1H), 1.08 (s, 3H), 1.02-0.93 (m, 2H), 0.92 (s, 3H), 0.88 (s, 12H), 0.76 (s, 3H), 0.66 (dd, *J* = 12.2, 2.3 Hz, 1H), 0.02 (s, 3H), 0.01 (s, 3H); ^{13}C NMR (101 MHz, $CDCl_3$) δ 207.9, 204.2, 82.1, 79.5, 57.8, 56.4, 54.5, 51.1, 50.8, 48.8, 39.5, 38.6, 36.7, 34.6, 30.2, 28.6, 27.6, 26.1, 24.9, 19.0, 18.3, 16.3, 16.3, 15.9, -3.6, -4.8; IR (thin film) vmax 3406, 2927, 2854, 1726, 1701, 1468, 1387, 1362, 1305, 1254, 1104, 1088, 1062, 1045, 1006, 984, 912, 880, 832, 772 cm^{-1}; HRMS (ESI) *m/z* calcd. for $[C_{28}H_{47}O_4Si]^-$(M-H)$^-$: 475.3249, found 475.3242.

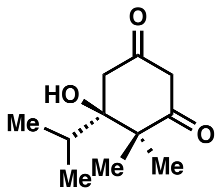

31

Diketone **31**: Following the general procedure (0.42 mmol scale) and using Et_2O/THF (*v:v* = 1:1) as solvent, the title compound was isolated by silica gel column chromatography (50%→60% EtOAc in hexanes) as a white solid (49 mg, 59% yield): m.p. = 108-113 °C; ^{1}H NMR (400 MHz, $CDCl_3$) δ 3.43 (d, *J* = 20.0 Hz, 1H), 3.38 (d, *J* = 20.0 Hz, 1H), 2.87 (d, *J* = 17.0 Hz, 1H), 2.64 (d, *J* = 17.0 Hz, 1H), 2.10 (sept, *J* = 6.9 Hz, 1H), 2.02 (bs, 1H), 1.30 (s, 3H), 1.26 (s, 3H), 1.00 (d, *J* = 6.9 Hz, 3H), 0.95 (d, *J* = 6.9 Hz, 3H); ^{13}C NMR (100 MHz, $CDCl_3$) δ 207.1, 204.3, 77.7, 53.6, 53.1, 43.4, 34.8, 21.9, 19.1, 18.3, 18.0; IR (thin film) vmax 3595, 3473, 2988, 2970, 2571, 1611 cm^{-1}; HRMS (ESI): *m/z* calcd. for $[C_{11}H_{17}O_3]^-$ (M-H)$^-$: 197.1183, found 197.1175.

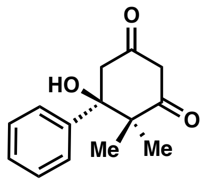

32

Diketone **32**: Following the general procedure (0.40 mmol scale) using Et_2O as solvent, the title compound was isolated by silica gel column chromatography (40%→50% EtOAc in hexanes) as a white solid (41 mg, 44% yield): m.p. = 135-137 °C; ^{1}H NMR (500 MHz, $CDCl_3$) δ 7.43 – 7.38 (m, 4H), 7.38 – 7.33 (m, 1H), 3.74 (d, *J* = 16.1 Hz, 1H), 3.65 (d, *J* = 18.4 Hz, 1H), 3.46 (dd, *J* = 18.4, 2.2 Hz, 1H), 2.72 (dd, *J* = 16.2, 2.2 Hz, 1H), 2.23 (bs, 1H), 1.22 (s, 3H), 1.02 (s, 3H); ^{13}C NMR (100 MHz, $CDCl_3$) δ 206.7, 202.8, 141.3, 128.4, 128.2, 126.3, 78.3, 54.0, 52.7, 50.8, 22.7, 17.9; IR (thin film) vmax 3379, 2985, 2925, 2892, 1734, 1700 cm^{-1}; HRMS (ESI): *m/z* calcd. for $[C_{14}H_{15}O_3]^-$ (M-H)$^-$: 231.1027, found 231.1037.

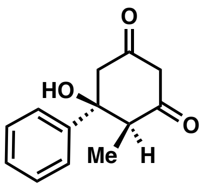

33

Diketone **33:** Following the general procedure (1.0 mmol scale) using Et_2O as solvent, the title compound was isolated by silica gel column chromatography (20%→50% EtOAc in hexanes) as a white crystalline solid (138 mg, 63% yield): m.p. = 171-173 °C; ^{1}H NMR (400 MHz, $CDCl_3$) δ 7.46 – 7.37 (m, 4H), 7.36 – 7.30 (m, 1H), 3.60 (d, *J* = 20.0 Hz, 1H), 3.55 (d, *J* = 20.0 Hz, 1H), 3.26 (d, *J* = 15.8 Hz, 1H), 3.18 (q, *J* = 6.8 Hz, 1H), 2.88 (dd, *J* = 15.7, 1.6 Hz, 1H), 2.14 (bs, 1H), 0.94 (d, *J* = 6.8 Hz, 3H); ^{13}C NMR (150 MHz, $CDCl_3$) δ 203.0, 201.6, 143.3, 129.1, 127.9, 124.5, 76.2, 57.3, 55.8, 53.4, 8.5; IR (thin film) vmax 3379, 3029, 2996, 2899, 1726, 1700 cm^{-1}; HRMS (EI): *m/z* calcd. for $[C_{13}H_{14}O_3]$: 218.0941, found 218.0943

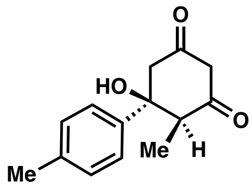

34

Diketone **34:** Following the general procedure (0.4 mmol scale) using Et_2O as solvent, the title compound was isolated by silica gel column chromatography (50% EtOAc in hexanes) as a light yellow solid (60 mg, 65% yield): ^{1}H NMR (600 MHz, $CDCl_3$) δ 7.28 – 7.25 (d, *J* = 8.0 Hz, 2H), 7.22 (d, *J* = 8.0 Hz, 2H), 3.57 (d, *J* = 17.5 Hz, 1H), 3.53 (dd, *J* = 17.5, 2.0 Hz, 1H), 3.23 (d, *J* = 15.7 Hz, 1H), 3.14 (q, *J* = 6.8 Hz, 1H), 2.85 (dd, *J* = 15.7, 2.0 Hz, 1H), 2.37 (s, 3H), 0.93 (d, *J* = 6.8 Hz, 3H); ^{13}C NMR (151 MHz, $CDCl_3$) δ 203.2, 202.0, 140.3, 137.5, 129.6, 124.3, 76.0, 57.2, 55.7, 53.4, 21.0, 8.4; IR (thin film) vmax 3428, 2986, 2922, 2876, 1731, 1703, 1605, 1513, 1454, 1404 cm^{-1}; HRMS (ESI-) *m/z* calcd. for $C_{14}H_{15}O_3$ $[M-H]^-$: 231.1027, found: 231.1027.

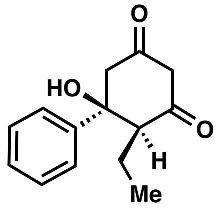

35

Diketone **35:** Following the general procedure (0.4 mmol scale) using Et_2O as solvent, the title compound was isolated by silica gel column chromatography (50% EtOAc in hexanes) as a light yellow solid (48 mg, 52% yield): ^{1}H NMR (500 MHz, $CDCl_3$) δ 7.47 – 7.38 (m, 4H), 7.32 (td, *J* = 6.4, 2.5 Hz, 1H), 3.62 (d, *J* = 16.7 Hz, 1H), 3.50 (dd, *J* = 16.8, 2.5 Hz, 1H), 3.21 (d, *J* = 15.6 Hz, 1H), 2.96 (dd, *J* = 9.1, 2.5 Hz, 1H), 2.81 (dd, *J* = 15.7, 2.4 Hz, 1H), 1.78 (ddq, *J* = 14.3, 9.1, 7.2 Hz, 1H), 1.11 (dtd, *J* = 15.0, 7.5, 2.4 Hz, 1H), 0.82 (t, *J* = 7.4 Hz, 3H); ^{13}C NMR (151 MHz, $CDCl_3$) δ 202.3, 202.0, 143.7, 129.0, 127.7, 124.5, 76.7, 60.6, 58.4, 56.1, 17.0, 13.5; IR (thin film) vmax 3399, 2059, 3027, 2963, 2932, 2874, 1731, 1703, 1599 1494, 1446 cm^{-1}; HRMS (ESI-) *m/z* calcd. for $C_{14}H_{15}O_3$ $[M-H]^-$: 231.1027, found: 231.1027.

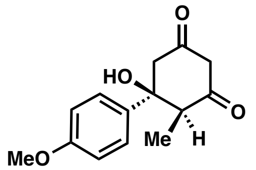

36

Diketone **36**: Following the general procedure (0.4 mmol scale) using Et_2O as solvent, the title compound was isolated by silica gel column chromatography (50% EtOAc in hexanes) as a light yellow solid (56 mg, 56% yield): ^{1}H NMR (600 MHz, $CDCl_3$) δ 7.29 (d, *J* = 8.7 Hz, 2H), 6.94 (d, *J* = 8.8 Hz, 2H), 3.83 (s, 3H), 3.58 – 3.52 (m, 2H), 3.23 (d, *J* = 15.7 Hz, 1H), 3.13 (q, *J* = 6.8 Hz, 1H), 2.86 (dd, *J* = 15.7, 1.6 Hz, 1H), 2.10 (s, 1H), 0.95 (d, *J* = 6.8 Hz, 3H); ^{13}C NMR (151 MHz, $CDCl_3$) δ 203.2, 201.9, 159.2, 135.4, 125.8, 114.4, 76.0, 57.3, 56.0, 55.5, 53.6, 8.5; IR (thin film) vmax 3446, 3398, 2988, 2940, 1733, 1706, 1653, 1609, 1559, 1533, 1512 cm^{-1}; HRMS (ESI-) *m/z* calcd. for $C_{14}H_{15}O_4$ $[M-H]^-$: 247.0976, found: 247.0977.

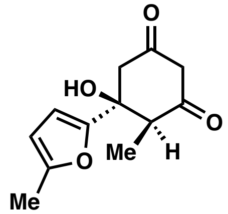

37

Diketone **37**: Following the general procedure (1.0 mmol scale) using Et_2O as solvent, the title compound was isolated by silica gel column chromatography (50% EtOAc in hexanes) as a brown oil (97 mg, 44% yield): ^{1}H NMR (500 MHz, $CDCl_3$) δ 6.18 (d, *J* = 3.0 Hz, 1H), 5.93 (d, *J* = 3.0 Hz, 1H), 3.50 (s, 2H), 3.28 (d, *J* = 16.0 Hz, 1H), 3.14 (q, *J* = 6.8 Hz, 1H), 2.90 (d, *J* = 16.0 Hz, 1H), 2.70 (bs, 1H), 2.28 (s, 3H), 1.04 (d, *J* = 6.8 Hz, 3H); ^{13}C NMR (100 MHz, $CDCl_3$) δ 203.1, 201.9, 153.5, 152.5, 107.2, 106.6, 73.3, 57.1, 53.4, 52.3, 13.7, 8.7; IR (thin film) vmax 3357, 2985, 2925, 1707, 1603, 1451 cm^{-1}; HRMS (ESI): *m/z* calcd. for $[C_{12}H_{13}O_4]^-$ $(M-H)^-$: 221.0819, found 221.0830.

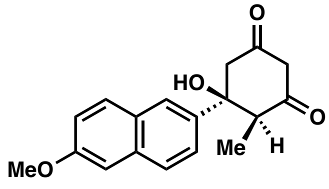

38

Diketone **38:** Following the general procedure (3.6 mmol scale) using Et_2O as solvent, the title compound was isolated by silica gel column chromatography (20%→50% EtOAc in hexanes) as a light brown solid (697 mg, 65% yield): m.p. = 180-183 °C; ^1H NMR (400 MHz, $CDCl_3$) δ 7.87 – 7.78 (m, 2H), 7.75 (d, J = 8.9 Hz, 1H), 7.40 (dd, J = 8.7, 2.1 Hz, 1H), 7.20 (dd, J = 8.9, 2.5 Hz, 1H), 7.15 (d, J = 2.5 Hz, 1H), 3.94 (s, 3H), 3.61 (d, J = 3.3 Hz, 2H), 3.35 (d, J = 15.8 Hz, 1H), 3.28 (q, J = 6.8 Hz, 1H), 2.93 (d, J = 15.8 Hz, 1H), 2.16 (bs, 1H), 0.96 (d, J = 6.8 Hz, 3H); ^{13}C NMR (150 MHz, $CDCl_3$) δ 203.0, 201.6, 158.4, 138.4, 133.9, 129.8, 128.8, 127.9, 123.4, 122.8, 119.8, 105.8, 76.4, 57.4, 55.8, 55.6, 53.3, 8.6; IR (thin film) vmax 3357, 3003, 2907, 1704, 1626, 1611 cm^{-1}; HRMS (ESI): *m/z* calcd for $[C_{18}H_{17}O_4]^-$ $(M-H)^-$: 297.1132, found 297.1133.

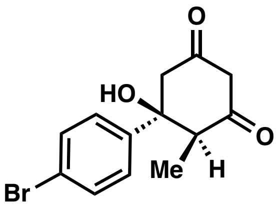

39

Diketone **39:** Following the general procedure (1.0 mmol scale) using Et_2O as solvent, the title compound was isolated by silica gel column chromatography (20% →60% EtOAc in hexanes) as an orange solid (184 mg, 62% yield): m.p. = 155-158 °C; ^1H NMR (400 MHz, $CDCl_3$) δ 7.55 (d, J = 8.7 Hz, 2H), 7.28 (d, J = 8.7 Hz, 2H), 3.58 (d, J = 20.0 Hz, 1H), 3.53 (dd, J = 20.0, 1.9 Hz, 1H), 3.22 (d, J = 15.7 Hz, 1H), 3.13 (q, J = 6.8 Hz, 1H), 2.85 (dd, J = 15.8, 1.9 Hz, 1H), 2.38 (bs, 1H), 0.93 (d, J = 6.8 Hz, 3H); ^{13}C NMR (100 MHz, $CDCl_3$) δ 202.5, 201.4, 142.5, 132.2, 126.4, 122.0, 76.0, 57.3, 55.5, 53.3, 8.4; IR (thin film) vmax 3569, 3368, 2985, 2903, 1700, 1588 cm^{-1}; HRMS (ESI): *m/z* calcd. for $[C_{13}H_{12}BrO_3]^-$ $(M-H)^-$: 294.9975, found 294.9991.

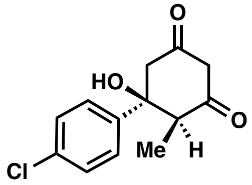

40

Diketone **40**: Following the general procedure (0.4 mmol scale) using Et_2O as solvent, the title compound was isolated by silica gel column chromatography (50% EtOAc in hexanes) as a light yellow solid (45 mg, 45% yield): ^1H NMR (600 MHz, $CDCl_3$) δ 7.40 (d, J = 8.6 Hz, 2H), 7.34 (d, J = 8.6 Hz, 2H), 3.82 – 3.48 (m, 2H), 3.22 (d, J = 15.7 Hz, 1H), 3.14 (q, J = 6.8 Hz, 1H), 2.86 (dd, J = 15.7, 1.8 Hz, 1H), 0.94 (d, J = 6.8 Hz, 3H); ^{13}C NMR (151 MHz, $CDCl_3$) δ 202.5, 201.3, 141.9, 133.9, 129.3, 126.1, 76.0, 57.3, 55.6, 53.3, 8.4; IR (thin film) vmax 3394, 2941, 2349, 1733, 1703, 1606, 1491, 1455 cm^{-1}; HRMS (ESI-): *m/z* calcd. for $C_{13}H_{12}O_3{}^{35}Cl]^-$ (M-H)-: 251.0480, found: 251.0482.

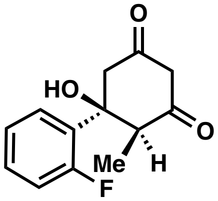

41

Diketone **41:** Following the general procedure (1.0 mmol scale) using Et_2O as solvent, the title compound was isolated by silica gel column chromatography (20%→50% EtOAc in hexanes) as a white crystalline solid (160 mg, 68% yield): m.p. = 154-158 °C; ^1H NMR (400 MHz, $CDCl_3$) δ 7.51 (td, J = 8.1, 1.8 Hz, 1H), 7.41 – 7.32 (m, 1H), 7.23 (td, J = 7.7, 1.3 Hz, 1H), 7.10 (ddd, J = 12.2, 8.2, 1.2 Hz, 1H), 3.67 – 3.44 (m, 4H), 2.81 (dd, J = 15.8, 2.3 Hz, 1H), 2.42 (bs, 1H), 0.92 (d, J = 6.9 Hz, 3H); ^{13}C NMR (100 MHz, $CDCl_3$) δ 202.6, 201.8, 158.7 (d, J = 244.4 Hz), 130.3 (d, J = 8.6 Hz), 130.0 (d, J = 12.4 Hz), 127.0 (d, J = 3.7 Hz), 125.0 (d, J = 3.6 Hz), 116.5 (d, J = 23.4 Hz), 74.9 (d, J = 4.4 Hz), 57.4, 53.3 (d, J = 3.6 Hz), 51.5 (d, J = 4.1 Hz), 8.5; IR (thin film) vmax 3391, 2925, 2854, 1730, 1700, 1488 cm^{-1}; HRMS (ESI): *m/z*

calcd. for $[C_{13}H_{12}FO_3]^-$ $(M-H)^-$: 235.0776, found 235.0792.

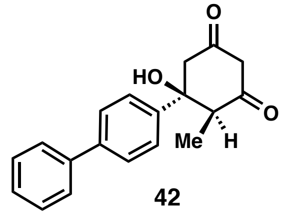

42

Diketone **42**: Following the general procedure (0.2 mmol scale) using Et_2O as solvent, the title compound was isolated by silica gel column chromatography (50% EtOAc in hexanes) as a light yellow solid (18 mg, 32% yield, 91% BRSM): ^1H NMR (600 MHz, $CDCl_3$) δ 7.65 (d, J = 8.3 Hz, 2H), 7.63 – 7.58 (m, 2H), 7.46 (dt, J = 8.0, 3.5 Hz, 4H), 7.41 – 7.33 (m, 1H), 3.59 (d, J = 1.5 Hz, 2H), 3.29 (d, J = 15.7 Hz, 1H), 3.21 (q, J = 6.8 Hz, 1H), 2.93 (dd, J = 15.7, 1.6 Hz, 1H), 2.21 (s, 1H), 1.00 (d, J = 6.8 Hz, 3H); ^{13}C NMR (151 MHz, $CDCl_3$) δ 203.0, 201.6, 142.3, 140.8, 140.3, 129.0, 127.8, 127.8, 127.2, 125.0, 76.2, 57.4, 55.8, 53.4, 8.6; IR (thin film) vmax 3742 3734 3614 3398 1742 1707 1627 1607 1491 cm^{-1}; HRMS (ESI^-) *m/z* calcd. for $C_{19}H_{17}O_3$ $[M-H]^-$: 293.1183, found: 293.1185.

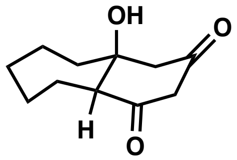

44

Diketone **44**: Following the general procedure (1.0 mmol scale) using Et_2O as solvent, the title compound (major isomer) was isolated by silica gel column chromatography (30%→40% EtOAc in hexanes) as a brown solid (61 mg, 31% yield). The remaining column fractions were concentrated and re-purified by silica gel column chromatography (0%→7.5% MeOH in CH_2Cl_2) to give the minor diastereomer as a yellow solid (42 mg, 21% yield). Major diastereomer: m.p. = 116-118 °C; ^1H NMR (400 MHz, $CDCl_3$) δ 3.41 (dd, J = 18.3, 1.9 Hz, 1H), 3.31 (d, J = 18.2 Hz, 1H), 2.89 (d, J = 16.2 Hz, 1H), 2.71 – 2.58 (m, 2H), 2.37 – 2.24 (m, 1H), 2.10 (bs, 1H), 2.08 – 1.96 (m, 2H), 1.88 – 1.76 (m, 2H), 1.72 (dd, J = 14.3, 10.1 Hz, 1H), 1.53 – 1.41 (m, 2H), 1.41 – 1.21 (m, 2H); ^{13}C NMR (100 MHz, $CDCl_3$) δ 204.3, 203.3, 74.4, 60.4, 56.0, 55.3, 42.6, 29.5, 28.1, 22.1, 20.6; IR (thin film) vmax 3391, 2925, 2862, 1726, 1700, 1451 cm^{-1}; HRMS (ESI): *m/z* calcd. for $[C_{11}H_{15}O_3]^-$ $(M-H)^-$: 195.1027, found 195.1055.

Supplementary Note 4. General Procedure for the annulation of lithium enolates with diketene using enolates generated by desilylation of trimethylsilyl enol ethers with methyl lithium.

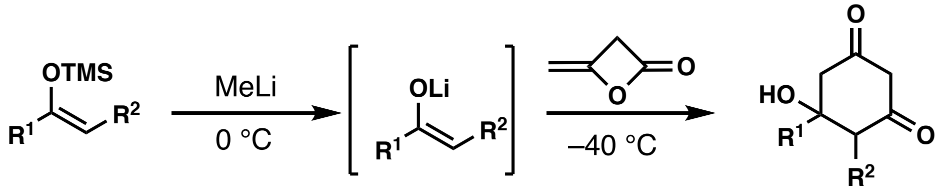

A flame-dried reaction tube was charged with the trimethylsilyl enol ether (0.89 mmol, 1.1 equiv). The reaction vessel was evacuated and backfilled with nitrogen (3 times in total) and Et_2O (4 mL) was added. After cooling the reaction to 0 °C, methyllithium (1.6 M in Et_2O, 0.80 mmol, 1.0 equiv) was added and the reaction mixture stirred for 1 hour. The reaction mixture was warmed to room temperature and monitored by TLC for consumption of the starting silyl enol ether. The reaction vessel was then cooled to -40 °C and freshly distilled diketene (0.89 mmol, 1.0 equiv) was rapidly added resulting in the formation of white precipitate. The suspension was stirred for 1 hour and then quenched with 1 M HCl (5 mL) and slowly warmed to room temperature. The reaction mixture was diluted in EtOAc (15 mL) and the layers separated. The aqueous layer was extracted with EtOAc (2 x 20 mL) and the combined organic layers were washed with brine, dried over $MgSO_4$, and concentrated *in vacuo* to afford a yellow oil. The crude residue was purified by silica gel column chromatography to afford the annulated product.

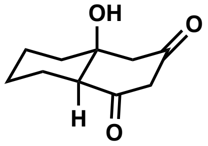

43

Diketone **43:** Following the general procedure (0.80 mmol scale), the major *trans* diastereomer was isolated by silica gel column chromatography (50%→100% EtOAc in hexanes) as a white solid (40 mg, 27% yield). The remaining column fractions were concentrated and re-purified by silica gel column chromatography (5%→7.5% MeOH in CH_2Cl_2) to afford the *cis* diastereomer as a yellow solid (27 mg, 19% yield). **43** (major diastereomer): m.p. = 145-147 °C; ^{1}H NMR (600 MHz, $CDCl_3$) δ 3.43 (dd, *J* = 18.0, 1.6 Hz, 1H), 3.40 (d, *J* = 18.0 Hz, 1H), 2.77 (d, *J* = 15.5 Hz, 1H), 2.72 (dd, *J* = 15.5, 1.5 Hz, 1H), 2.51 (dd, *J* = 12.1, 4.2 Hz, 1H), 2.03 – 1.96 (m, 1H), 1.93 – 1.87 (m, 1H), 1.80 (ddt, *J* = 13.4, 4.0, 1.7 Hz, 1H), 1.72 (bs, 1H), 1.71 – 1.66 (m, 1H), 1.63 – 1.56 (m, 2H), 1.56 – 1.49 (m, 1H), 1.28 (qt, *J* = 13.3, 3.7 Hz, 1H); ^{13}C NMR (150 MHz, $CDCl_3$) δ 203.4, 202.4, 70.8, 57.5, 55.2, 55.1, 38.9, 24.6, 21.3, 21.0; IR (thin film) vmax 3383, 2981, 2936, 2854, 1726, 1700 cm^{-1}; HRMS (ESI): calcd. for $[C_{10}H_{13}O_3]^-$ $(M-H)^-$: *m/z* 181.0870, found 181.0872.

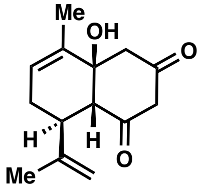

45

Diketone **45**. Following the general procedure (0.82 mmol scale), the title compound was isolated by silica gel column chromatography (50%→100% EtOAc in hexanes) as a white solid (67 mg, 35% yield): ^{1}H NMR (400 MHz, $CDCl_3$) δ 5.50 (s, 1H), 4.84 (s, 1H), 4.79 (s, 1H), 3.49 (d, *J* = 16.5Hz, 1H), 3.31 (d, *J* = 16.4, 1H), 2.99 – 2.86 (m, 2H), 2.85 – 2.77 (m, 1H), 2.29 (ddt, *J* =15.6, 10.4, 2.6 Hz, 1H), 2.22 – 2.08 (m, 1H), 1.77 (s,

6H); ^{13}C NMR (100 MHz, $CDCl_3$) δ 204.2, 201.0, 145.2, 136.5, 123.7, 114.2, 73.3, 60.8, 56.5, 48.5, 45.6, 30.3, 17.5, 16.8; IR (thin film) vmax 3072, 2956, 1919, 1771, 1605, 1439, 1405, 1377, 1321, 1288 cm^{-1}; HRMS (ESI) *m/z* calcd. for $C_{14}H_{19}O_3$ $[M+H]^+$: 235.1329, found: 235.1330.

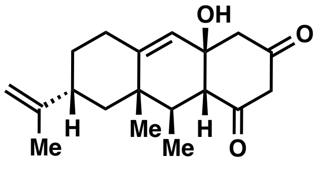

46

Diketone **46**: Following the general procedure (1.0 mmol scale), the title compound was isolated by silica gel column chromatography (100% EtOAc) as a white solid (121 mg, 40% yield): m.p. = 164-166 °C; ^{1}H NMR (600 MHz, $CDCl_3$) δ 5.35 (s, 1H), 4.73 (s, 1H), 4.69 (s, 1H), 3.43 (d, *J* = 16.2 Hz, 1H), 3.36 (d, *J* = 16.2 Hz, 1H), 2.91 (d, *J* = 15.4 Hz, 1H), 2.72 (d, *J* = 15.4 Hz, 1H), 2.61 (d, *J* = 12.2 Hz, 1H), 2.39 – 2.32 (m, 1H), 2.28 (t, *J* = 12.4 Hz, 1H), 2.17 – 2.11 (m, 1H), 2.05 – 1.99 (m, 1H), 1.97 (d, *J* = 12.7 Hz, 1H), 1.88 (d, *J* = 12.8 Hz, 1H), 1.72 (s, 3H), 1.30 – 1.18 (m, 1H), 1.08 (s, 3H), 1.02 (t, *J* = 12.7 Hz, 1H), 0.91 (d, *J* = 6.9 Hz, 3H); ^{13}C NMR (150 MHz, $CDCl_3$) δ 205.5, 200.7, 149.4, 145.7, 125.1, 109.3, 71.5, 59.8, 56.9, 50.3, 44.8, 41.8, 40.7, 40.1, 32.8, 32.3, 21.0, 18.3, 12.6; IR (thin film) vmax 3346, 2974, 2933, 2854, 1596, 1529 cm^{-1}; HRMS (ESI): calcd. for $[C_{19}H_{25}O_3]^-$ $(M-H)^-$: *m/z* 301.1809, found 301.1822.

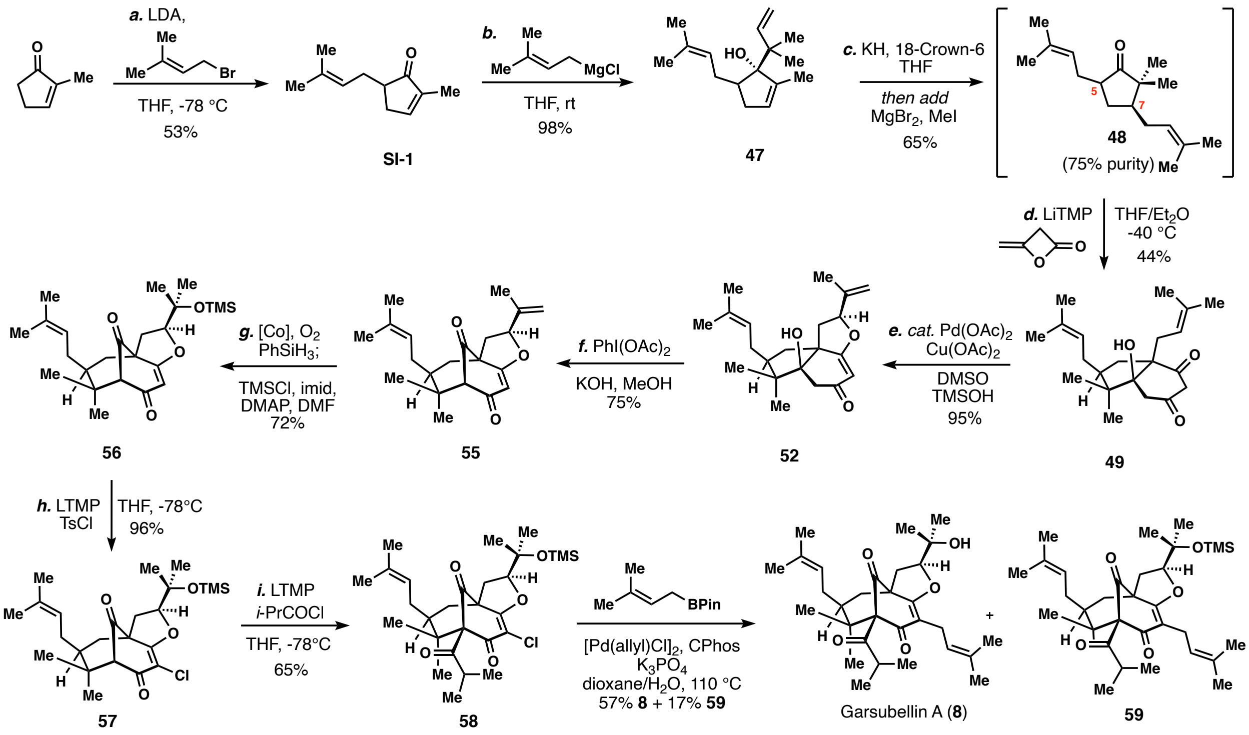

Supplementary Figure 2. Synthetic pathway to garsubellin A (**8**).

Supplementary Note 5. General Procedures for the total synthesis of garsubellin A.

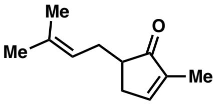

Enone **SI-1**: A 1 L flame-dried round bottom flask was charged with 2-methylcyclopent-2-en-1-one (6.0 mL, 61.2 mmol, 1.0 equiv.). The reaction vessel was evacuated and backfilled with nitrogen and dry THF (500 mL) was added. The flask was cooled to -78 °C and freshly prepared LDA (0.5 M in THF, 135 mL, 67.5 mmol, 1.1 equiv.) was added dropwise. The resulting solution was stirred at -78 °C for 15 minutes and then prenyl bromide (9 mL, 77.5 mmol, 1.3 equiv.) was added. The reaction mixture was slowly warmed to room temperature and stirred overnight and then quenched by the addition of saturated aqueous NH_4Cl solution (500 mL). The mixture was extracted with EtOAc (500 mL), washed with brine, (500 mL), dried over $MgSO_4$ and concentrated *in vacuo*. The crude product was purified by column chromatography (3% EtOAc in hexanes) to afford enone **SI-1** (5.3 g, 53% yield) as a yellow oil: ^{1}H NMR (700 MHz, $CDCl_3$) δ 7.27 (m, 1H), 5.05 (m, 1H), 2.66 (dddd, *J* = 18.8, 8.8, 4.6, 2.2 Hz, 1H), 2.48 (m, 1H), 2.38 (dddd, *J* = 8.9, 6.6, 4.5, 2.2 Hz, 1H), 2.20 (dq, *J* = 18.8, 2.4 Hz, 1H), 2.09 (dt, *J* = 14.4, 8.3 Hz, 1H), 1.77 (td, *J* = 2.2, 1.4 Hz, 3H), 1.68 (s, 3H), 1.61 (s, 3H); ^{13}C NMR (176 MHz, $CDCl_3$) δ 212.0, 157.2, 141.5, 133.8, 121.1, 45.3, 32.9, 29.9, 26.0, 18.0, 10.5; IR (thin film) vmax 3420, 3380, 2974, 2922, 1699, 1637, 1441, 1376, 1336, 1230, 1155, 1071 cm^{-1}; HRMS (ESI$^+$) *m/z* calcd. for $C_{11}H_{17}O$ [M+H]$^+$: 165.1274, found: 165.1274.

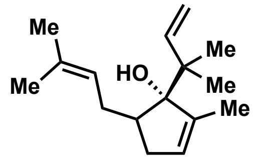

Alcohol **47**: A 250 mL flame-dried round bottom flask was charged with enone **SI-1** (2.3 g, 14 mmol, 1.0 equiv.). The reaction flask was evacuated and backfilled with nitrogen followed by the addition of dry THF (120 mL). Freshly prepared prenylmagnesium chloride (0.67 M, 50 mL, 33.5 mmol, 2.4 equiv.) was then added and the resulting mixture stirred at room temperature for 2 hours. After this period, the reaction was quenched by the addition of saturated aqueous $NaHCO_3$ solution (100 mL), extracted with EtOAc (3 x 200 mL), and concentrated *in vacuo*. The crude product was purified column chromatography on neutral alumina (10% EtOAc in hexanes) to afford allylic alcohol **47** (3.2 g, 98% yield) as a colorless oil: ^{1}H NMR (700 MHz, $CDCl_3$) δ 6.12 (dd, *J* = 18.0, 10.5 Hz, 1H), 5.51 (dt, *J* = 3.1, 1.6 Hz, 1H), 5.13 (tdt, *J* = 6.3, 2.9, 1.4 Hz, 1H), 5.04 (dd, *J* = 10.5, 1.5 Hz, 1H), 5.04

(dd, J = 18.0, 1.5 Hz, 1H), 2.38 – 2.25 (m, 2H), 2.21 – 2.15 (m, 1H), 1.95 – 1.81 (m, 2H), 1.74 (dt, J = 3.6, 1.8 Hz, 3H), 1.68 (s, 3H), 1.62 (s, 3H), 1.06 (s, 3H), 1.04 (s, 3H); ^{13}C NMR (176 MHz, $CDCl_3$) δ 146.1, 141.9, 132.7, 129.1, 123.8, 112.5, 89.6, 45.5, 43.3, 36.7, 30.7, 26.1, 23.0, 22.4, 18.2, 15.5; IR (thin film) νmax 3533, 3508, 3080, 3033, 2966, 2920, 2854, 1634, 1445, 1431, 1376, 1362, 1343, 1289, 1174 cm^{-1}; HRMS (EI) *m/z* calcd. for $C_{16}H_{26}O$ $[M]^+$: 234.1984, found: 234.1982.

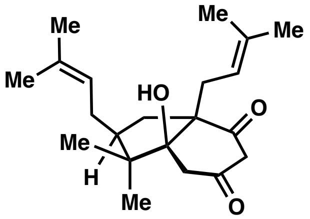

Diketone **49**: *i.* A 20 mL flame-dried reaction tube was charged with KH (205 mg, 5.13 mmol, 3.0 equiv.) and 18-crown-6 (1.35 g, 5.13 mmol, 3.0 equiv.). The tube was evacuated and backfilled with nitrogen (three times in total) and THF (5 mL) added. Allylic alcohol **47** (400 mg, 1.7 mmol, 1.0 equiv.) in 1 mL of THF was then added and the resulting solution was stirred at room temperature for 2 hours. After this period, the resulting mixture was transferred to a 100 mL flame-dried round bottom flask that contained a homogenous solution of $MgBr_2$ (1.57 g, 8.54 mmol, 5.0 equiv.) in THF (20 mL) preheated to 50 °C. The resulting brown solution was stirred for 10 mins and then MeI (0.53 mL, 8.54 mmol, 5.0 equiv.) was added. After two hours of stirring at 50 °C, the reaction mixture was slowly cooled to room temperature overnight. The reaction was quenched by the addition of saturated aqueous NH_4Cl solution (25 mL) and extracted with EtOAc (3 x 30 mL). The crude mixture was purified by column chromatography (3% EtOAc in hexanes) to afford an inseparable mixture of the desired methylated ketone (**48**) and small amounts of the C-5 methylated isomer and doubly alkylated product as a colorless oil (362 mg, 75% purity as determined by ^{1}H NMR, 65% yield) which were used directly in the next step. *ii.* A 20 mL flame-dried reaction tube was charged with **48** (250 mg, 75% purity, 0.76 mmol, 1 equiv) and the reaction vessel evacuated and backfilled with nitrogen (three times in total). Degassed THF (12.5 mL) and Et_2O (12.5 mL) were added and the reaction vessel cooled to -78 °C. Freshly prepared lithium 2,2,6,6-tetramethylpiperidide (0.45 M in THF, 2 mL, 0.9 mmol, 1.2 equiv.) was added dropwise resulting in a light-yellow colored solution. The reaction mixture was stirred for 30 minutes at -78 °C and then warmed to 0 °C and stirred 60 minutes. After this period, the reaction mixture was cooled to -40 °C and freshly distilled diketene (71 µL, 0.92 mmol, 1.2 equiv.) was added rapidly in one portion resulting in a bright yellow colored solution. The reaction vessel was maintained at this temperature for 90 minutes and then quenched by the addition of aqueous 1 M HCl (20 mL)

at this temperature. The reaction mixture was extracted with EtOAc (3 x 35 mL) and the combined organic layers were washed with brine, dried over $MgSO_4$, and concentrated *in vacuo*. The crude residue was purified by column chromatography (20% EtOAc in hexanes) to afford the annulated product **49** (114 mg, 44% yield) as a red/orange solid: m.p = 109 °C; ^{1}H NMR (700 MHz, $CDCl_3$) δ 5.12 (m, 1H), 4.92 (m, 1H), 3.64 (d, J = 18.2 Hz, 1H), 3.12 (d, J = 18.2 Hz, 1H), 2.76 (dd, J = 13.7, 7.2 Hz, 1H), 2.64 (d, J = 15.2 Hz, 1H), 2.53 (dd, J =15.1, 7.3 Hz, 1H), 2.41 (dd, J = 15.1, 7.1 Hz, 1H), 2.37 (d, J = 15.2 Hz, 1H), 2.22 – 2.05 (m, 1H), 1.85 (s, 1H), 1.80 (ddd, J = 14.4, 9.6, 7.3 Hz, 1H), 1.69 (s, 6H), 1.59 (dd, J = 4.0, 1.3 Hz, 6H), 1.37 (dddd, J = 11.4, 10.1, 7.2, 4.0 Hz, 1H), 1.23 (dd, J = 13.7, 12.2 Hz, 1H), 0.96 (s, 3H), 0.87 (s, 3H); ^{13}C NMR (176 MHz, $CDCl_3$) δ 206.3, 203.9, 135.2, 132.4, 122.9, 118.3, 83.7, 61.1, 51.9, 50.8, 48.2, 45.2, 35.2, 35.0, 29.0, 26.1, 26.0, 23.7, 18.2, 18.0, 17.9; IR (thin film) νmax 2971, 2915, 2880, 1732, 1703, 1602, 1452, 1415, 1377, 1325, 1223, 1145 cm^{-1}; HRMS (ESI-) *m/z* calcd. for $C_{21}H_{31}O_3$ [M-H]$^-$: 331.2279, found: 331.2274.

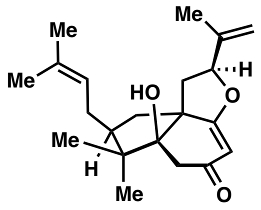

Vinylogous ester **52**: A 100 mL round bottom flask was charged with diketone **49** (100 mg, 0.3 mmol, 1.0 equiv.), $Pd(OAc)_2$ (10 mg, 0.045 mmol, 15 mol%) and $Cu(OAc)_2$ (60 mg, 0.33 mmol, 1.1 equiv.). 14 mL of DMSO and 6 mL of TMSOH were injected and the resulting green solution was allowed to stir at room temperature for 7 hours. The reaction was washed with water (3 x 40 mL) and then combined aqueous solutions extracted with diethyl ether (3 x 40 mL). The combined organic layers were dried over $MgSO_4$ and concentrated *in vacuo*. The crude product was purified by column chromatography (25% EtOAc in hexanes) to give vinylogous ester **52** as a foam (95 mg, 95% yield): m.p. = 140 °C; ^{1}H NMR (600 MHz, $CDCl_3$) δ 5.47 (s, 1H), 5.11 (s, 1H), 5.06 (m, 1H), 4.99 (s, 1H), 4.91 (dd, J = 11.2, 4.8 Hz, 1H), 2.67 (d, J = 17.4 Hz, 1H), 2.40 (d, J = 17.4 Hz, 1H), 2.35 (t, J = 11.6 Hz, 1H), 2.19 (m, 2H), 2.02 (m, 2H), 1.93 (m, 2H), 1.75 (s, 3H), 1.69 (s, 3H), 1.64 (m, 4H), 0.98 (d, J = 1.7 Hz, 6H); ^{13}C NMR (151 MHz, $CDCl_3$) δ 196.7, 183.9, 142.0, 132.9, 122.8, 114.0, 99.8, 85.7, 84.4, 54.5, 46.7, 46.5, 46.0, 41.7, 40.7, 30.9, 25.9, 23.6, 18.8, 18.1, 17.4; IR (thin film) νmax 2970, 2935, 1627, 1447, 1363, 1230, 1182, 1155 cm^{-1}; HRMS (ESI+) *m/z* calcd. for $C_{21}H_{31}O_3$ [M+H]$^+$: 331.2268, found: 331.2268.

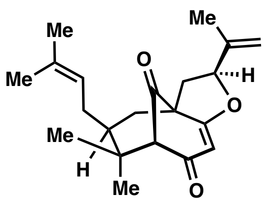

Polycycle **55**: To a 20 mL reaction tube containing vinylogous ester **52** (100 mg, 0.30 mmol, 1.0 equiv.) under nitrogen was added a solution of KOH (5 M in methanol, 6 mL) resulting reddish orange colored solution. The mixture was cooled to -10 °C and PIDA (270 mg, 0.838 mmol, 2.7 equiv.) added as a solid. The reaction mixture was maintained at a temperature of -10–0 °C for 1 hour, quenched with saturated aqueous $NaHCO_3$ (10 mL), and extracted with EtOAc (3 x 20 mL). The combined organic layers were washed with brine, dried over $MgSO_4$, and concentrated *in vacuo*. The crude material was purified by column chromatography (10% EtOAc in hexanes) to give **55** as a pale yellow solid (75 mg, 75% yield): m.p. = 137 °C; ^{1}H NMR (600 MHz, $CDCl_3$) δ 5.77 (s, 1H), 5.12 (m, 2H), 5.01 (m, 2H), 2.82 (s, 1H), 2.51 (dd, *J* = 13.1, 11.3 Hz, 1H), 2.21 – 2.15 (m, 1H), 2.13 (dd, *J* = 13.7, 4.6 Hz, 1H), 1.90 (dd, *J* = 13.2, 5.5 Hz, 1H), 1.80 (m, 1H), 1.75 (m, 1H), 1.72 (s, 3H), 1.70 (s, 3H), 1.57 (s, 3H), 1.52 (m, 1H), 1.16 (s, 3H), 0.90 (s, 3H); ^{13}C NMR (150 MHz, $CDCl_3$) δ 204.4, 194.4, 178.8, 141.2, 133.7, 122.3, 114.5, 104.4, 87.6, 73.4, 60.0, 42.8, 40.5, 37.6, 33.6, 27.5, 26.8, 26.0, 20.7, 18.0, 17.2; IR (thin film) νmax 2969, 2933, 1735, 1651, 1624, 1364, 1250, 1199, 1177, 1055 cm^{-1}; HRMS (ESI-) *m/z* calcd. for $C_{21}H_{31}O_3$ $[M-H]^-$: 331.2279, found: 331.2274.

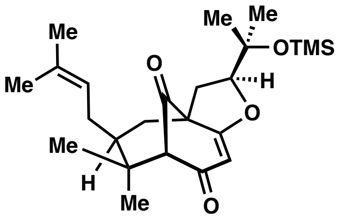

Polycycle **56**: A flame-dried reaction tube was charged with olefin **55** (18.6 mg, 0.06 mmol, 1.0 equiv.), (*S,S*)-(+)-*N,N'*-Bis(3,5-di-*tert*-butylsalicylidene)-1,2-cyclohexanediaminocobalt(II) (3.7 mg, 0.006 mmol, 10 mol%), and dry DMF (0.6 mL). Phenylsilane (8.3 μL, 0.067 mmol, 1.2 equiv.) was then added as a stock solution in DMF (0.2 mL) and the resulting red colored solution was saturated with O_2 by bubbling from a balloon for 10 minutes. The reaction mixture was then stirred at room temperature for 15 hours under an oxygen atmosphere (not bubbling). After that period, TMSCl (72 μL, 0.57 mmol, 10 equiv.), imidazole (39 mg, 0.57 mmol, 10 equiv.), and DMAP (1.4 mg, 0.011 mmol, 20 mol%) were added. The reaction mixture was stirred for 2 days at room temperature, quenched by the addition of saturated aqueous $NaHCO_3$ solution (5 mL), and extracted with EtOAc (3 x 5 mL). The combined organic layers were washed with brine, dried over $MgSO_4$, and concentrated *in vacuo*. The crude product was purified by column chromatography (5% EtOAc in hexanes) to give polycycle **56** as a white solid (16.8 mg, 72%

yield): m.p. = 82 °C; ^{1}H NMR (600 MHz, $CDCl_3$) δ 5.73 (s, 1H), 5.00 (m, 1H), 4.48 (dd, *J* = 10.4, 5.8 Hz, 1H), 2.80 (s, 1H), 2.65 (dd, *J* = 13.1, 10.4 Hz, 1H), 2.18 (m, 1H), 2.06 (dd, *J* = 13.7, 4.6 Hz, 1H), 1.79 (m, 1H), 1.72 (m, 1H), 1.70 (s, 3H), 1.67 (m, 1H), 1.57 (s, 3H), 1.49 (dd, *J* = 13.7, 12.2 Hz, 1H), 1.30 (s, 3H), 1.22 (s, 3H), 1.15 (s, 3H), 0.90 (s, 3H), 0.08 (s, 9H). ^{13}C NMR (150 MHz, $CDCl_3$) δ 204.4, 194.6, 179.5, 133.6, 122.4, 104.2, 91.6, 73.8, 73.4, 60.0, 42.6, 40.6, 38.4, 29.3, 27.5, 26.9, 26.7, 26.0, 25.9, 20.7, 18.0, 2.5; IR (thin film) vmax 2969, 2933, 1735, 1651, 1624, 1451, 1395, 1375, 1360, 1345, 1274, 1240, 1197, 1175, 1146 cm^{-1}; HRMS (ESI+) *m/z* calc'd for $C_{24}H_{39}O_4Si$ $[M+H]^+$: 419.2612, found: 419.2612.

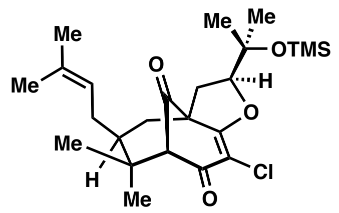

Chloride **57**. A 50 mL flame-dried flask was charged with compound **56** (125 mg, 0.30 mmol, 1.0 equiv.). The reaction vessel was evacuated and backfilled with nitrogen and this process repeated twice. THF (15 mL) was then added and the solution cooled to -78 °C. A solution of freshly prepared lithium 2,2,6,6-tetramethylpiperidide (0.50 M in THF, 1.23 mL, 0.62 mmol, 2.1 equiv.) was added dropwise resulting in a light-yellow colored solution. The reaction mixture was stirred for 60 minutes at -78 °C and then *p*-toluenesulfonyl chloride (120 mg, 0.63 mmol, 2.1 equiv.) was added as a solution in THF. The reaction mixture was stirred for 15 minutes at -78 °C, warmed to 0 °C and stirred 15 minutes, and then quenched by the addition of saturated aqueous $NaHCO_3$ solution (10 mL). The reaction mixture was extracted with EtOAc (3 x 20 mL) and the combined organic layers were washed with brine, dried over $MgSO_4$, and concentrated *in vacuo*. The crude product was purified by column chromatography (50% DCM in PhMe *then* 20% EtOAc in hexanes) affording **57** (134 mg, 98% yield) as a white solid: m.p. = 115 °C; ^{1}H NMR (400 MHz, $CDCl_3$) δ 5.48 (m, 1H), 4.61 (dd, J = 10.1, 6.0 Hz, 1H), 3.01 (s, 1H), 2.80 (dd, J = 13.1, 10.1 Hz, 1H), 2.20 (m, 1H), 2.13 (dd, *J* = 13.6, 3.9 Hz, 1H), 1.76 (dd, *J* = 13.2, 6.0 Hz, 1H), 1.70 (s, 3H), 1.69 (m, 2H), 1.56 (s, 3H), 1.51(m, 1H), 1.36 (s, 3H), 1.24 (s, 3H), 1.16 (s, 3H), 0.90 (s, 3H), 0.08 (s, 9H). ^{13}C NMR (100 MHz, $CDCl_3$) δ 202.1, 187.2, 173.2, 133.9, 122.0, 106.8, 92.9, 73.8, 73.4, 61.5, 43.0, 40.6, 38.6, 29.9, 27.5, 26.7, 26.0, 25.9, 20.6, 18.0, 2.4; IR (thin film) vmax 2967, 2934, 1739, 1669, 1615, 1452, 1385, 1366, 1344, 1249, 1216, 1177, 1051 cm^{-1}; HRMS (ESI+) *m/z* calc'd for $C_{24}H_{38}O_4ClSi$ $[M+H]^+$: 453.2222, found: 453.2226.

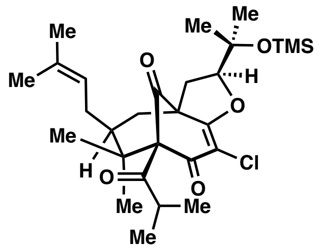

Triketone **58**. Three 20 mL flame-dried reaction tubes were charged with compound **57** (3 x 40 mg, 0.27 mmol, 1.0 equiv.). The reaction vessels were evacuated and backfilled with nitrogen (three times in total) followed by the addition of THF (1 mL each). The reaction mixtures were cooled to -78 °C and a freshly prepared solution of lithium 2,2,6,6-tetramethylpiperidide (0.45 M in THF, 0.64 mL, 0.28 mmol, 3.2 equiv.) was added dropwise to each vessel resulting in a light brown colored solution. The reaction mixtures were stirred for 10 minutes at -78°C, warmed to 0 °C and stirred for 5 minutes, and then re-cooled to -78 °C. Isobutyryl chloride (47 µL, 0.44 mmol, 5 equiv.) was then added dropwise to each vessels at -78 °C. The reaction mixtures were slowly warmed to -5 °C over the course of 60 minutes at which point they were quenched with saturated aqueous $NaHCO_3$ solution (5 mL). The contents of the tubes were combined and extracted with EtOAc (3 x 20 mL). The combined organic layers were washed with brine, dried over $MgSO_4$, and concentrated *in vacuo*. The crude product was purified by column chromatography (2% → 10% Et_2O in hexanes) to afford **58** (90 mg, 65% yield) as a yellow oil: ^{1}H NMR (400 MHz, $CDCl_3$) δ 4.93 (m, 1H), 4.64 (dd, *J* = 9.9, 6.2 Hz, 1H), 2.93 (dd, *J* = 13.2, 9.9 Hz, 1H), 2.18 (m, 1H), 2.10 (dd, *J* = 11.7, 20.6 Hz, 1H), 2.01 (hept, *J* = 6.5 Hz, 1H), 1.80 (dd, *J* = 13.2, 6.2 Hz, 1H), 1.72 (q, *J* = 8.6 Hz, 1H), 1.68 (s, 3H), 1.58 (m, 1H), 1.55 (s, 3H), 1.43 (s, 3H), 1.31 (m, 1H), 1.27 (s, 3H), 1.24 (s, 3H), 1.08 (d, *J* = 6.6 Hz, 3H), 1.07 (s, 3H), 1.02 (d, *J* = 6.6 Hz, 3H), 0.08 (s, 9H). ^{13}C NMR (100 MHz, $CDCl_3$) δ 207.8, 202.7, 187.1, 172.8, 133.9, 122.1, 106.8, 93.1, 82.9, 73.8, 61.8, 47.0, 42.6, 42.5, 39.5, 30.2, 26.8, 26.7, 26.4, 26.0, 22.7, 21.6, 20.5, 18.1, 16.3, 2.3; IR (thin film) νmax 2973, 2929, 1736, 1667, 1619, 1445, 1370, 1345, 1251, 1222, 1178, 1051 cm^{-1}; HRMS (ESI+) *m/z* calc'd for $C_{28}H_{44}O_5ClSi$ $[M+H]^+$: 523.2641, found: 523.2642.

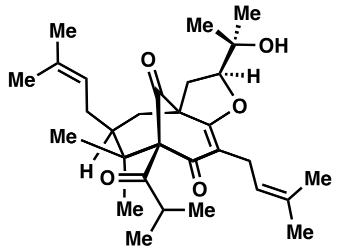

Garsubellin A (**8**) and TMS-garsubellin A (**59**). A 10 mL flame-dried reaction tube under nitrogen was charged with compound **58** (10 mg, 0.02 mmol, 1.0 equiv.) and prenylBpin (20 mg, 0.1 mmol, 5.3 equiv.). CPhos (2-Dicyclohexylphosphino-2',6'-bis(*N,N*-dimethylamino)biphenyl (2.2 mg, 0.005 mmol, 25 mol%), and $[Pd(allyl)Cl]_2$ (0.7 mg, 0.002 mmol, 10 mol%) in 0.4 mL of dioxane was injected along with 0.4 mL of an aqueous K_3PO_4 solution (65 mg, 0.30 mmol, 16 equiv. of K_3PO_4). The sealed reaction vessel was heated to 110 °C for 4 hours, cooled to room temperature, quenched with saturated

aqueous $NaHCO_3$ (5 mL), and extracted with EtOAc (3 x 20 mL). The combined organic layers were washed with brine, dried over $MgSO_4$, and concentrated *in vacuo*. The crude product was purified by column chromatography (20 mol% EtOAc in hexanes) to give **8** as a colorless oil (5.5 mg, 57 % yield). Less polar fractions were combined and re-chromatographed (25% DCM in PhMe) to give **59** as a colorless oil (1.8 mg, 17% yield): Data for **8**: ^{1}H NMR (700 MHz, C_6D_6) δ 5.40 (dd, J = 7.4, 5.9 Hz, 1H), 4.96 (dddd, J = 7.3, 5.9, 2.9, 1.4 Hz, 1H), 3.91 (dd, J = 10.7, 5.8 Hz, 1H), 3.39 (dd, J = 14.2, 7.2 Hz, 1H), 3.21 (dd, J = 14.2, 7.6 Hz, 1H), 2.73 (dd, J = 13.0, 10.7 Hz, 1H), 2.26 (hept, J = 6.5 Hz, 1H), 2.09 (m, 1H), 1.93 (dd, J = 13.6, 4.5 Hz, 1H), 1.75 (dddd, J = 12.5, 10.5, 4.6, 2.8 Hz, 1H), 1.70 (d, J = 1.3 Hz, 3H), 1.61 (s, 6H), 1.58 (s, 3H), 1.55 (m, 1H), 1.45 (s, 3H), 1.37 (d, J = 6.5 Hz, 3H), 1.31 (d, J = 6.5 Hz, 3H), 1.28 (m, 1H), 1.26 (m, 1H), 1.25 (s, 3H), 1.04 (s, 1H), 0.93 (s, 3H), 0.77 (s, 3H); ^{13}C NMR (176 MHz, C_6D_6) δ 208.5, 204.7, 192.9, 173.2, 133.2, 132.5, 123.3, 122.1, 116.7, 90.2, 82.7, 70.3, 59.9, 46.7, 43.1, 42.8, 39.1, 30.3, 27.1, 26.4, 26.0, 25.8, 24.5, 23.2, 22.7, 22.0, 20.9, 17.9, 17.9, 16.5; IR (thin film) νmax 3444, 2967, 2925, 2854, 1732, 1664, 1622, 1561, 1501, 1451, 1365, 1250, 1213, 1176, 1099, 1054 cm^{-1}; HRMS (ESI-) *m/z* calc'd for $C_{30}H_{43}O_5$ [M-H]$^-$: 483.3116, found: 483.3127.

Data for **59**: ^{1}H NMR (700 MHz, $CDCl_3$) δ 5.07 (m, 1H), 4.94 (m, 1H), 4.48 (dd, J = 10.0, 6.2 Hz, 1H), 3.14 (dd, J = 14.2, 7.8 Hz, 1H), 3.01 (dd, J = 14.3, 6.8 Hz, 1H), 2.76 (dd, J = 13.1, 10.0 Hz, 1H), 2.15 (d, J = 14.3 Hz, 1H), 2.02 (dd, J = 12.7, 3.7 Hz, 1H), 1.97 (hept, J = 6.6 Hz, 1H), 1.72 (m, 4H), 1.68 (s, 3H), 1.66 (m, 1H), 1.61 (s, 3H), 1.55 (s, 3H), 1.543 (m, 1H), 1.48 (m, 1H), 1.35 (s, 3H), 1.27 (s, 3H), 1.25 (s, 3H), 1.07 (d, J = 6.5 Hz, 3H), 1.04 (s, 3H), 0.97 (d, J = 6.6 Hz, 3H), 0.09 (s, 9H); ^{13}C NMR (176 MHz, $CDCl_3$) δ 209.3, 204.9, 193.0, 173.8, 133.5, 132.4, 122.6, 121.3, 116.1, 91.0, 82.2, 74.1, 59.8, 46.3, 42.6, 42.1, 39.5, 30.1, 29.9, 26.7, 26.6, 26.2, 26.0, 25.8, 22.9, 22.4, 21.5, 20.6, 18.0, 18.0, 16.3, 2.4; IR (thin film) νmax 2926, 2856, 1733, 1661, 1622, 1455, 1372, 1249, 1231, 1215, 1177, 1121, 1099, 1052 cm^{-1}; HRMS (ESI-) *m/z* calc'd for $C_{33}H_{52}O_5ClSi$ [M+Cl]$^-$: 591.3278, found: 591.3276.

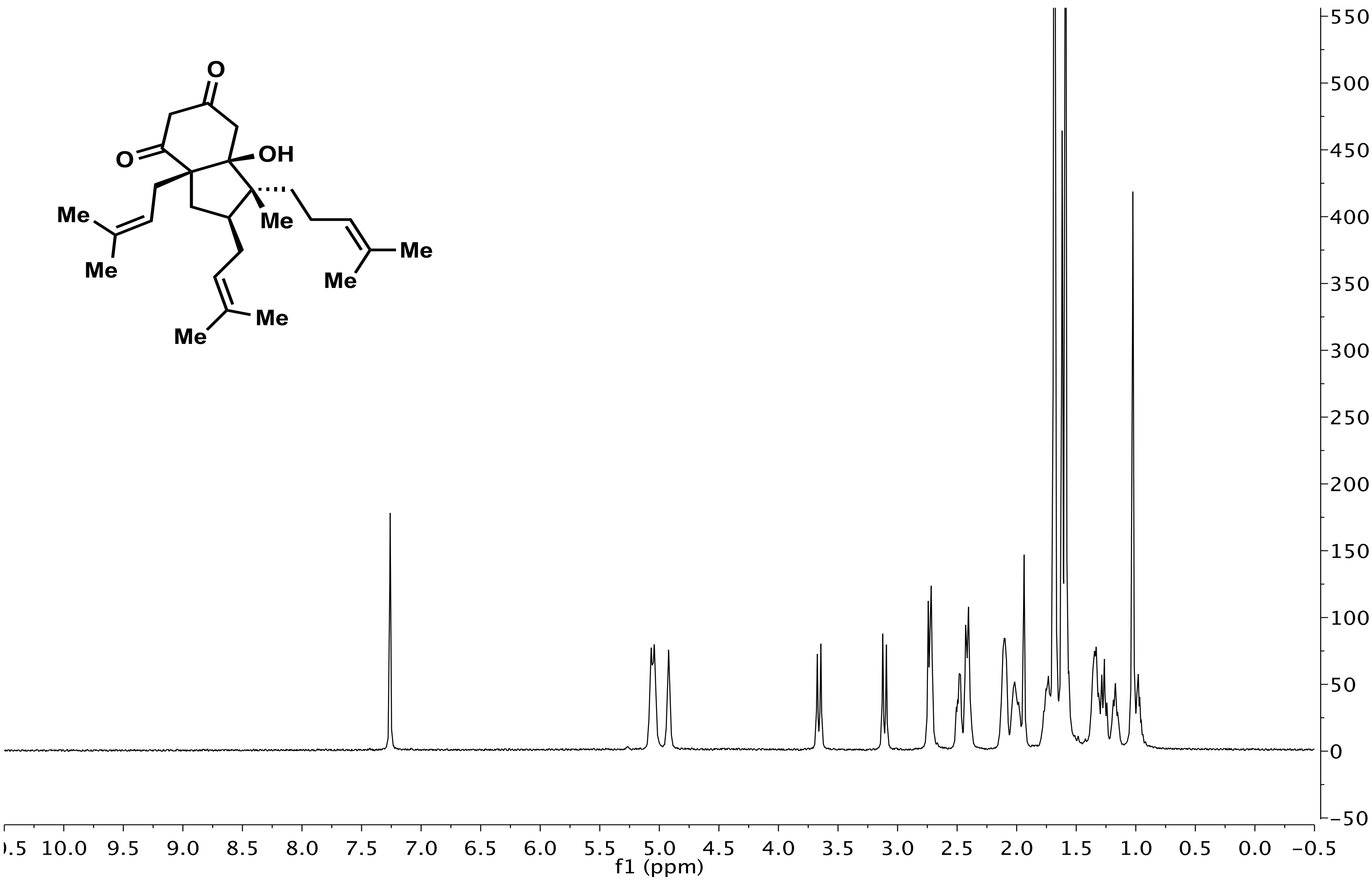

Supplementary Figure 3. ^{1}H NMR of **17**

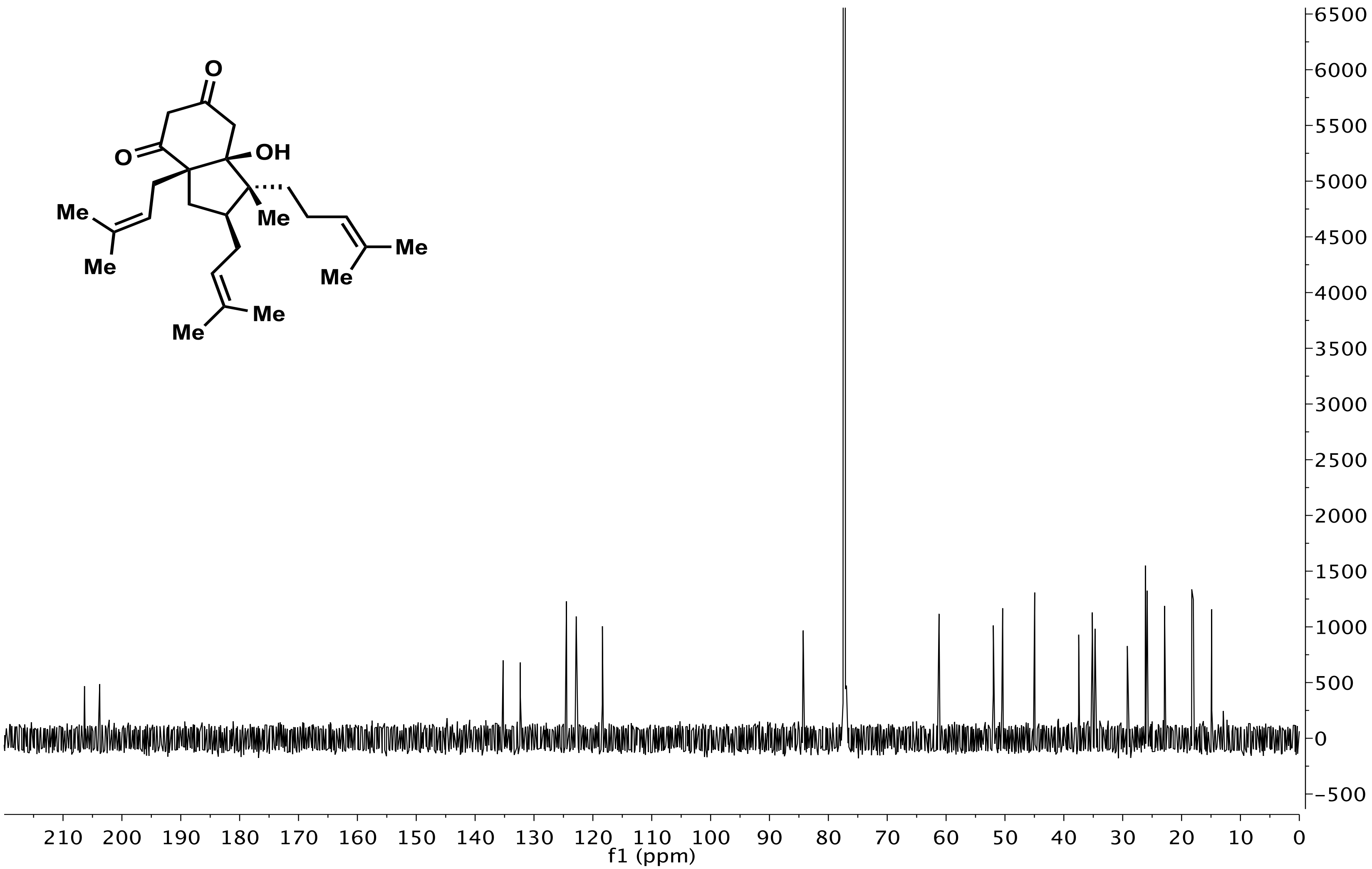

Supplementary Figure 4. ^{13}C NMR of **17**

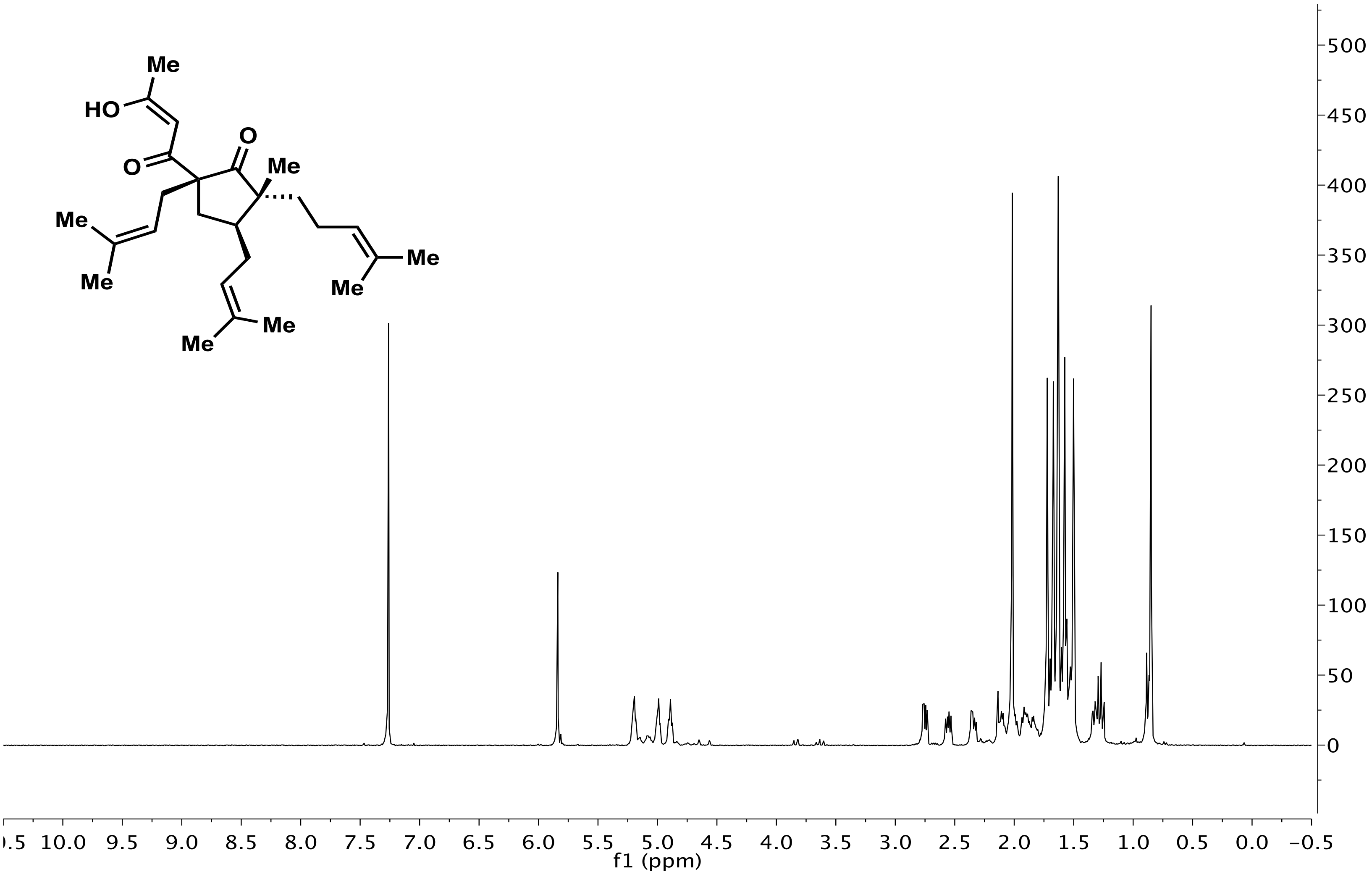

Supplementary Figure 5. ^{1}H NMR of **18**

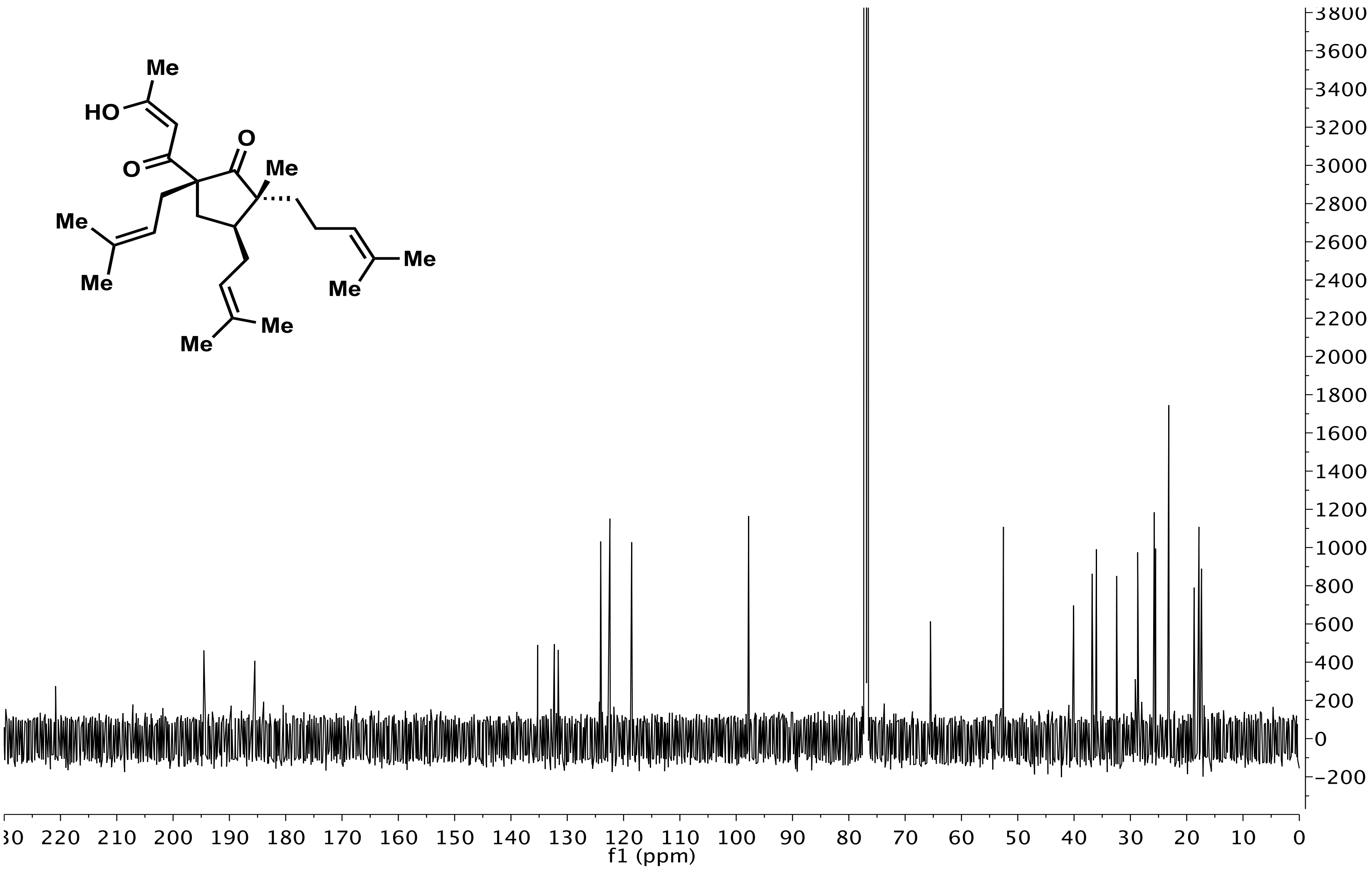

Supplementary Figure 6. ^{13}C NMR of **18**

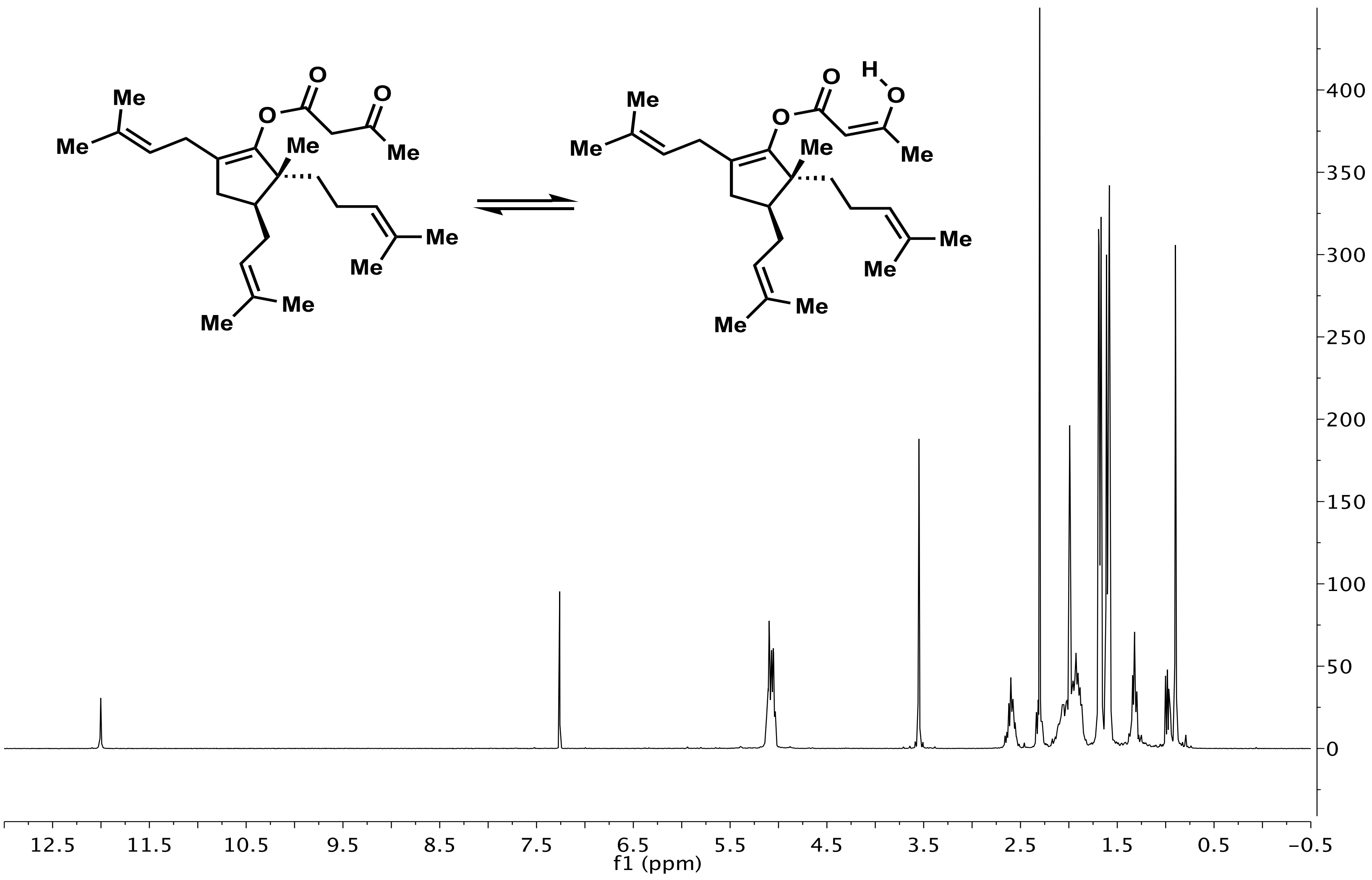

Supplementary Figure 7. ^{1}H NMR of **19**

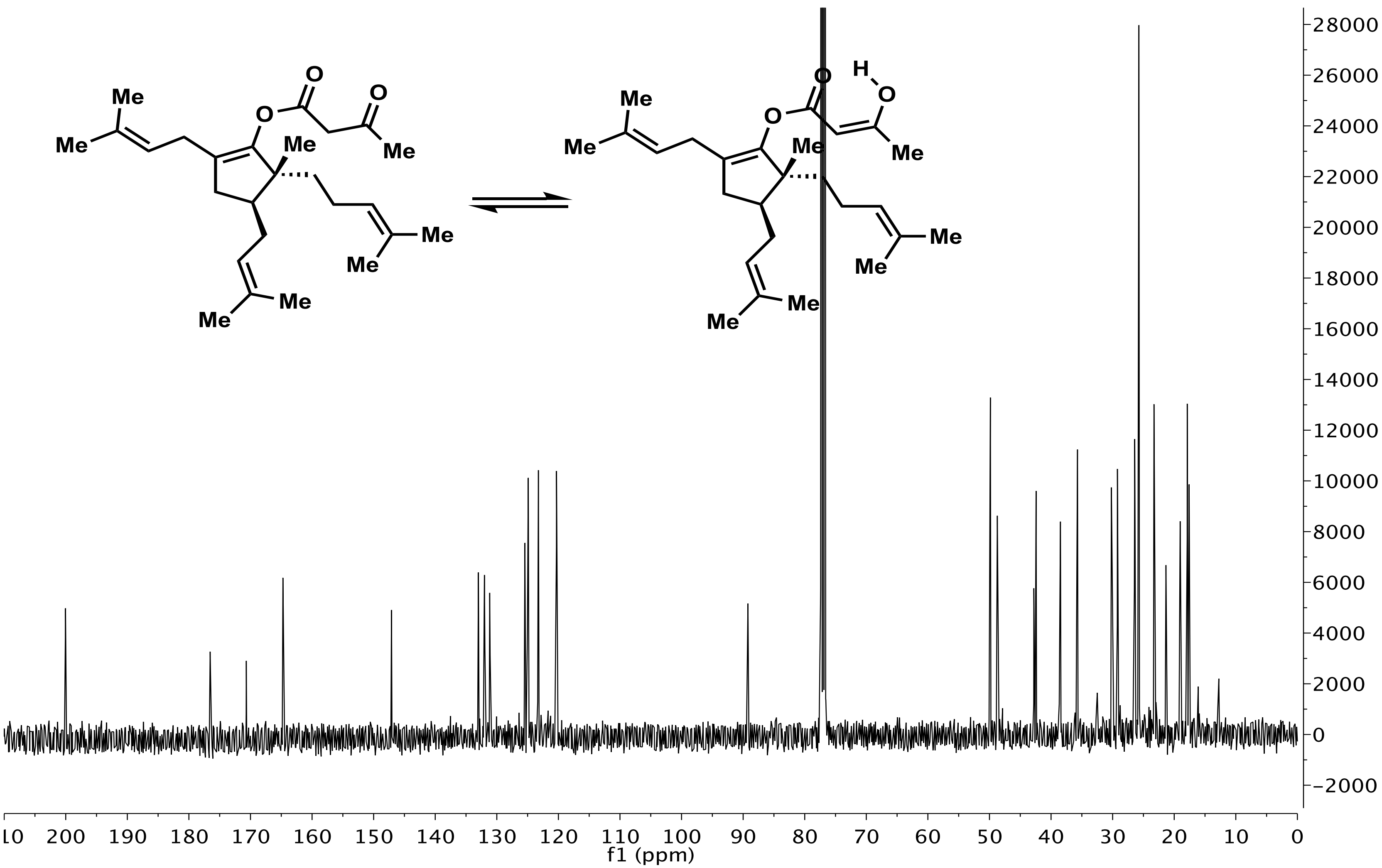

Supplementary Figure 8. ^{13}C NMR of **19**

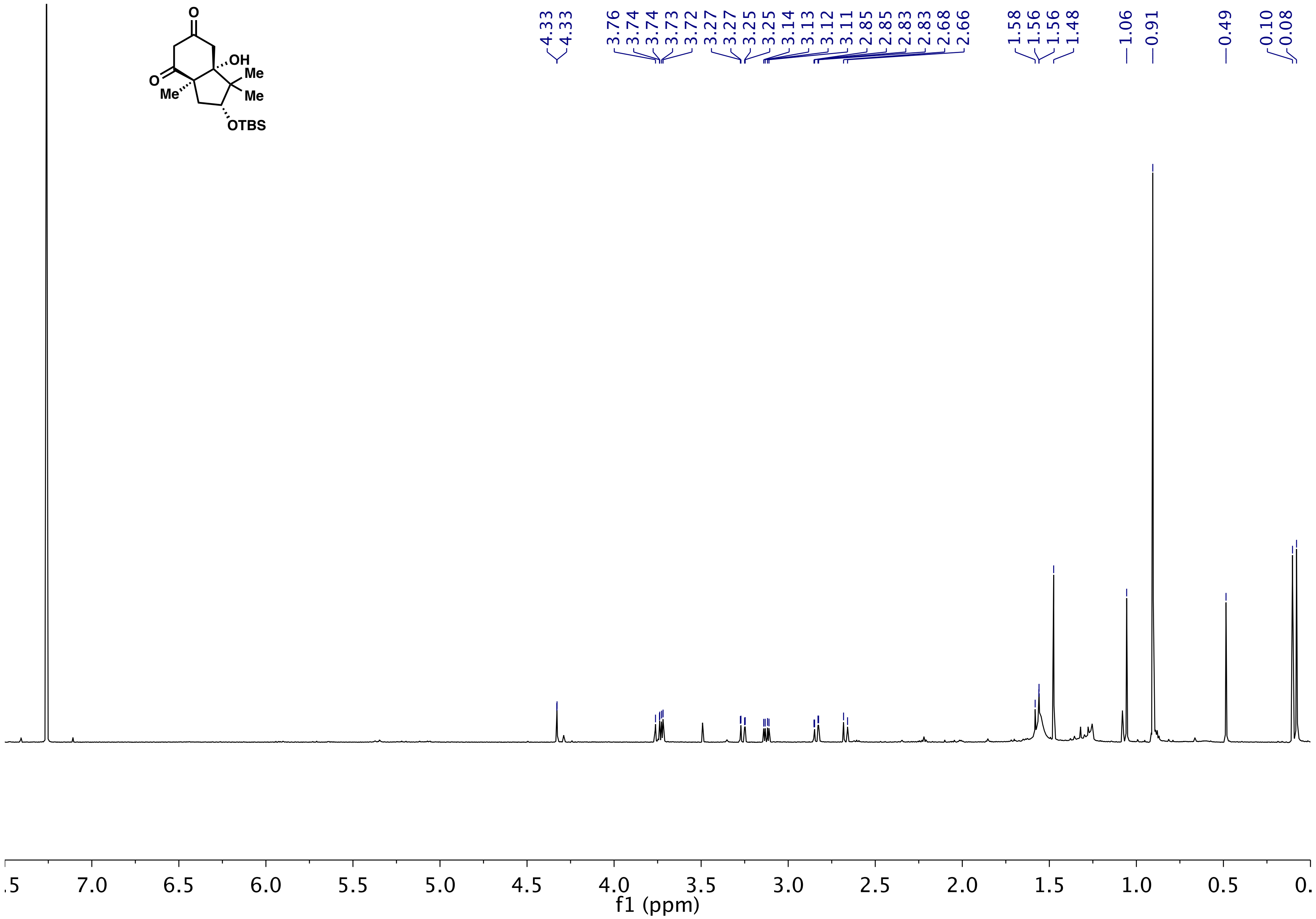

Supplementary Figure 9. ^{1}H NMR of **26**

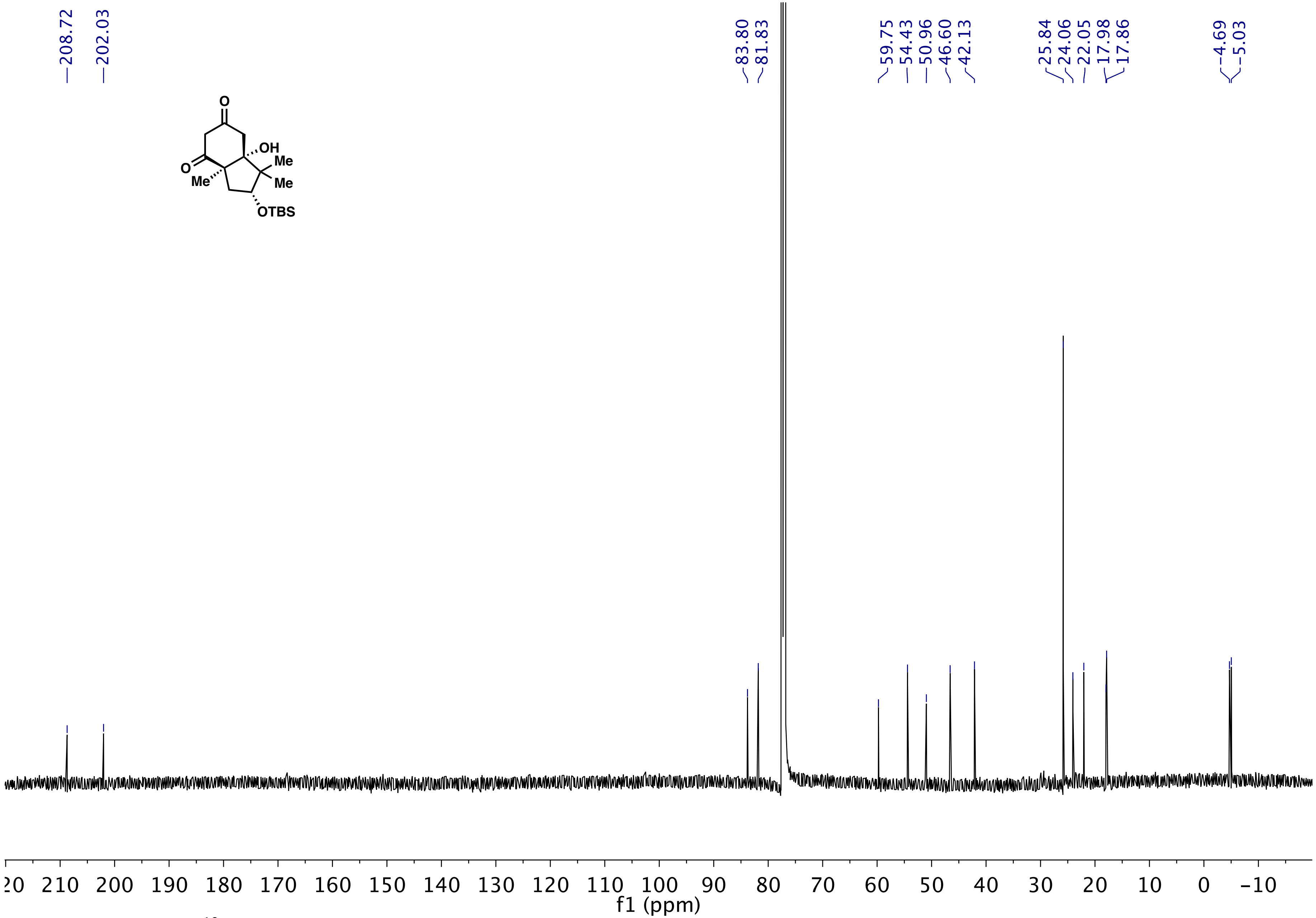

Supplementary Figure 10. ^{13}C NMR of **26**

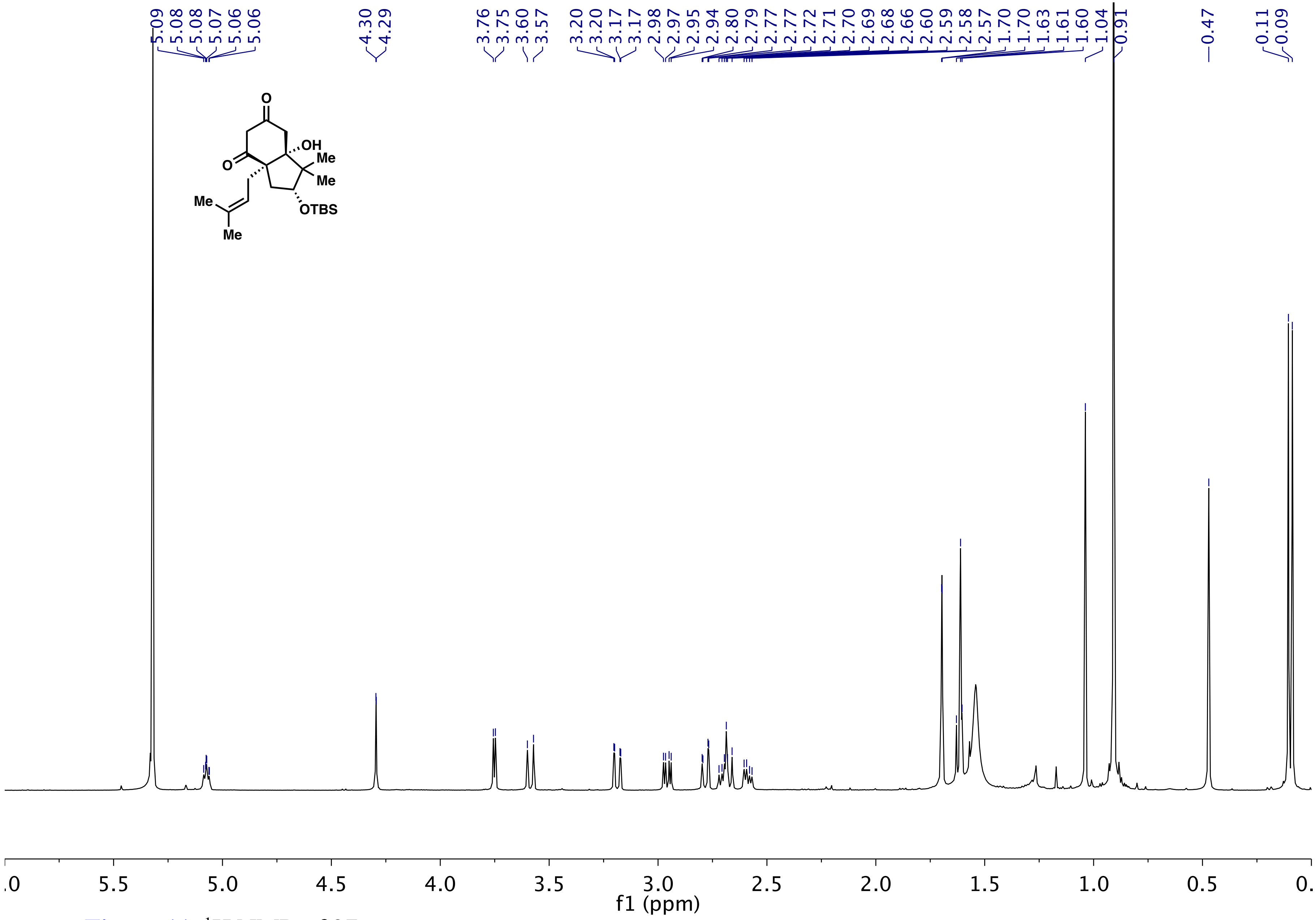

Supplementary Figure 11. ¹H NMR of **27**

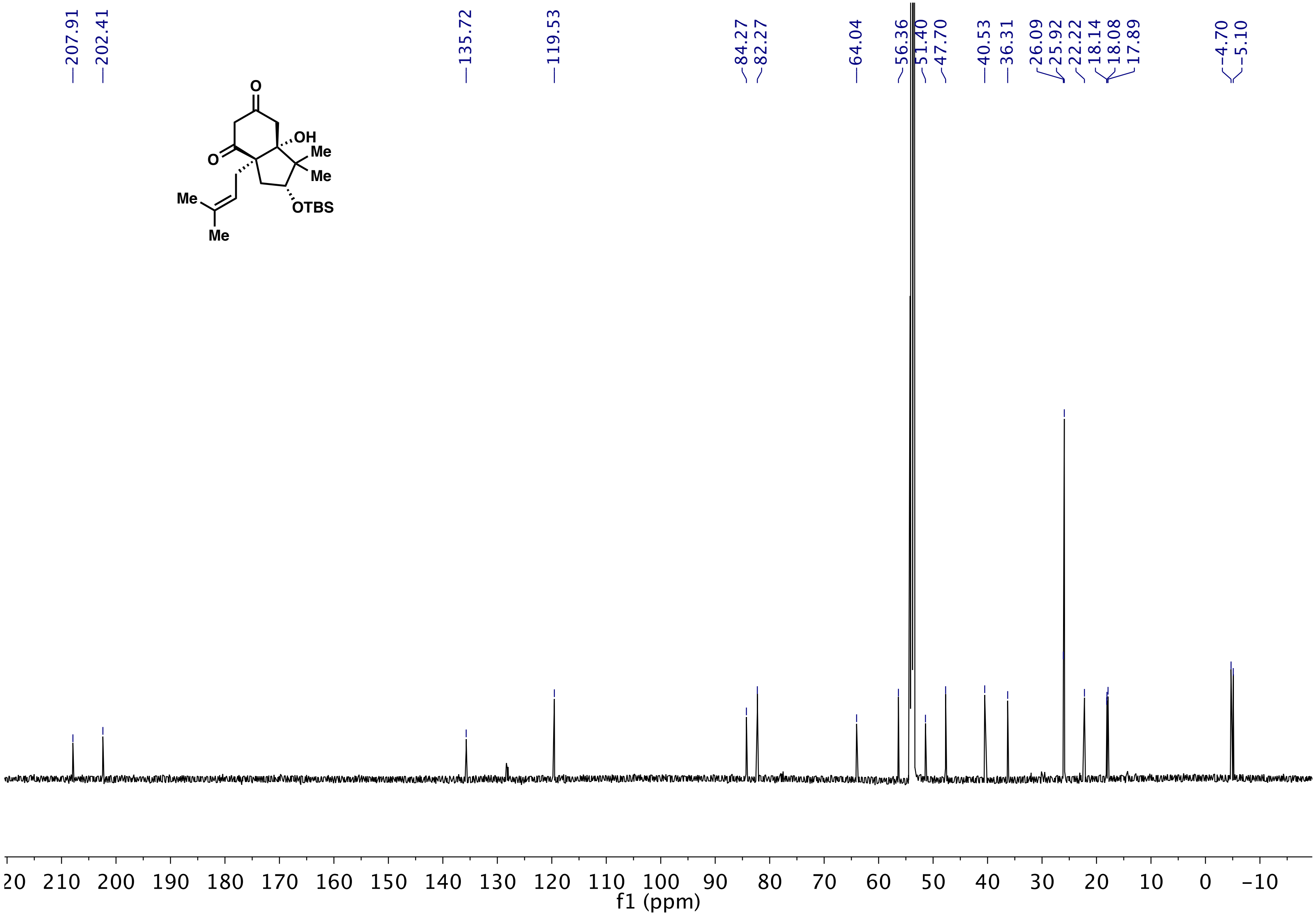

Supplementary Figure 12. ^{13}C NMR of **27**

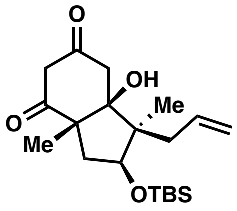

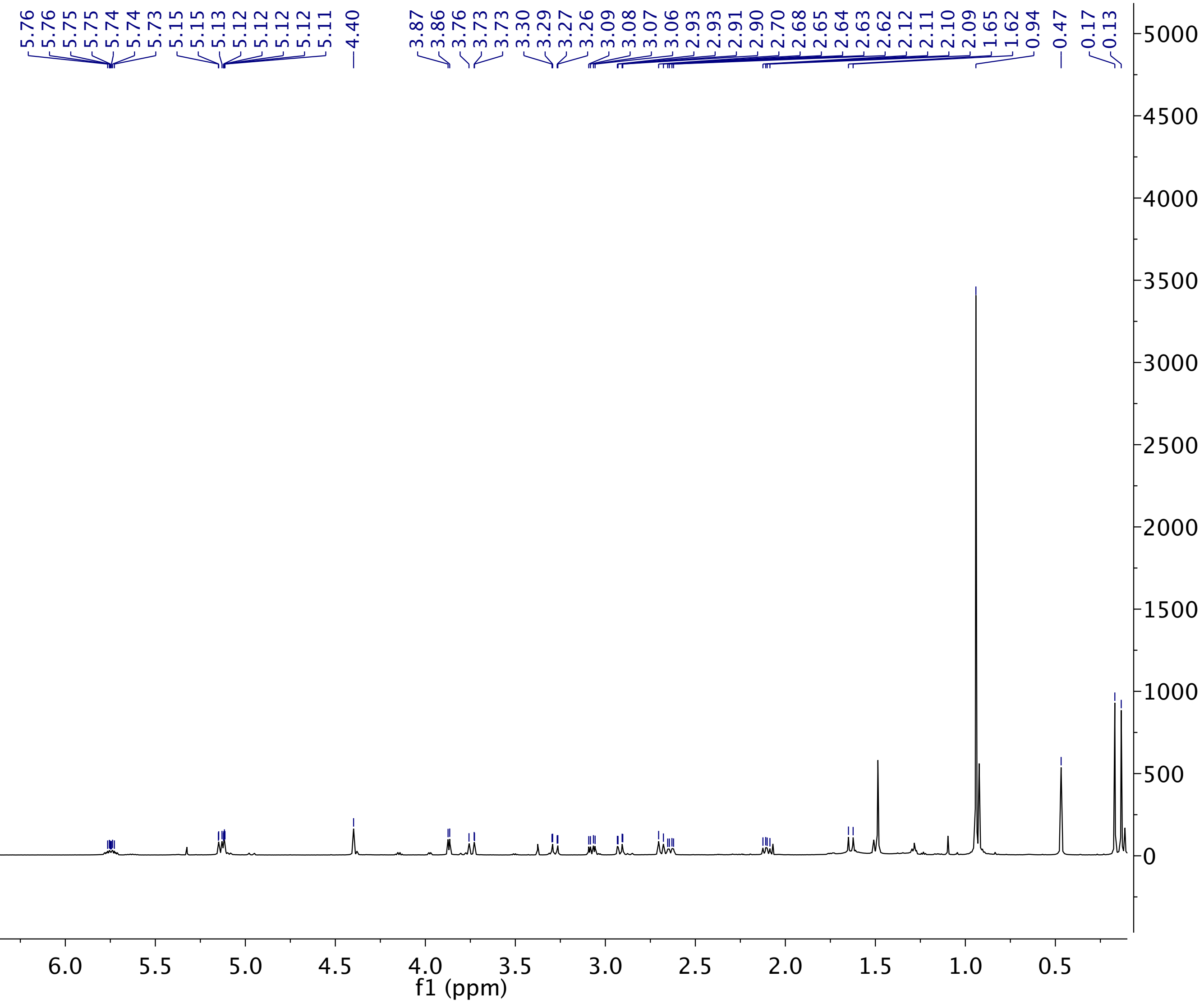

Supplementary Figure 13. ^{1}H NMR of **28**

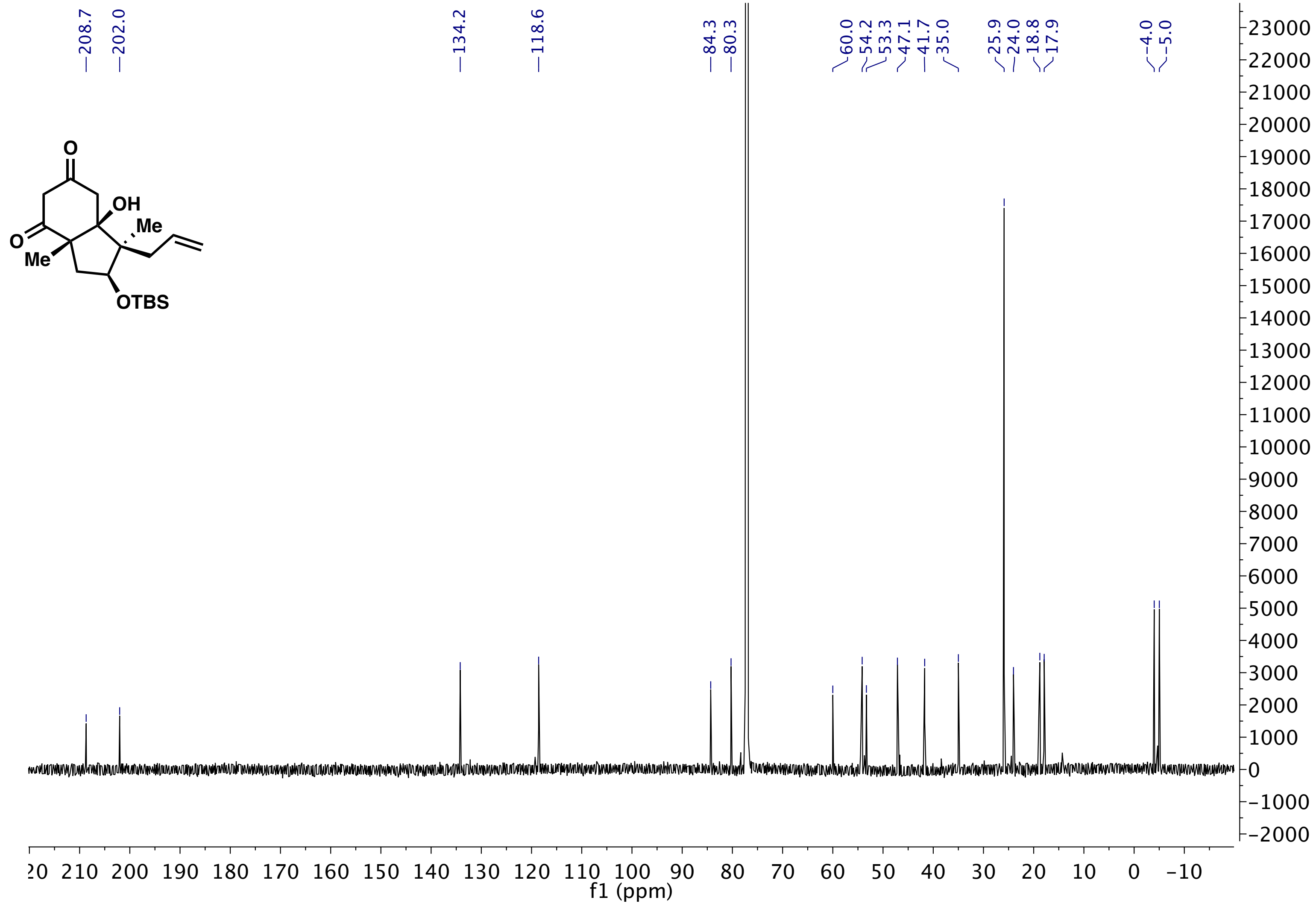

Supplementary Figure 14. ^{13}C NMR of **28**

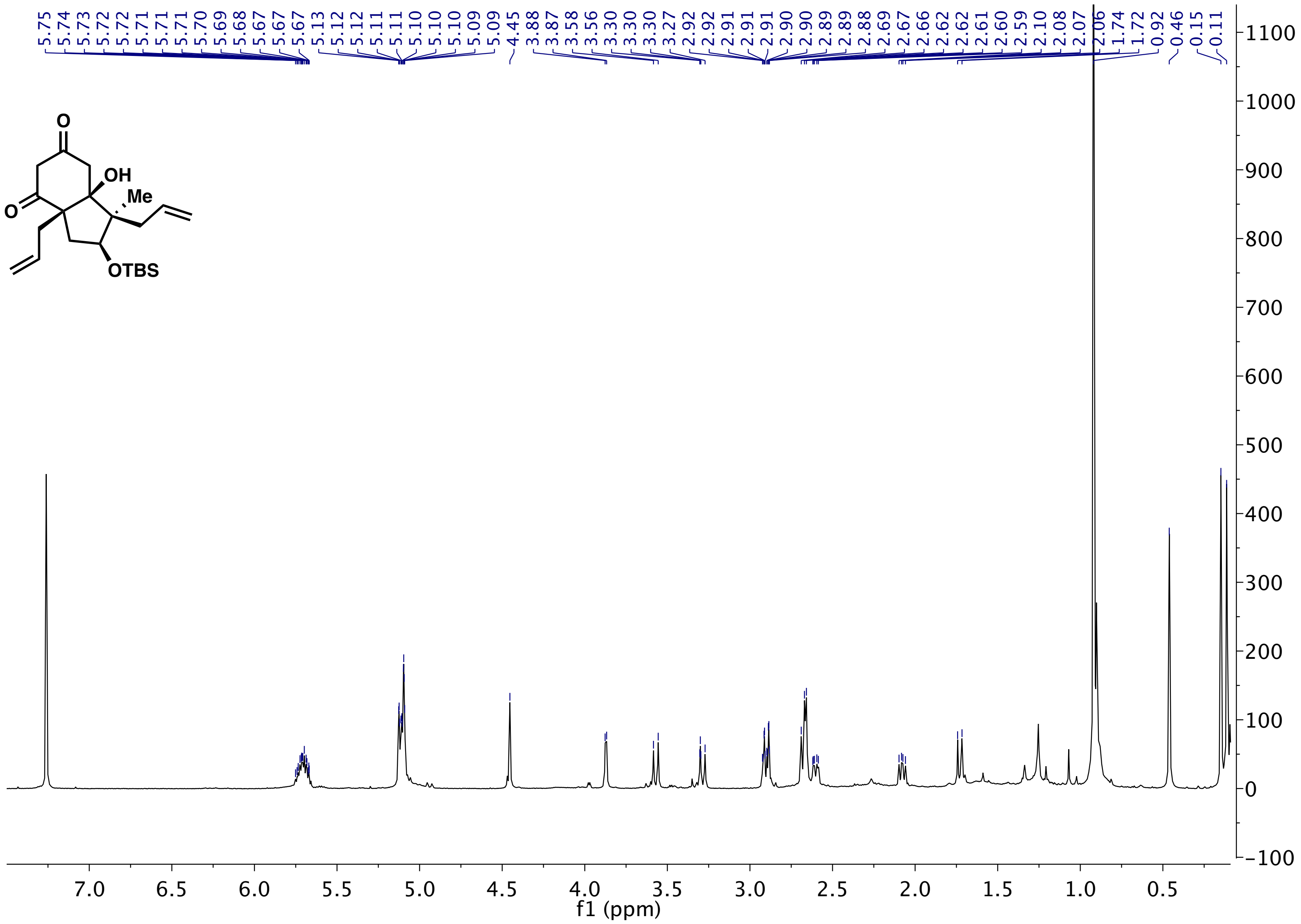

Supplementary Figure 15. ^{1}H NMR of **29**

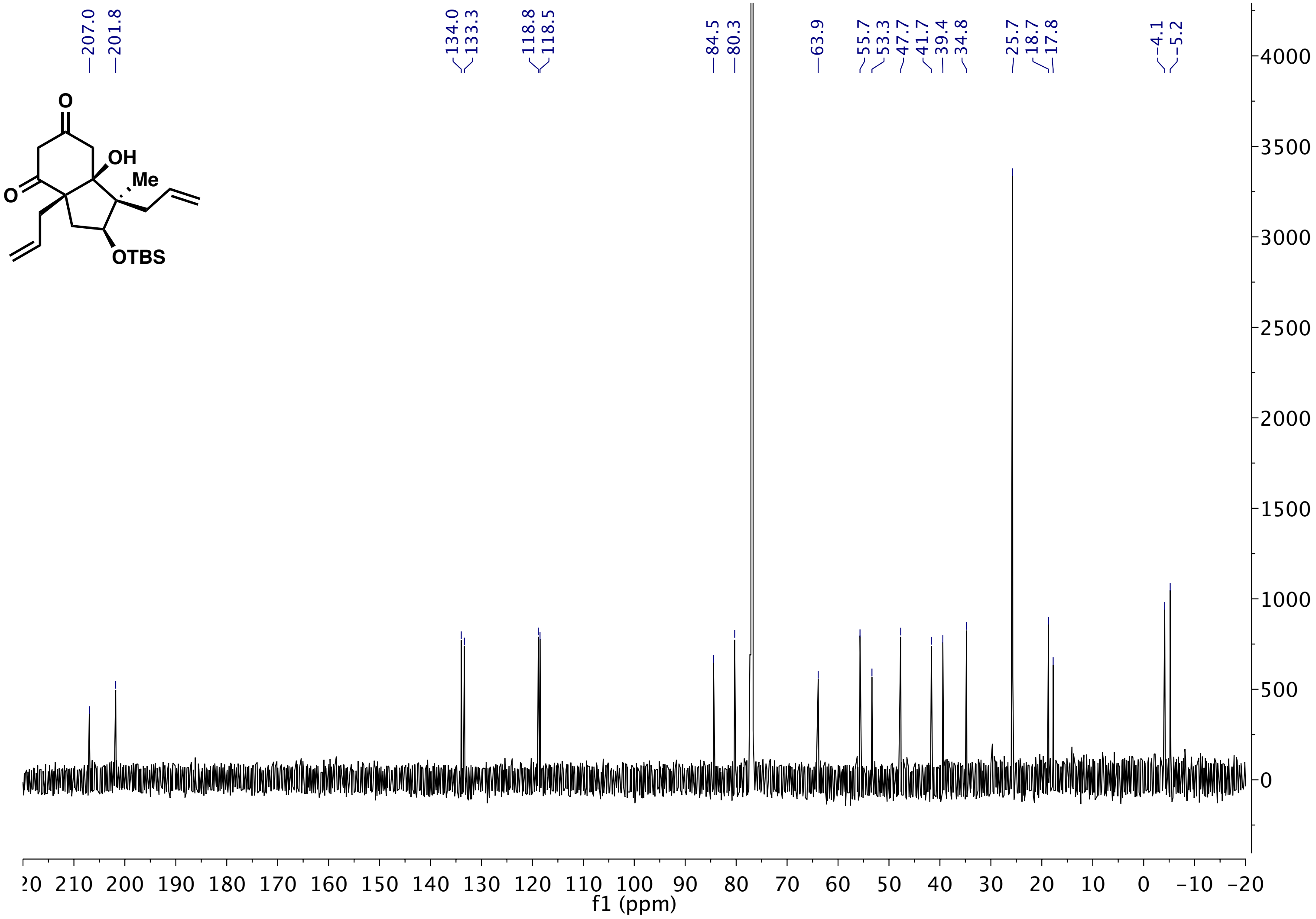

Supplementary Figure 16. ^{13}C NMR of **29**

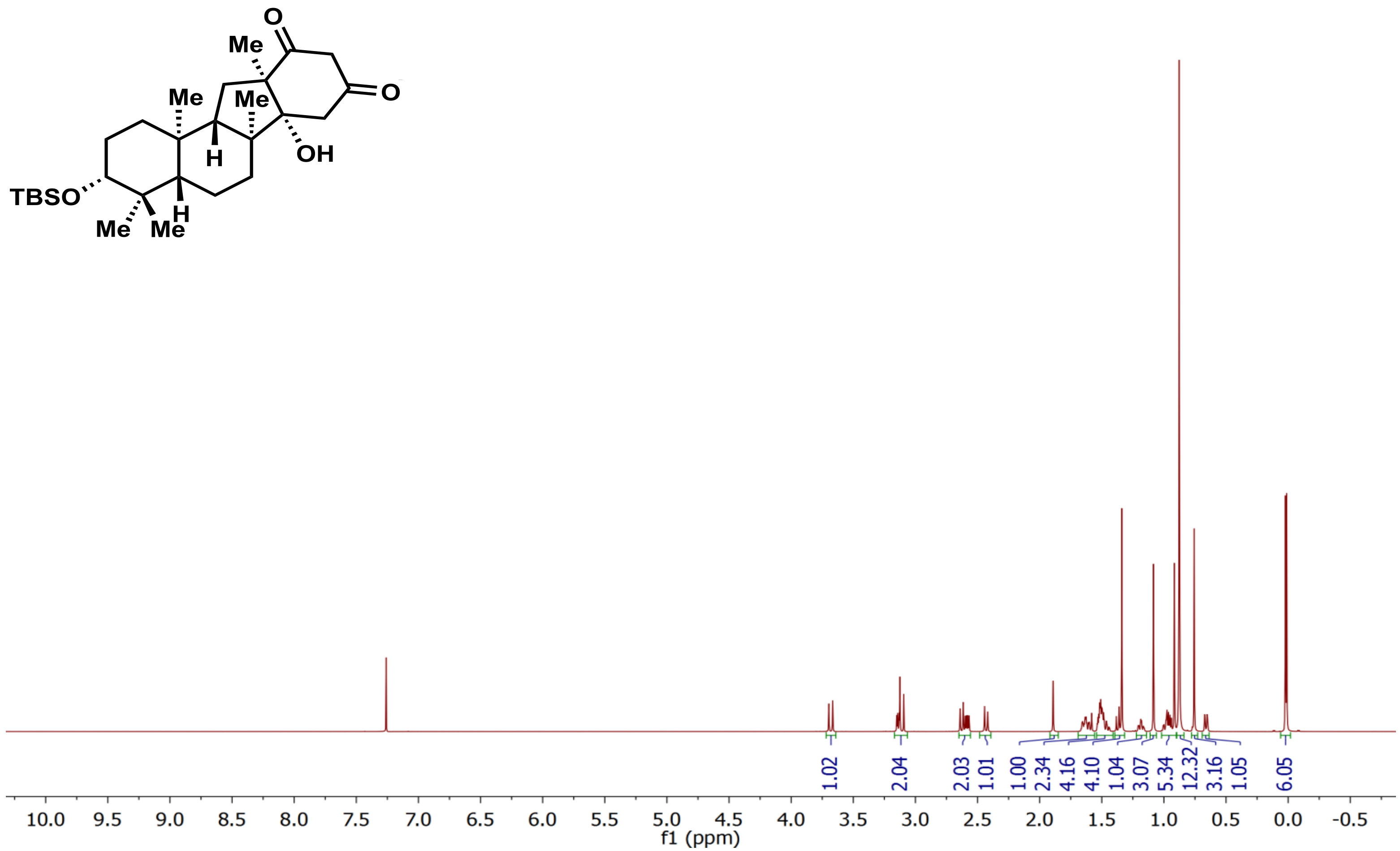

Supplementary Figure 17. ^{1}H NMR of **30**

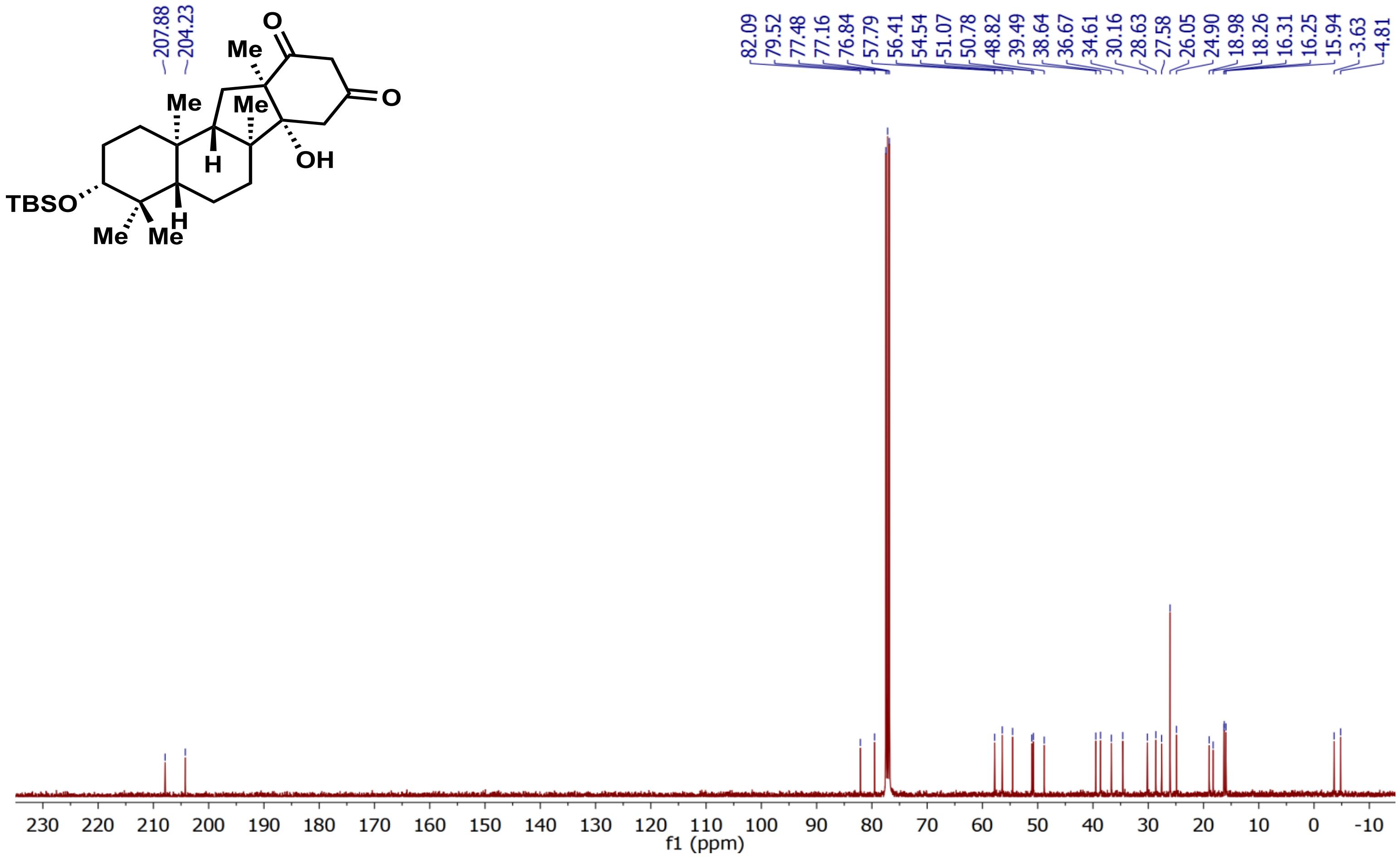

Supplementary Figure 18. ^{13}C NMR of **30**

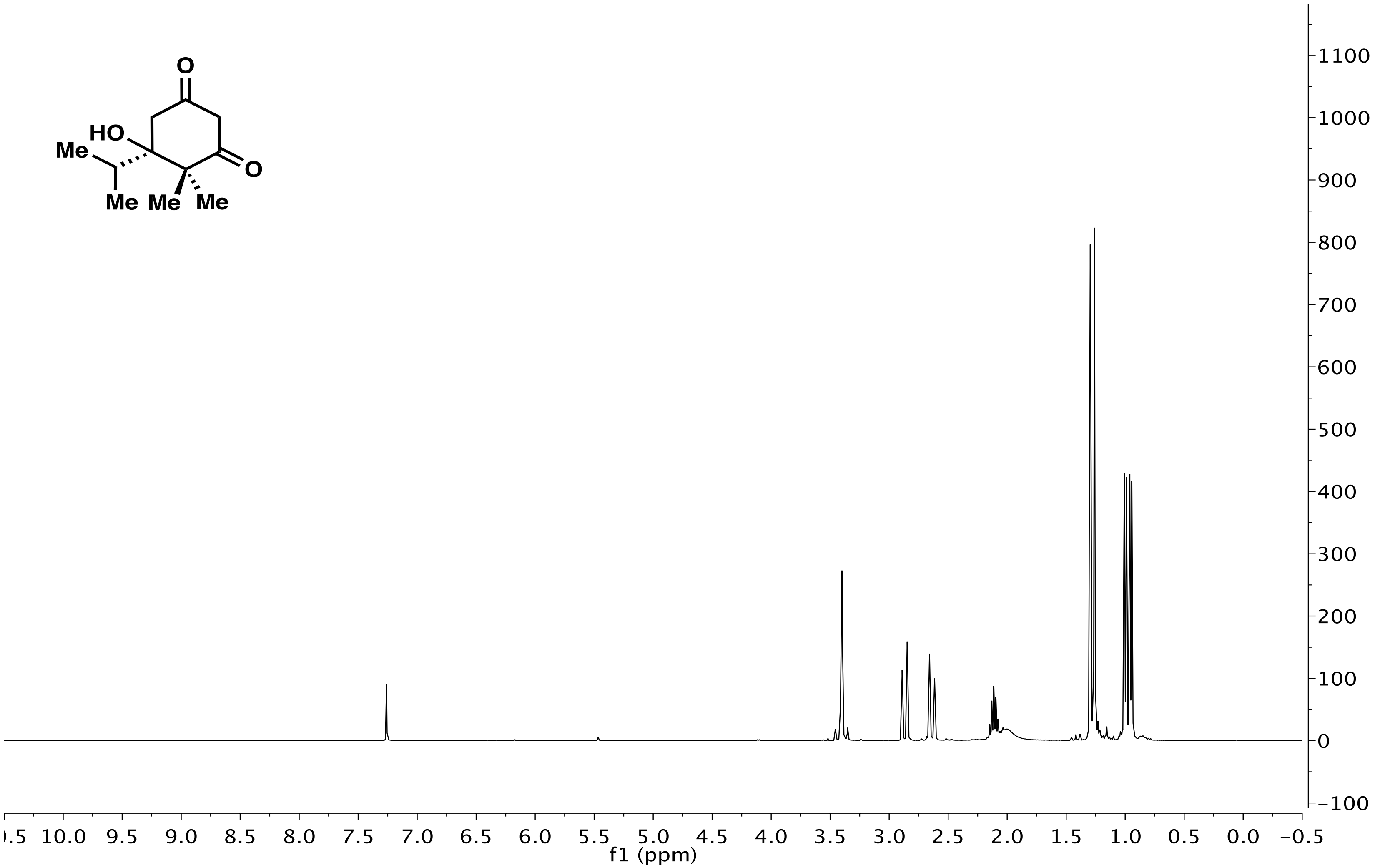

Supplementary Figure 19. ^{1}H NMR of **31**

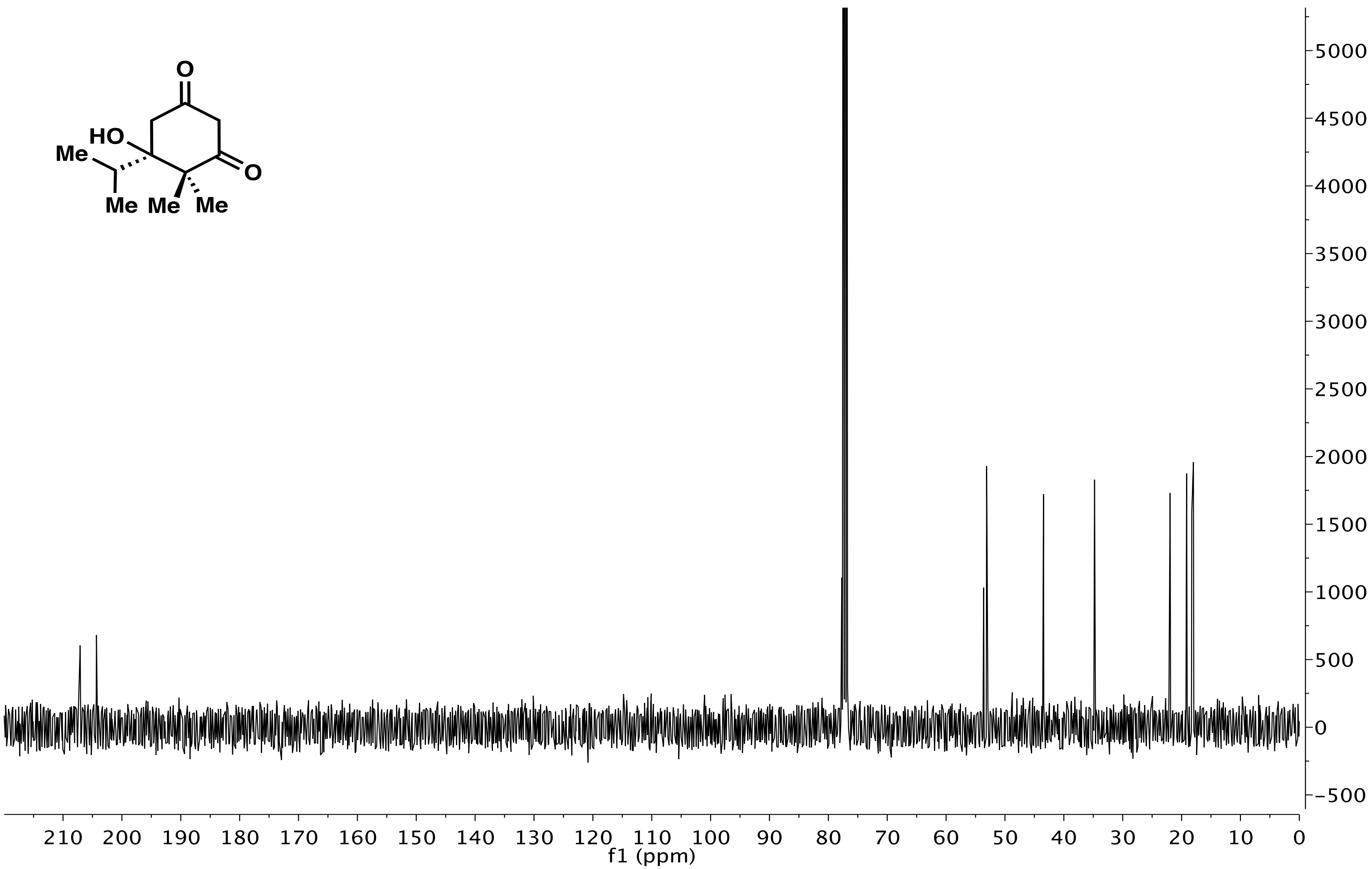

Supplementary Figure 20. ^{13}C NMR of **31**

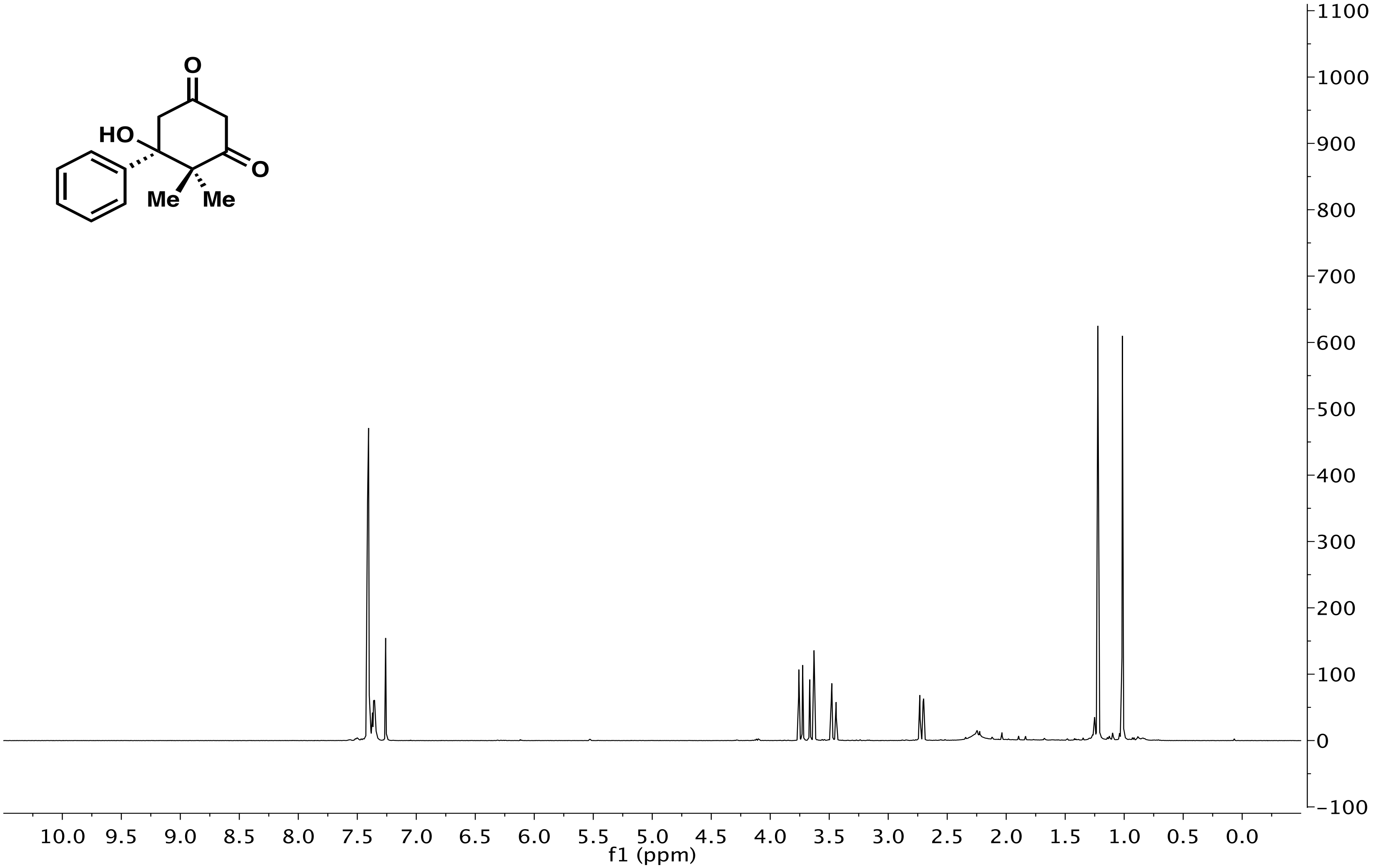

Supplementary Figure 21. ^{1}H NMR of **32**

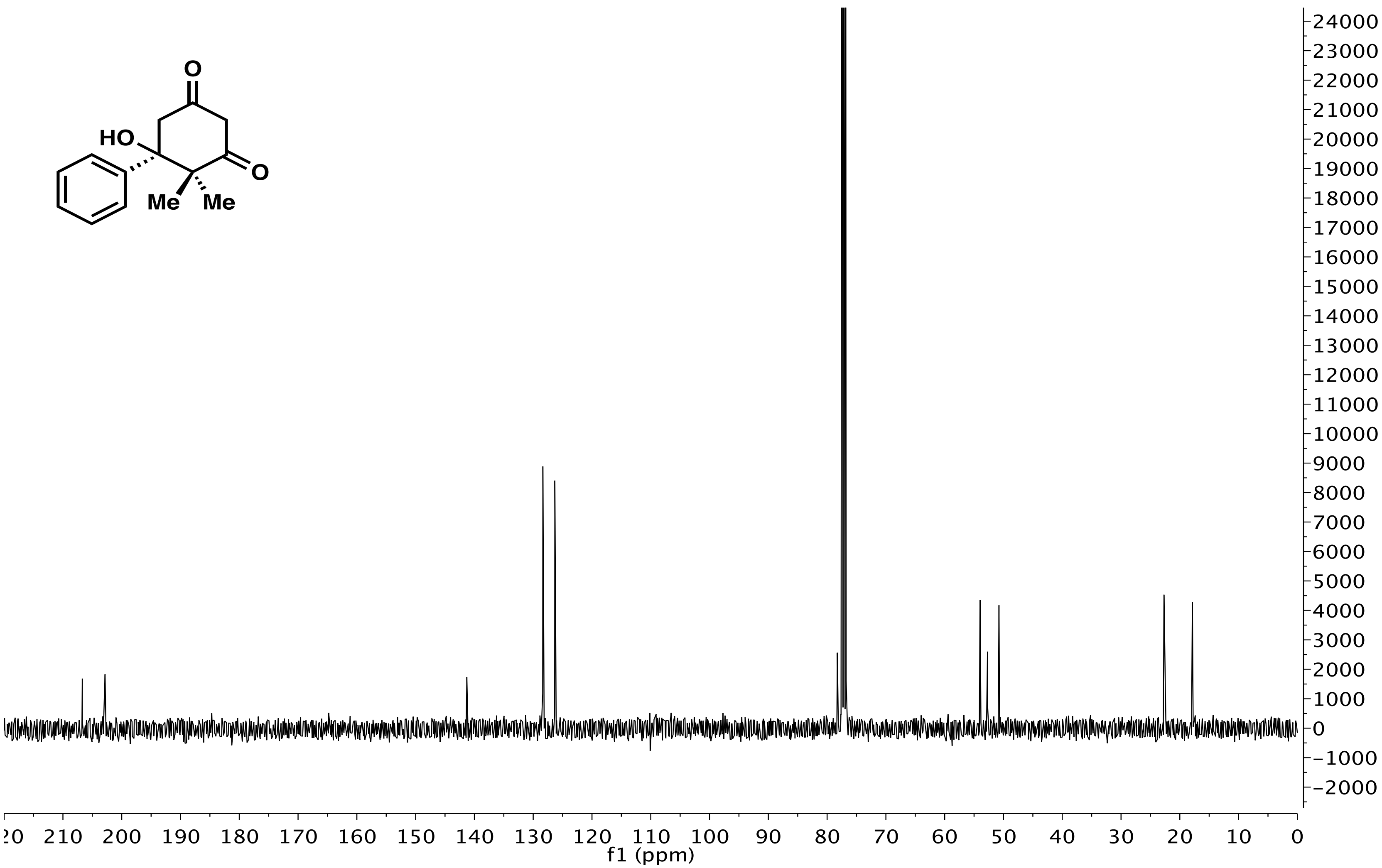

Supplementary Figure 22. ^{13}C NMR of **32**

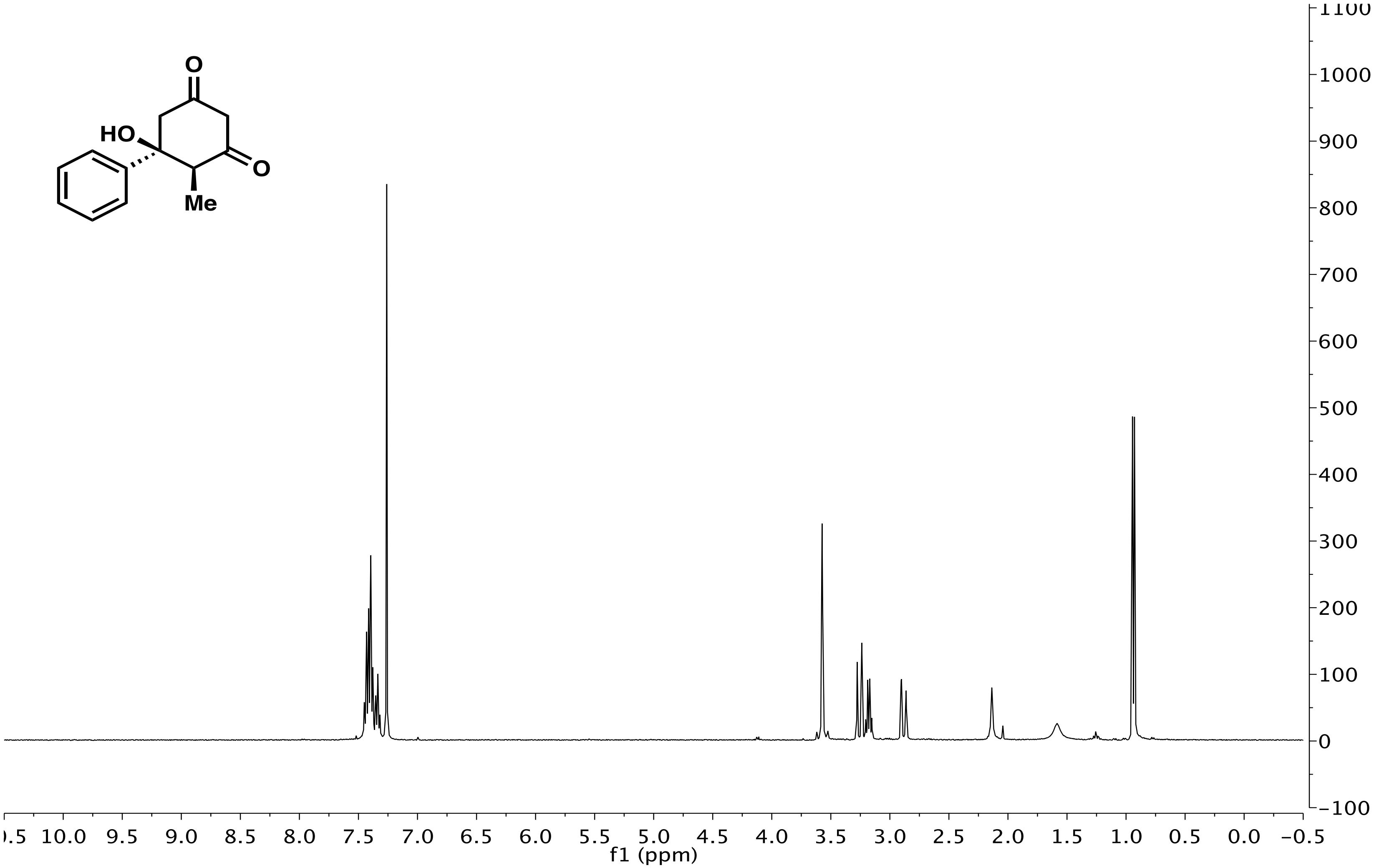

Supplementary Figure 23. ^{1}H NMR of **33**

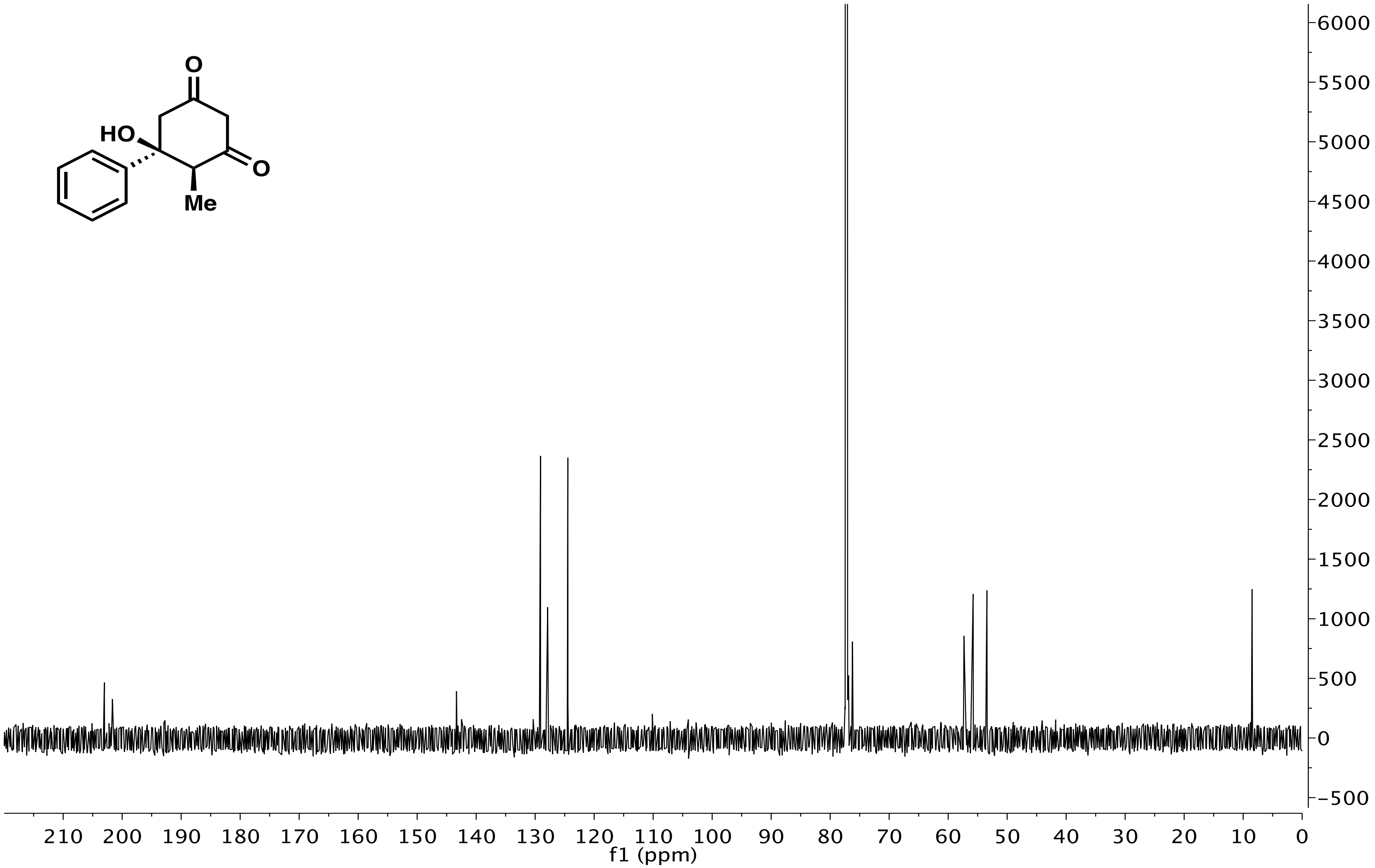

Supplementary Figure 24. ^{13}C NMR of **33**

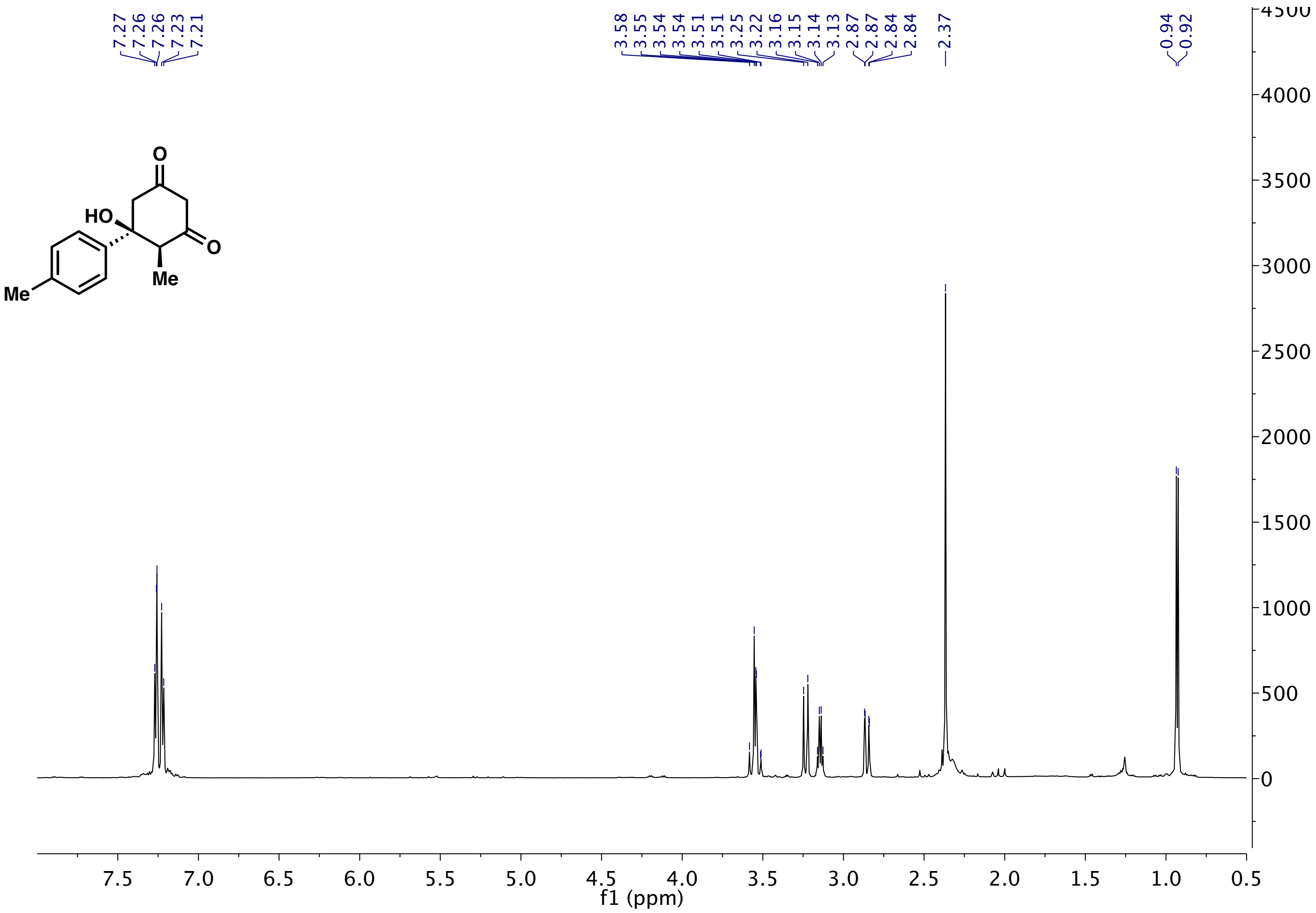

Supplementary Figure 25. ^{1}H NMR of **34**

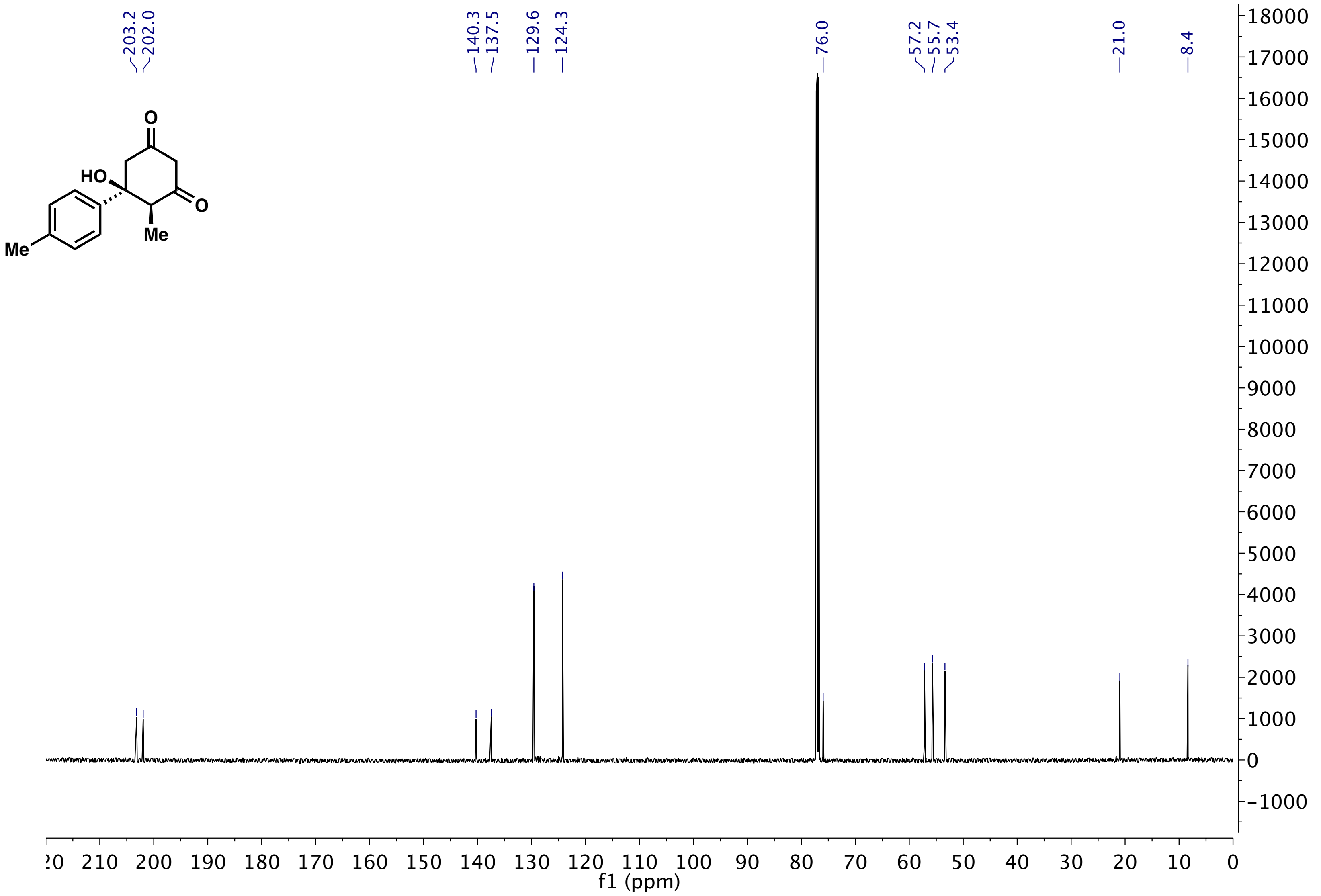

Supplementary Figure 26. ^{13}C NMR of **34**

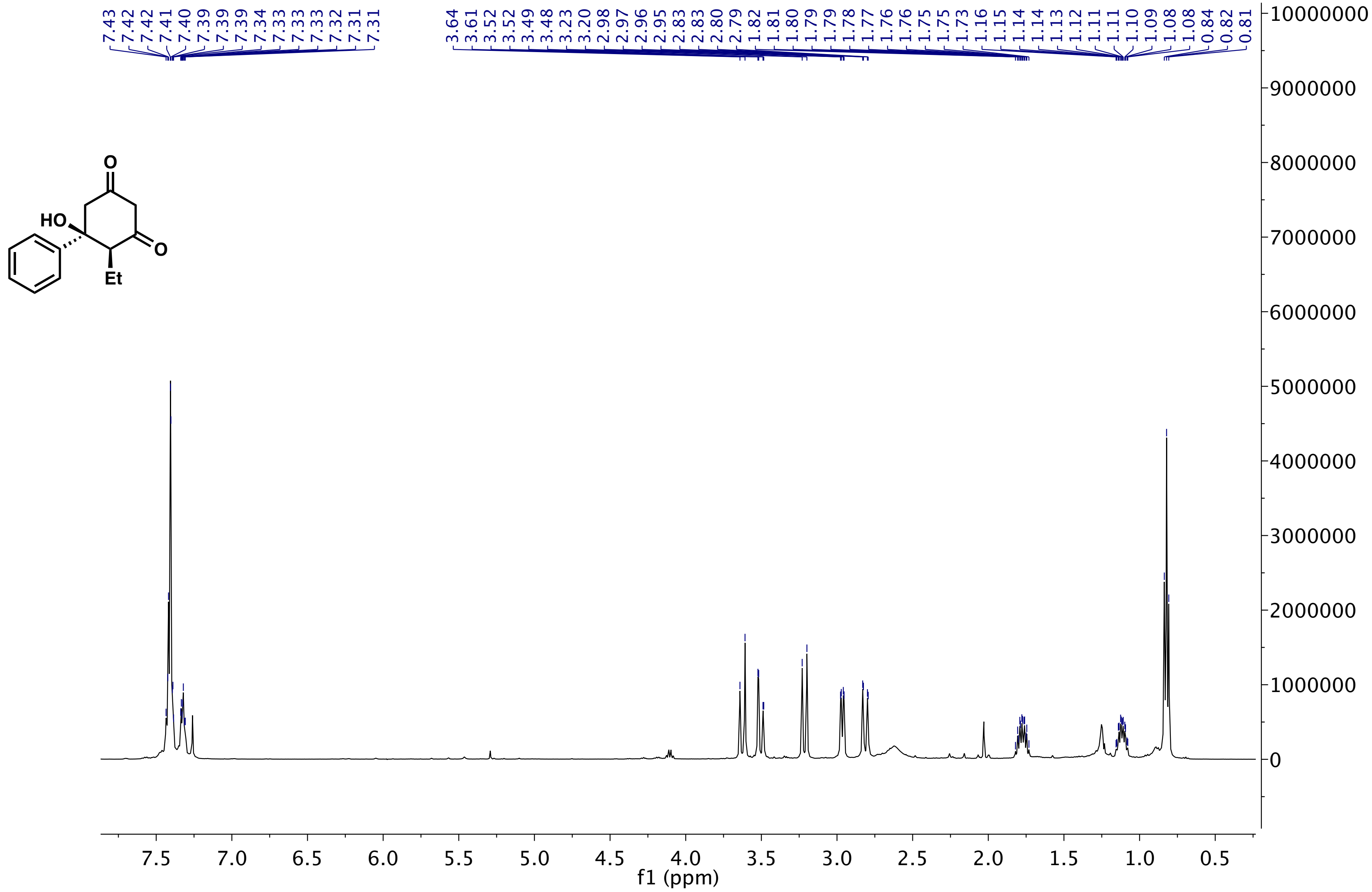

Supplementary Figure 27. ¹H NMR of **35**

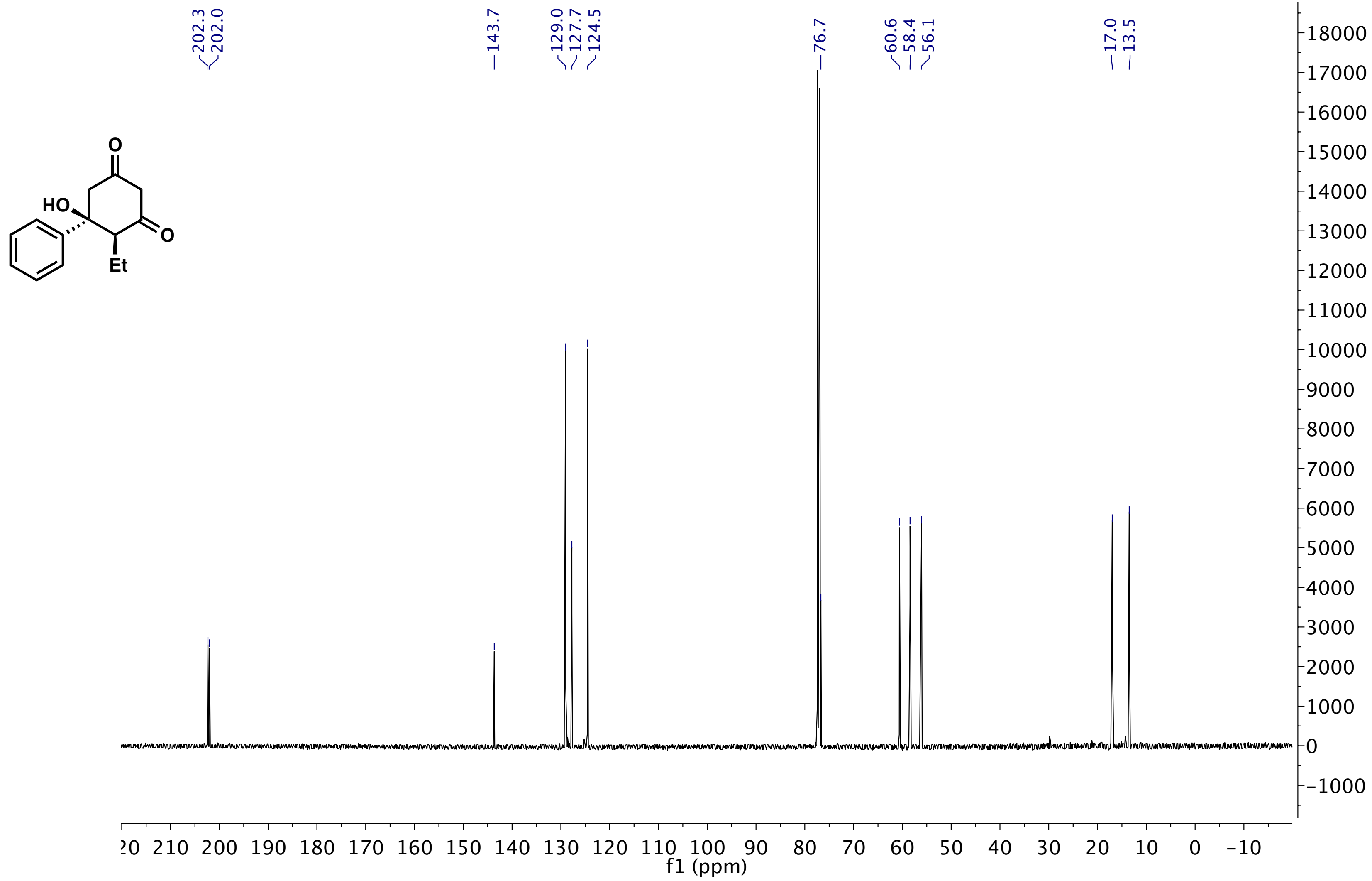

Supplementary Figure 28. ^{13}C NMR of **35**

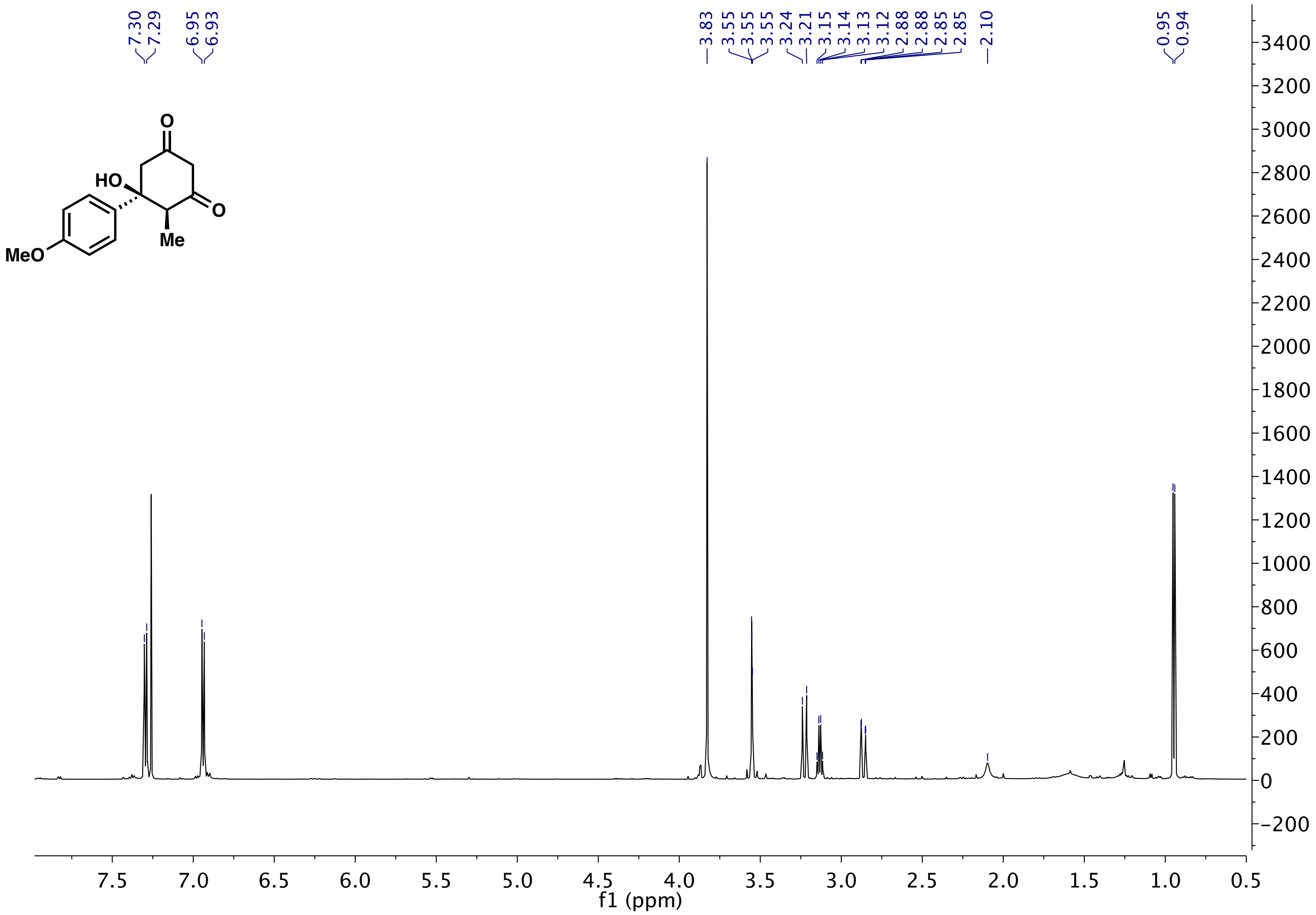

Supplementary Figure 29. ^{1}H NMR of **36**

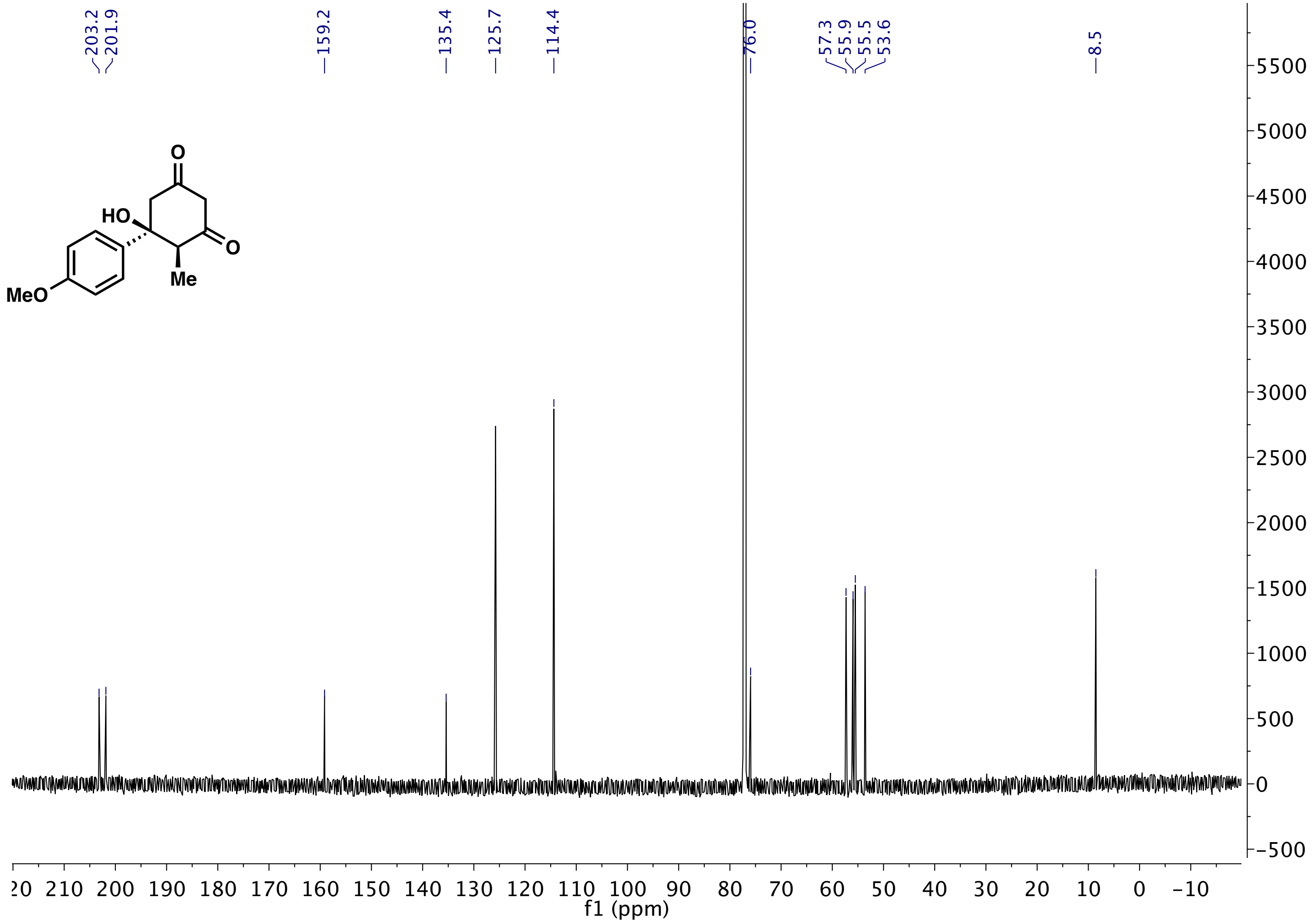

Supplementary Figure 30. ^{13}C NMR of **36**

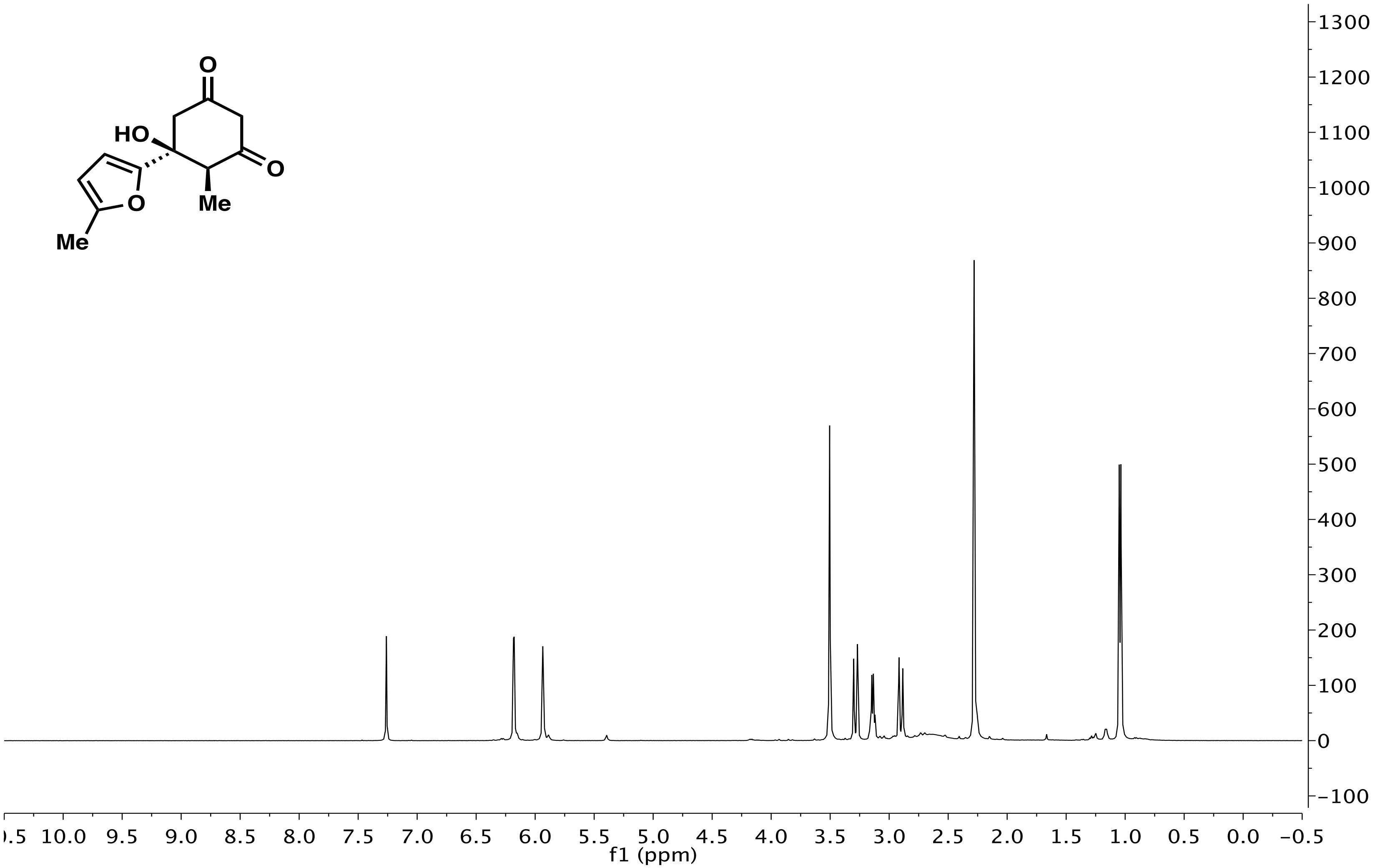

Supplementary Figure 31. ^{1}H NMR of **37**

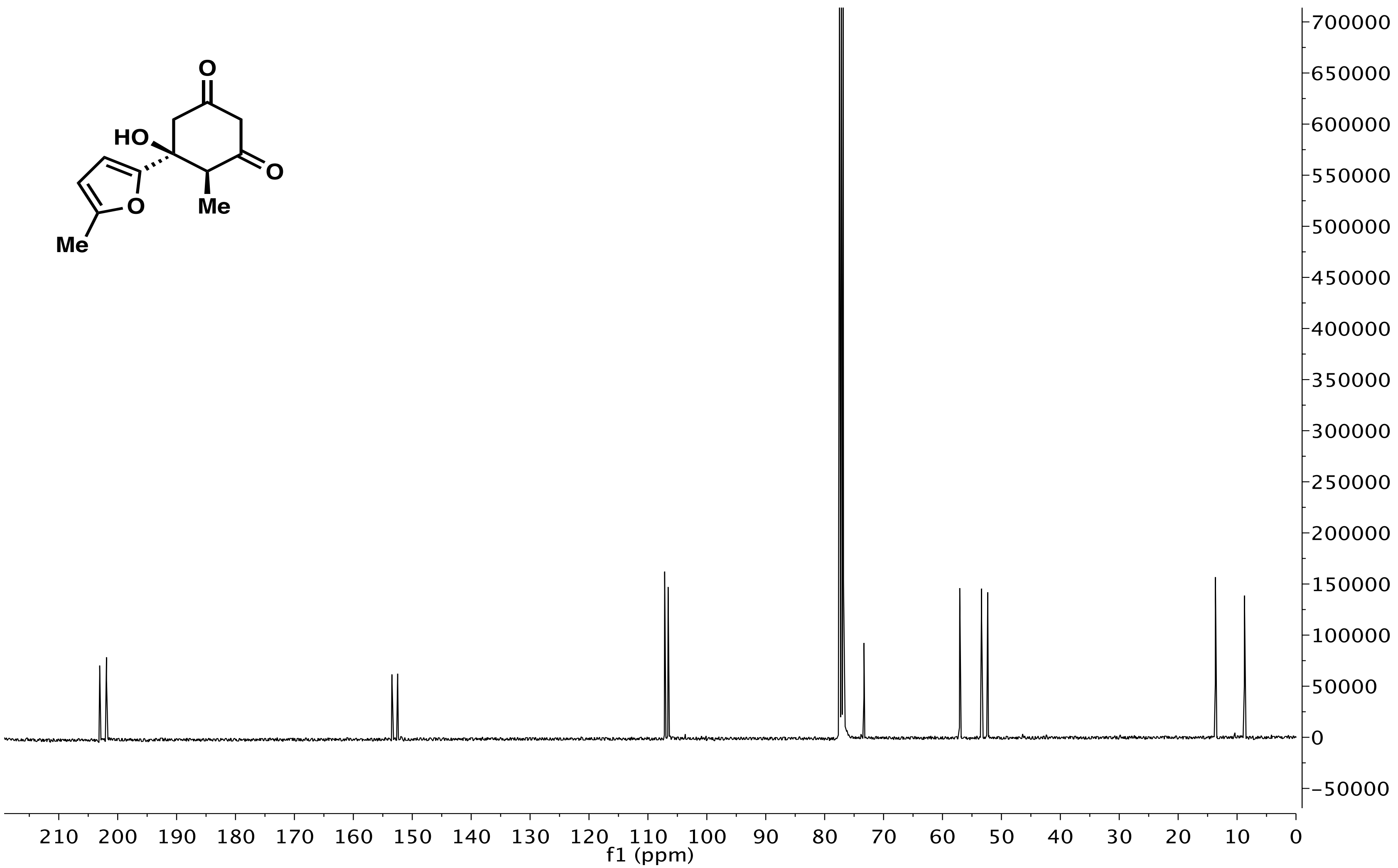

Supplementary Figure 32. ^{13}C NMR of **37**

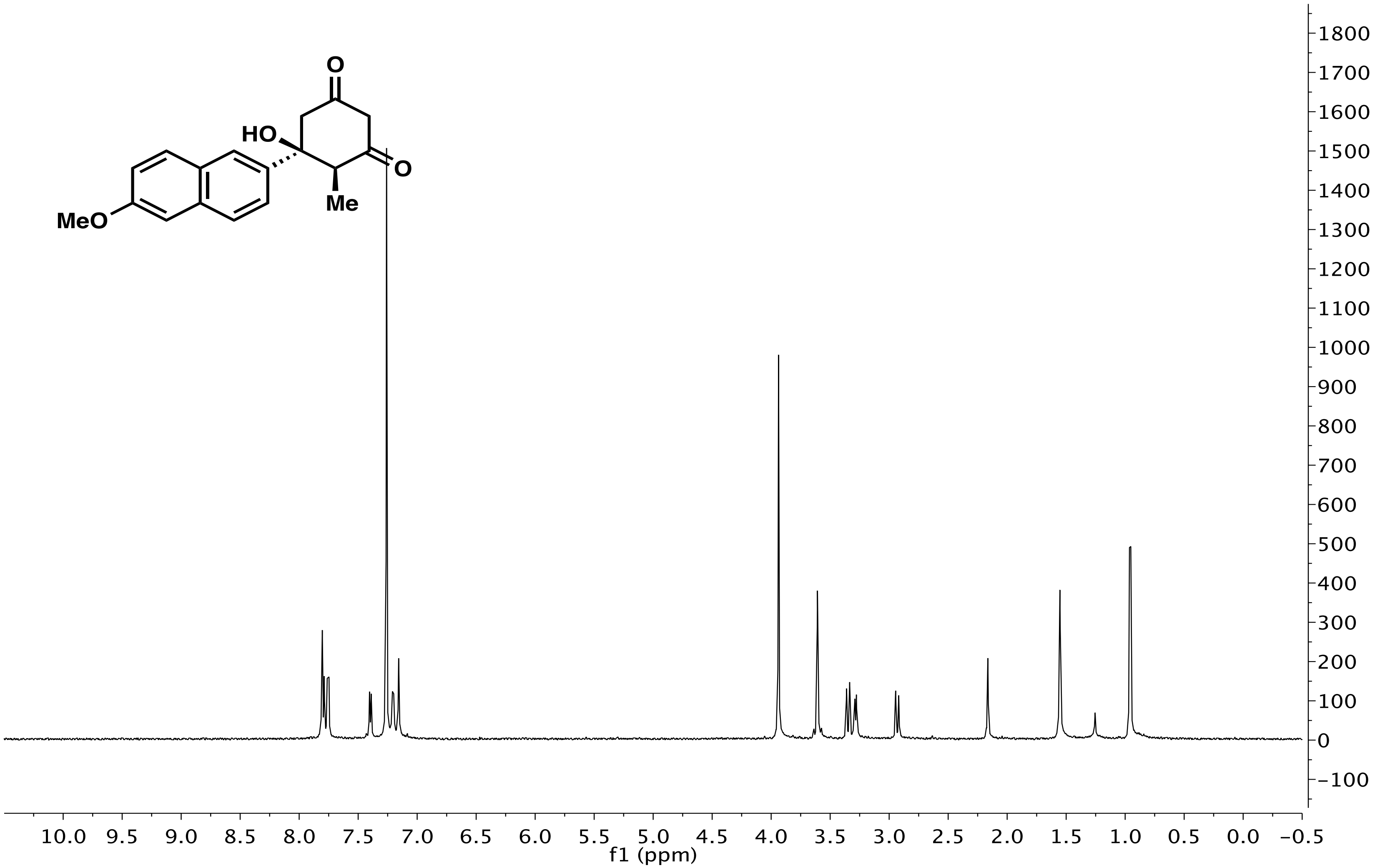

Supplementary Figure 33. ^{1}H NMR of **38**

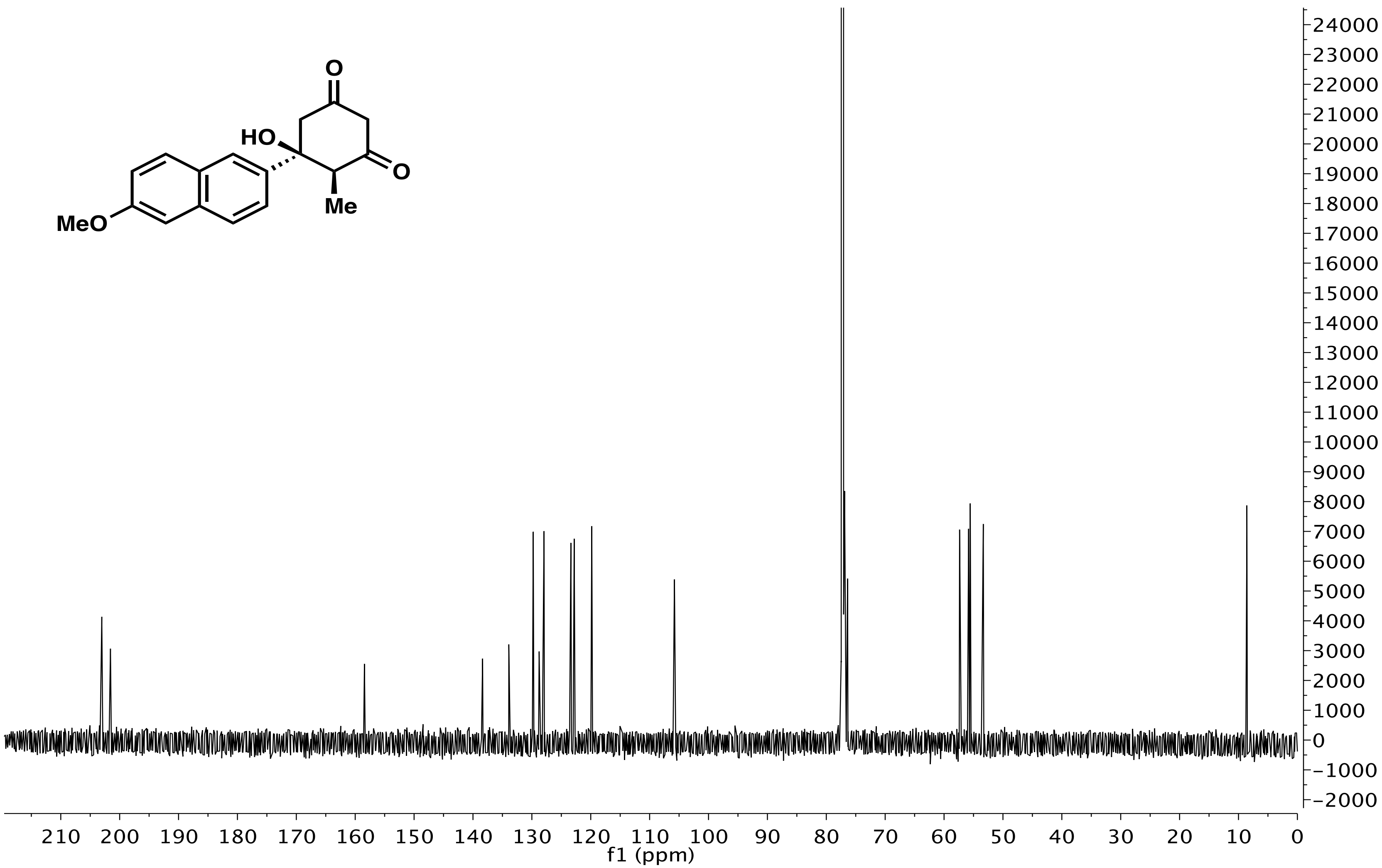

Supplementary Figure 34. ^{13}C NMR of **38**

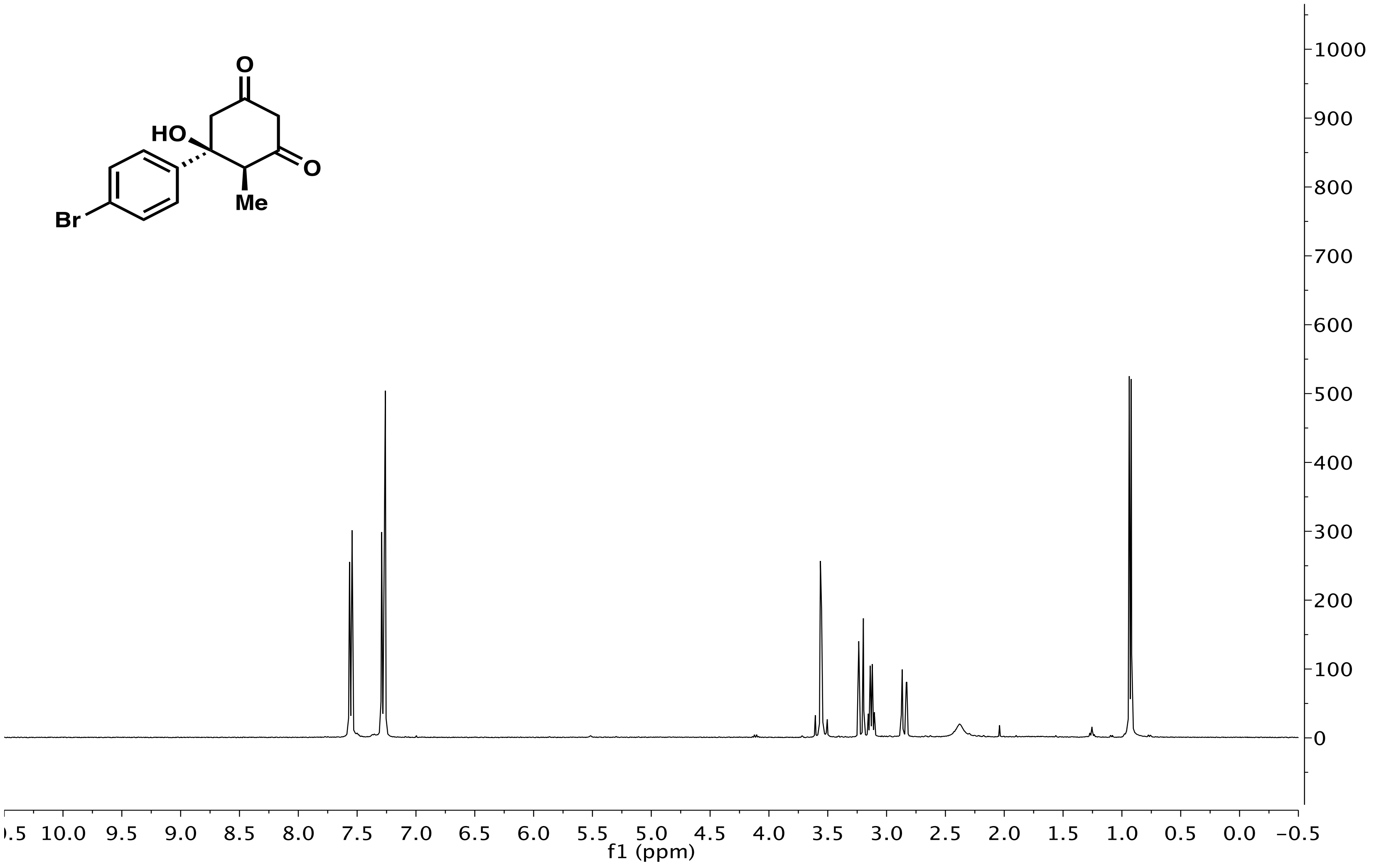

Supplementary Figure 35. ^{1}H NMR of **39**

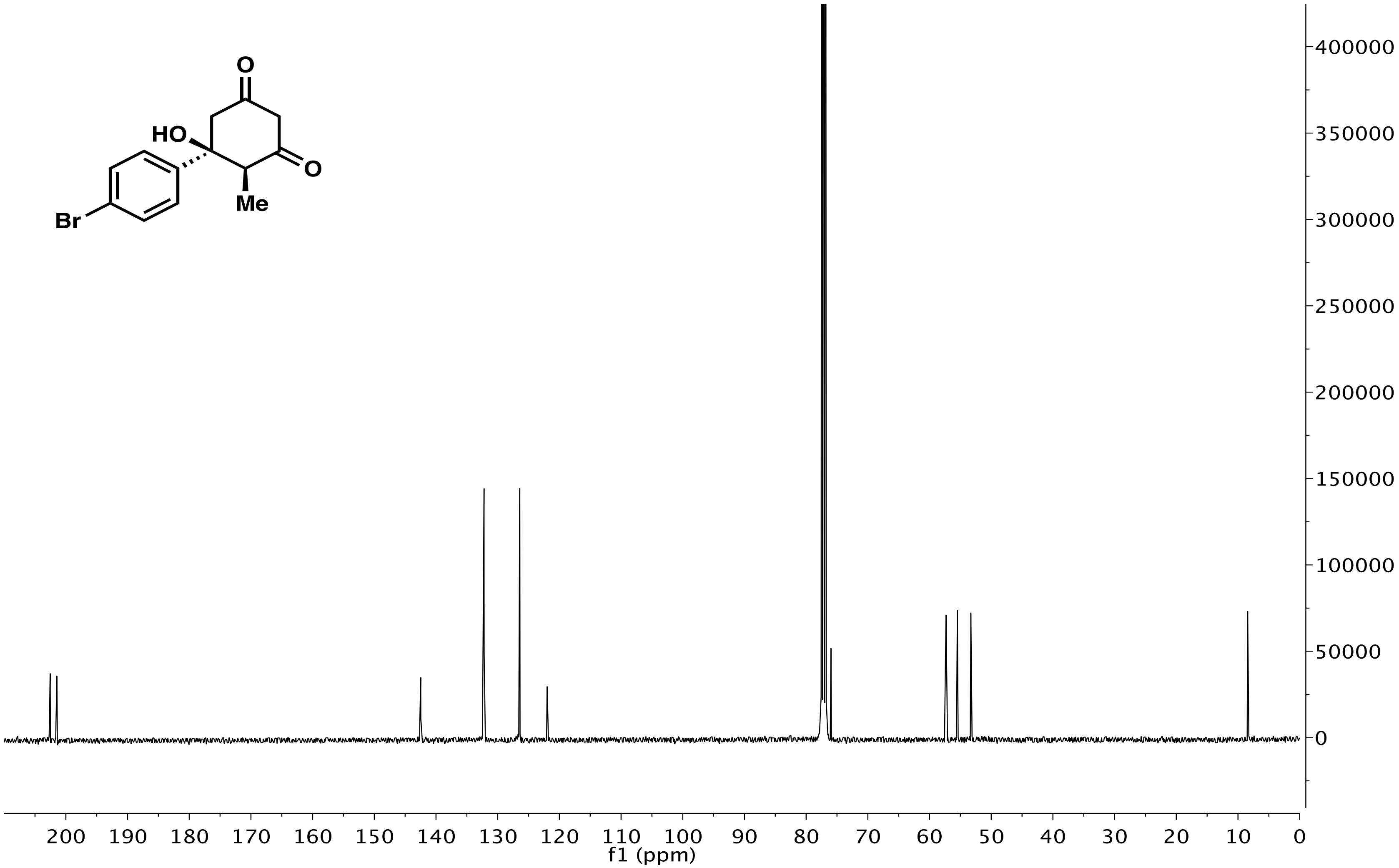

Supplementary Figure 36. ^{13}C NMR of **39**

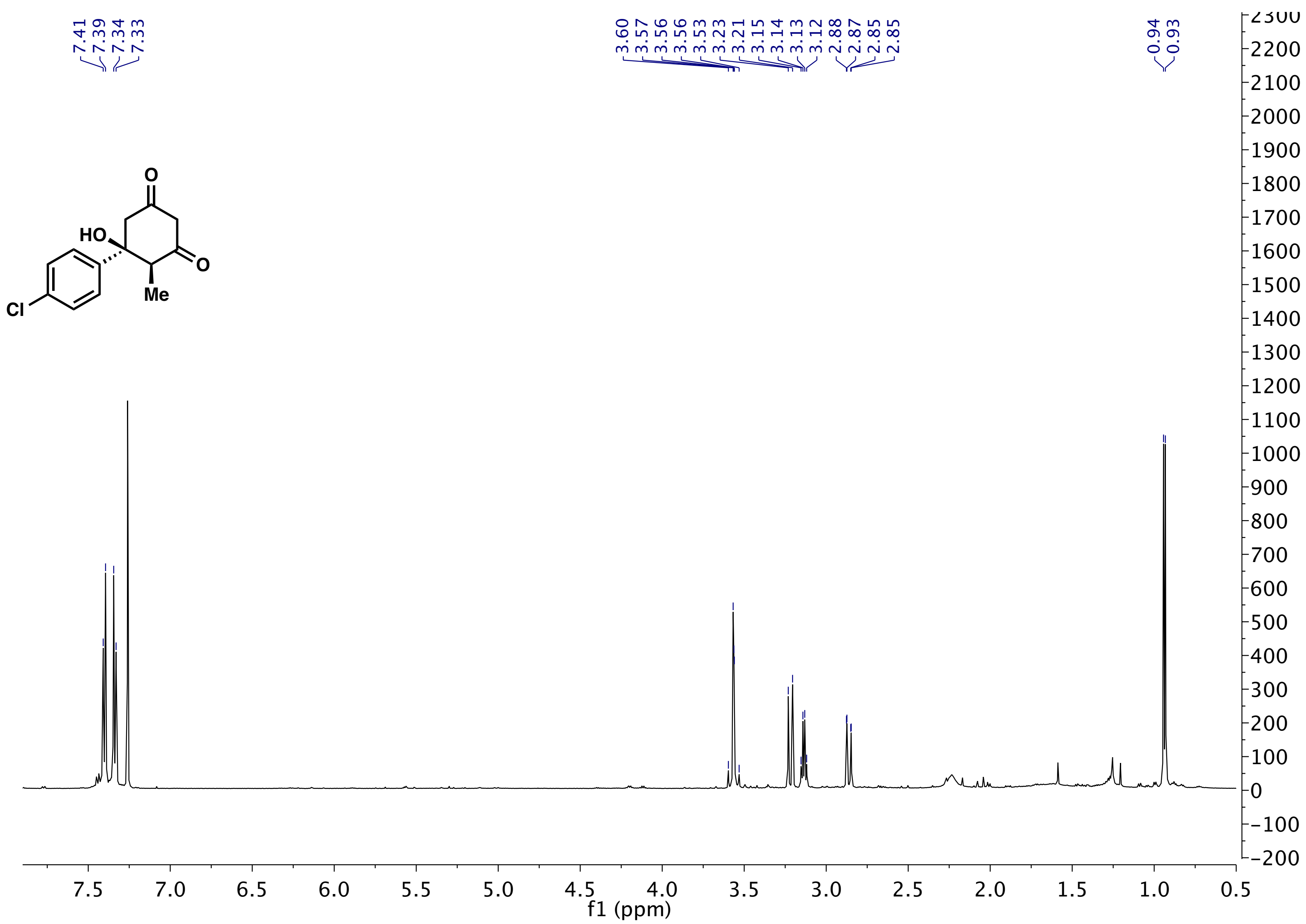

Supplementary Figure 37. ^{1}H NMR of **40**

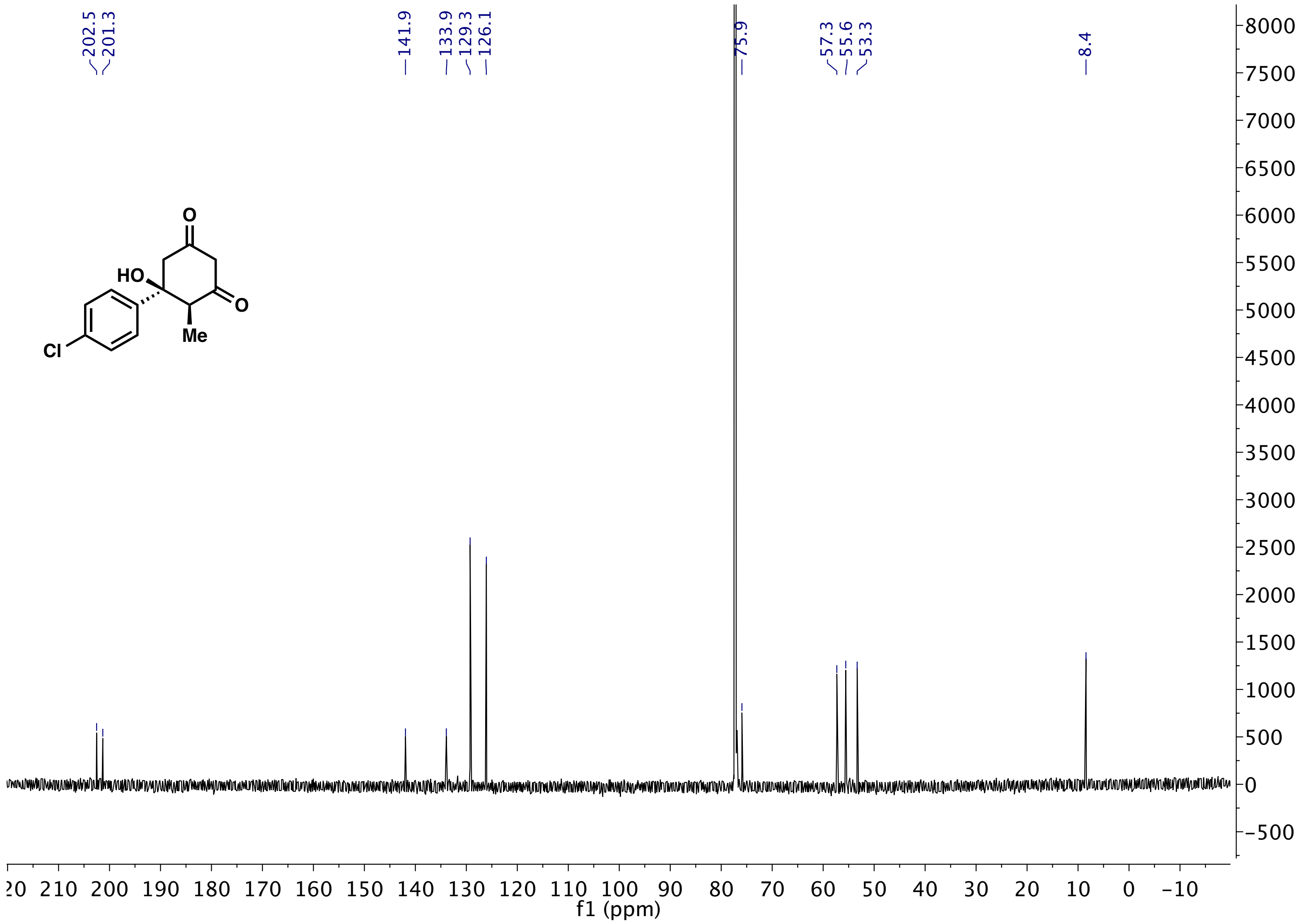

Supplementary Figure 38. ^{13}C NMR of **40**

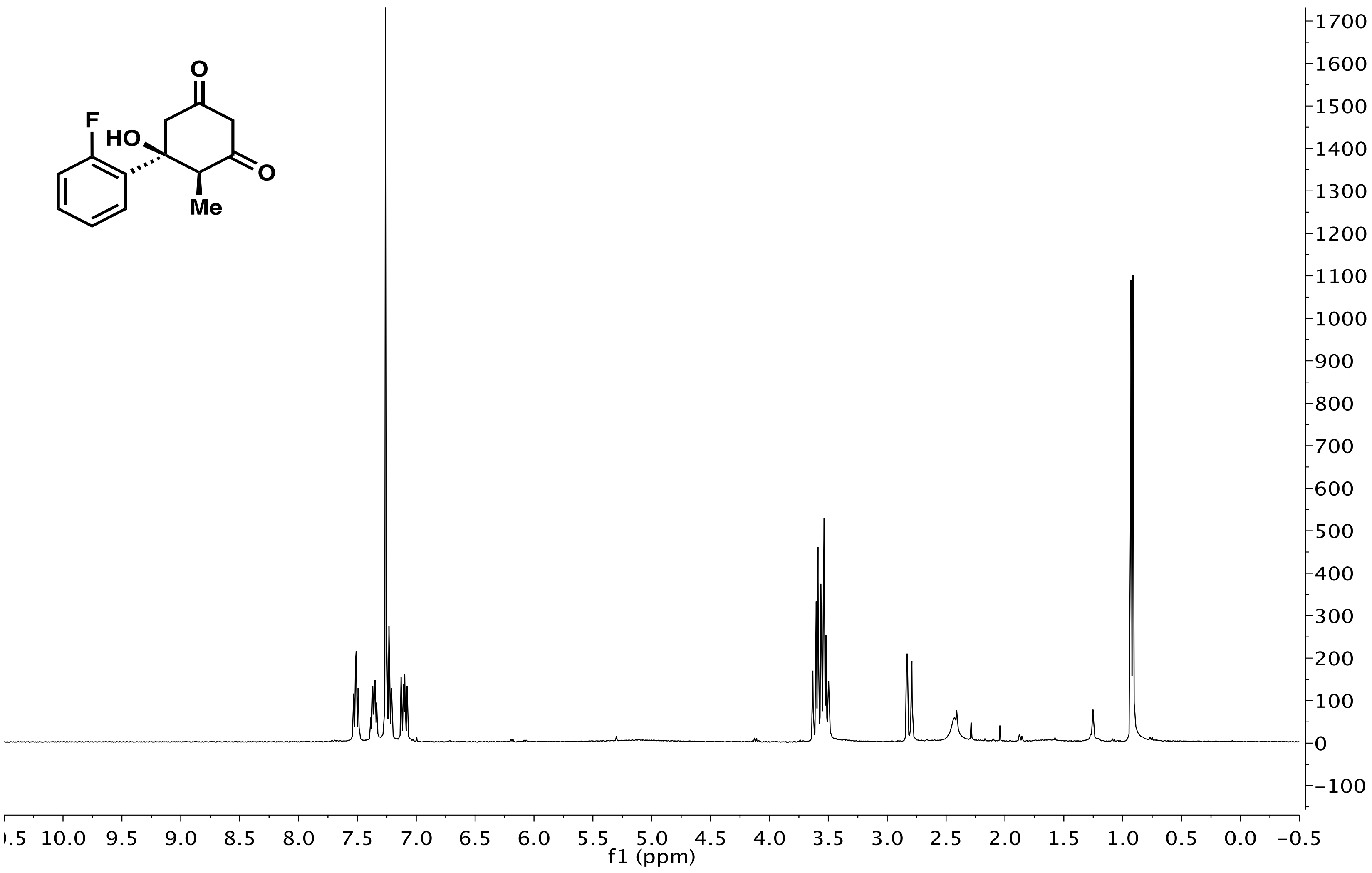

Supplementary Figure 39. ^{1}H NMR of **41**

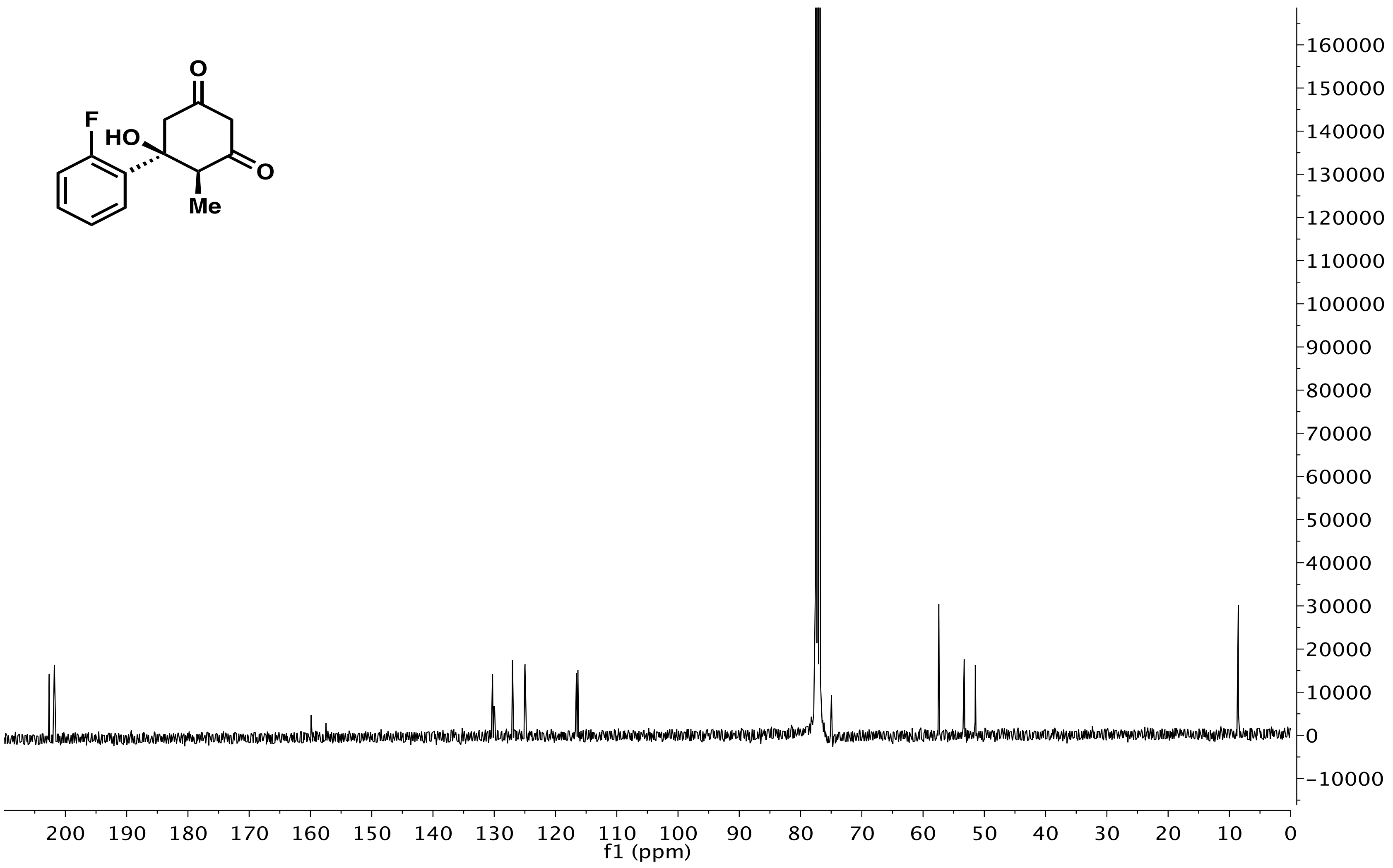

Supplementary Figure 40. ^{13}C NMR of **41**

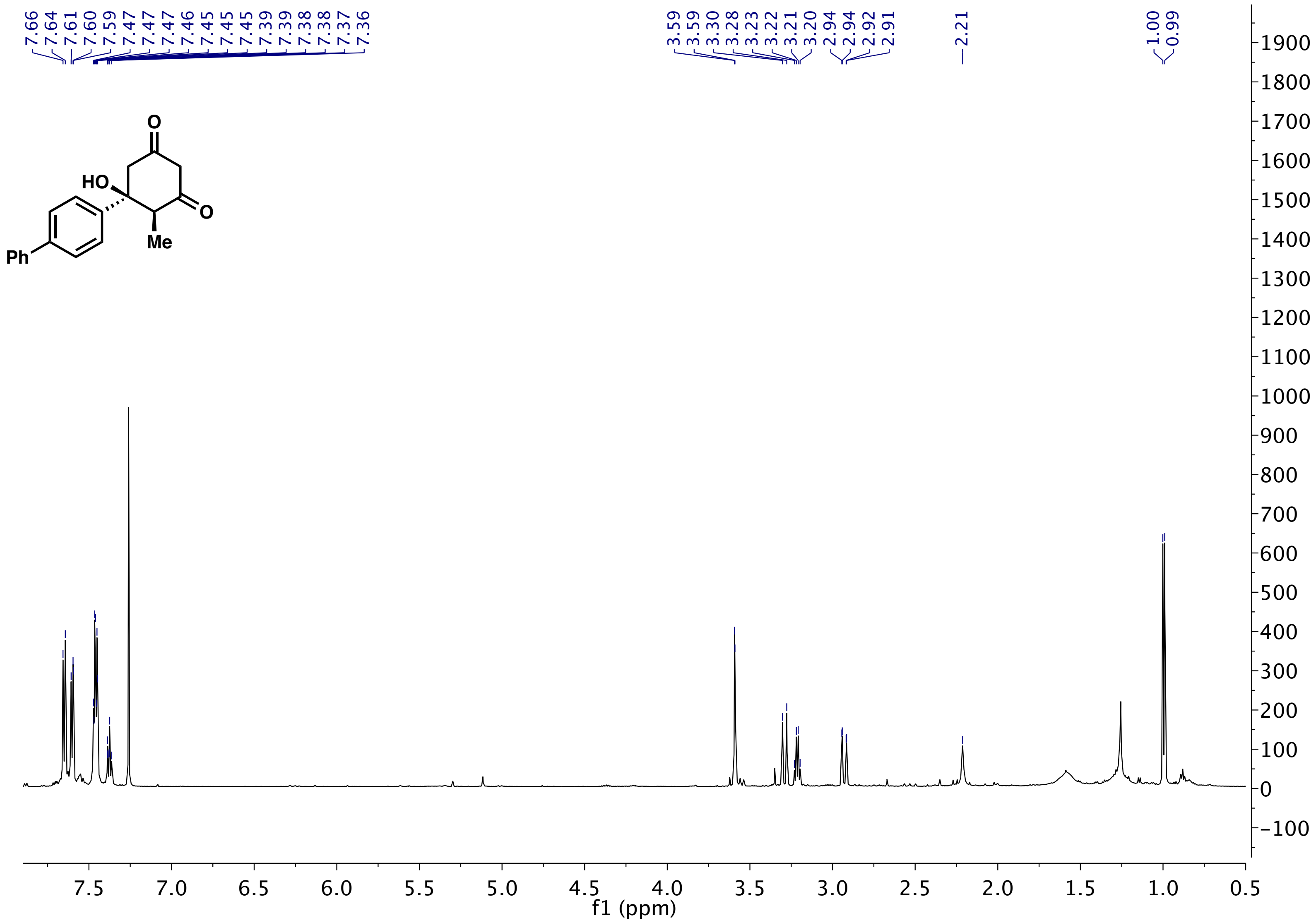

Supplementary Figure 41. ^{1}H NMR of **42**

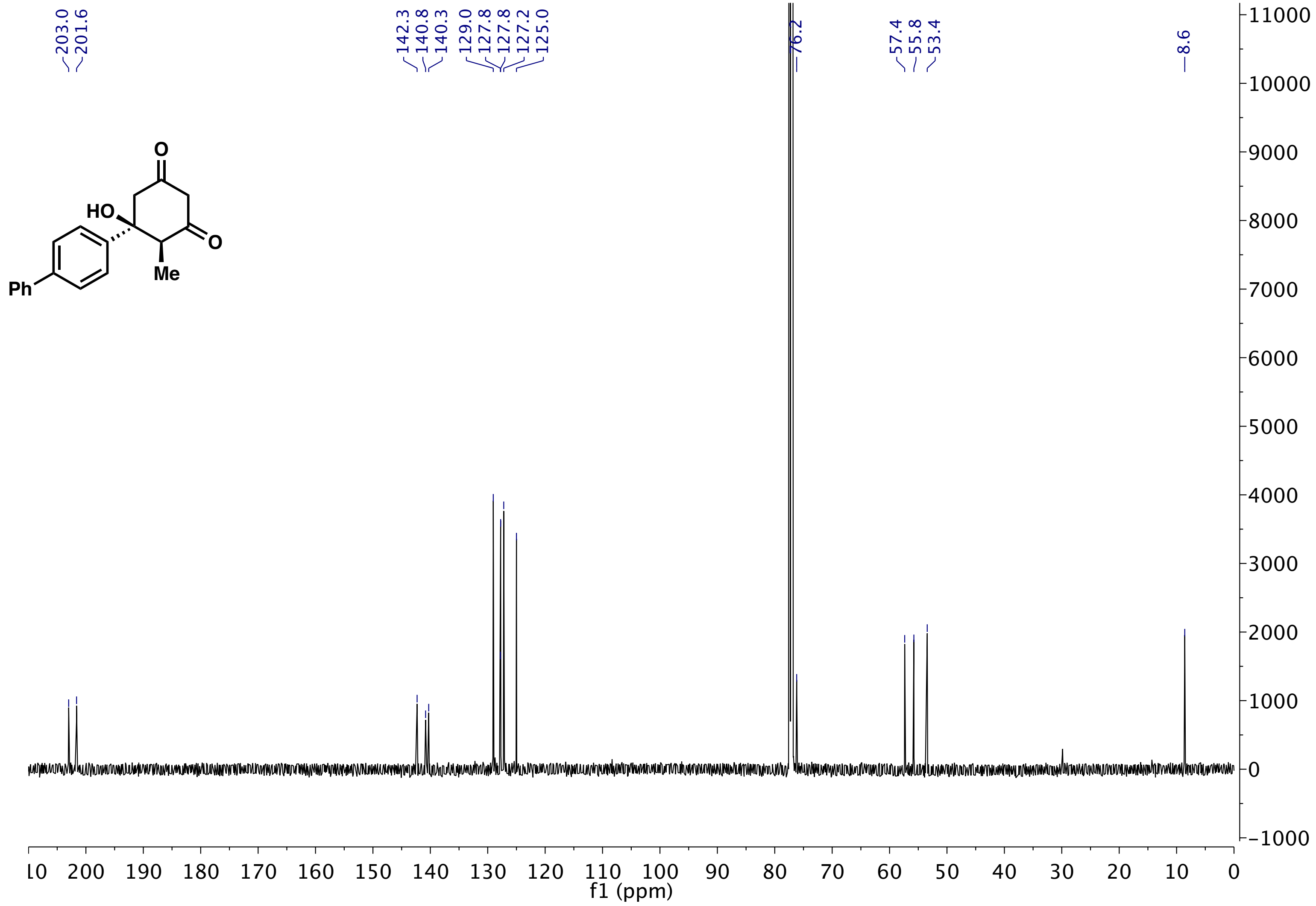

Supplementary Figure 42. ^{13}C NMR of **42**

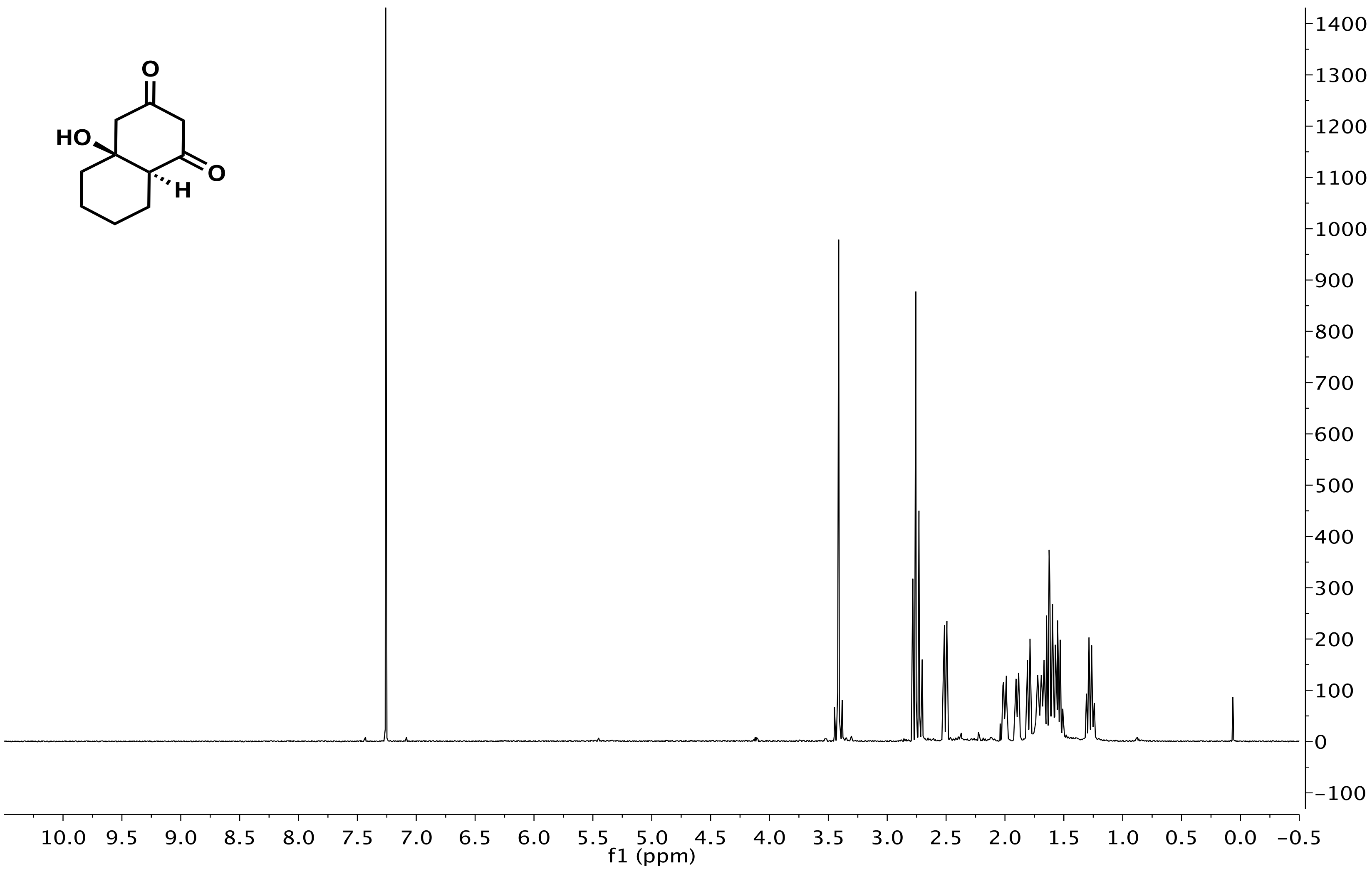

Supplementary Figure 43. ^{1}H NMR of **43**

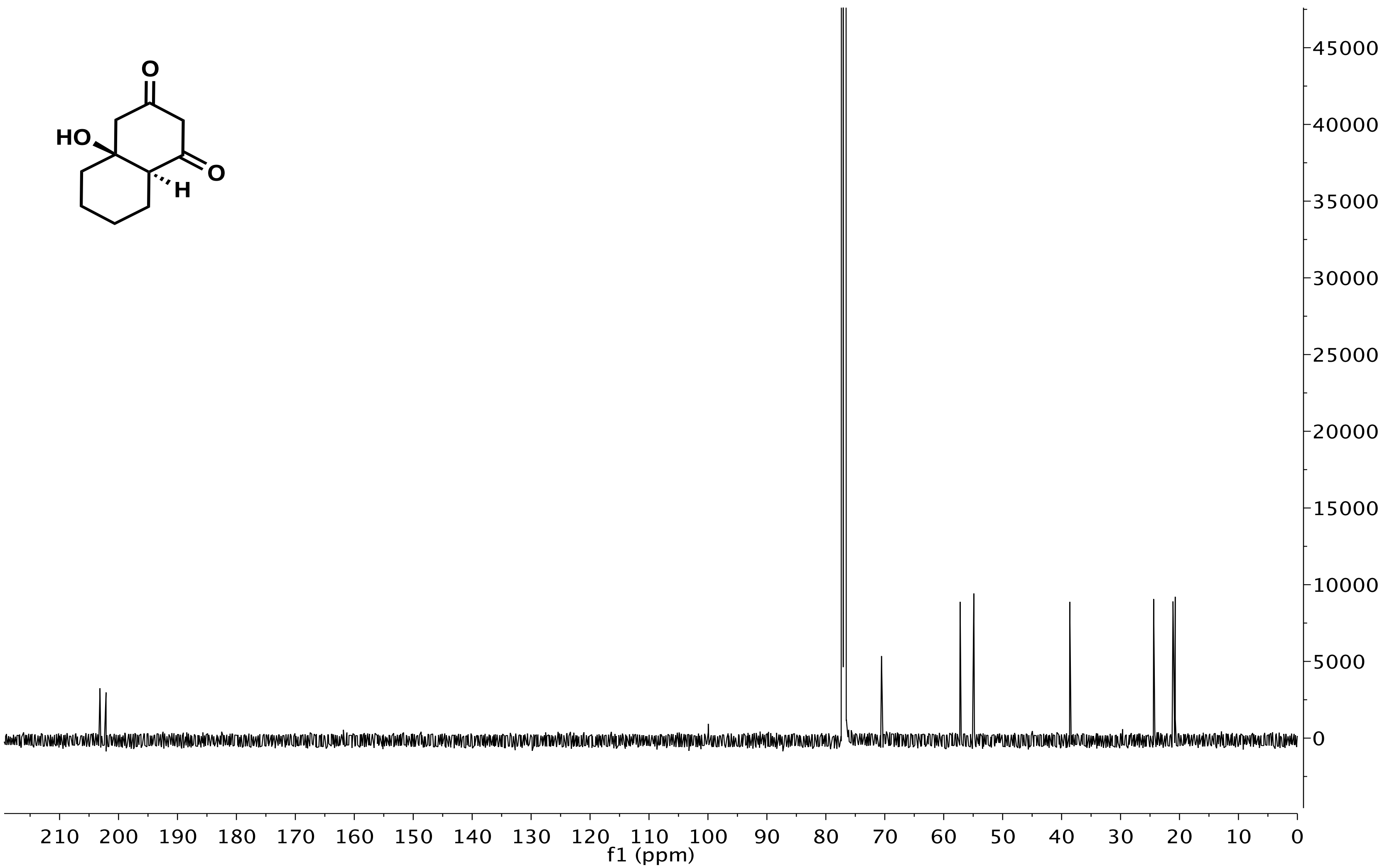

Supplementary Figure 44. ^{13}C NMR of **43**

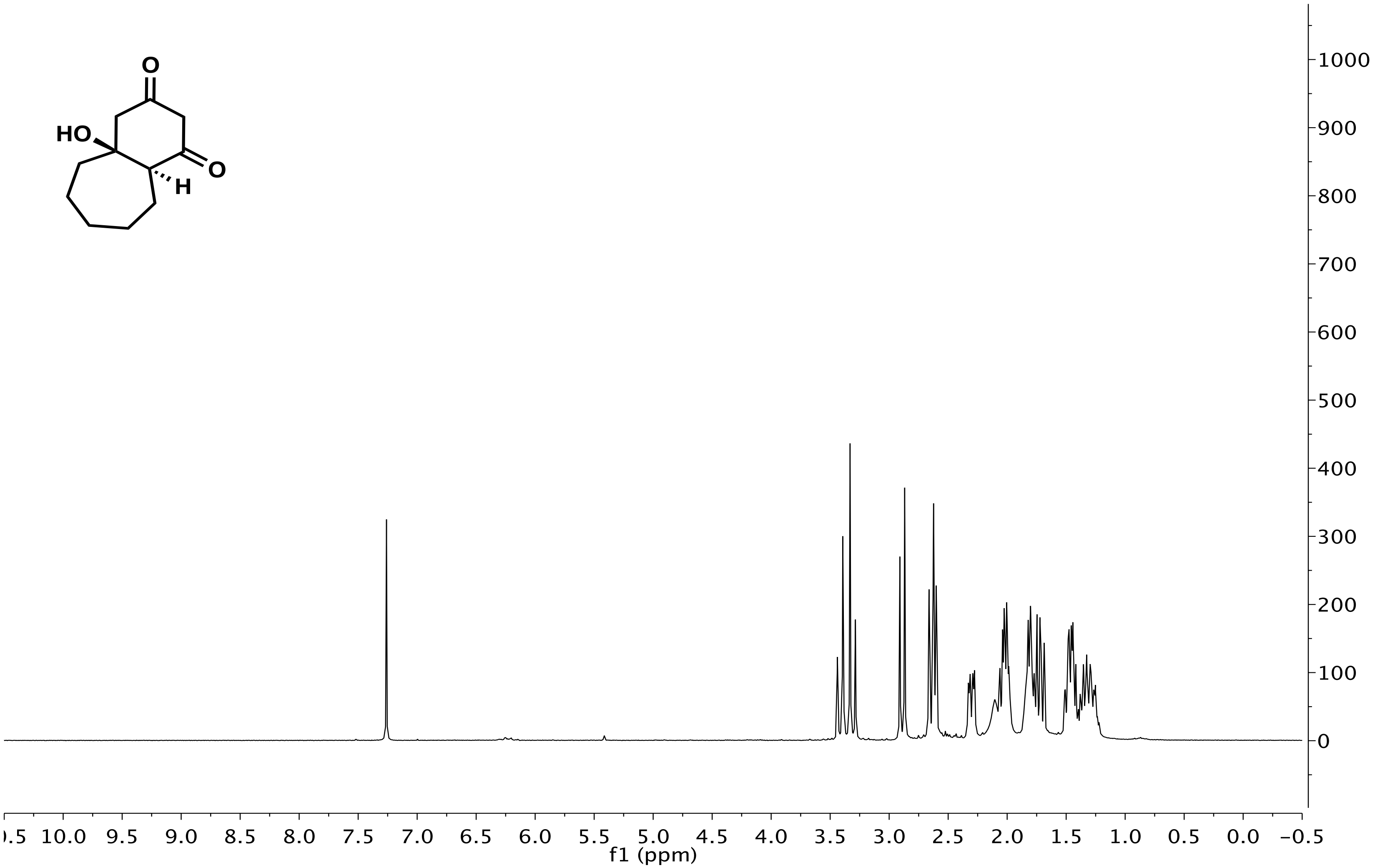

Supplementary Figure 45. ^{1}H NMR of **44**

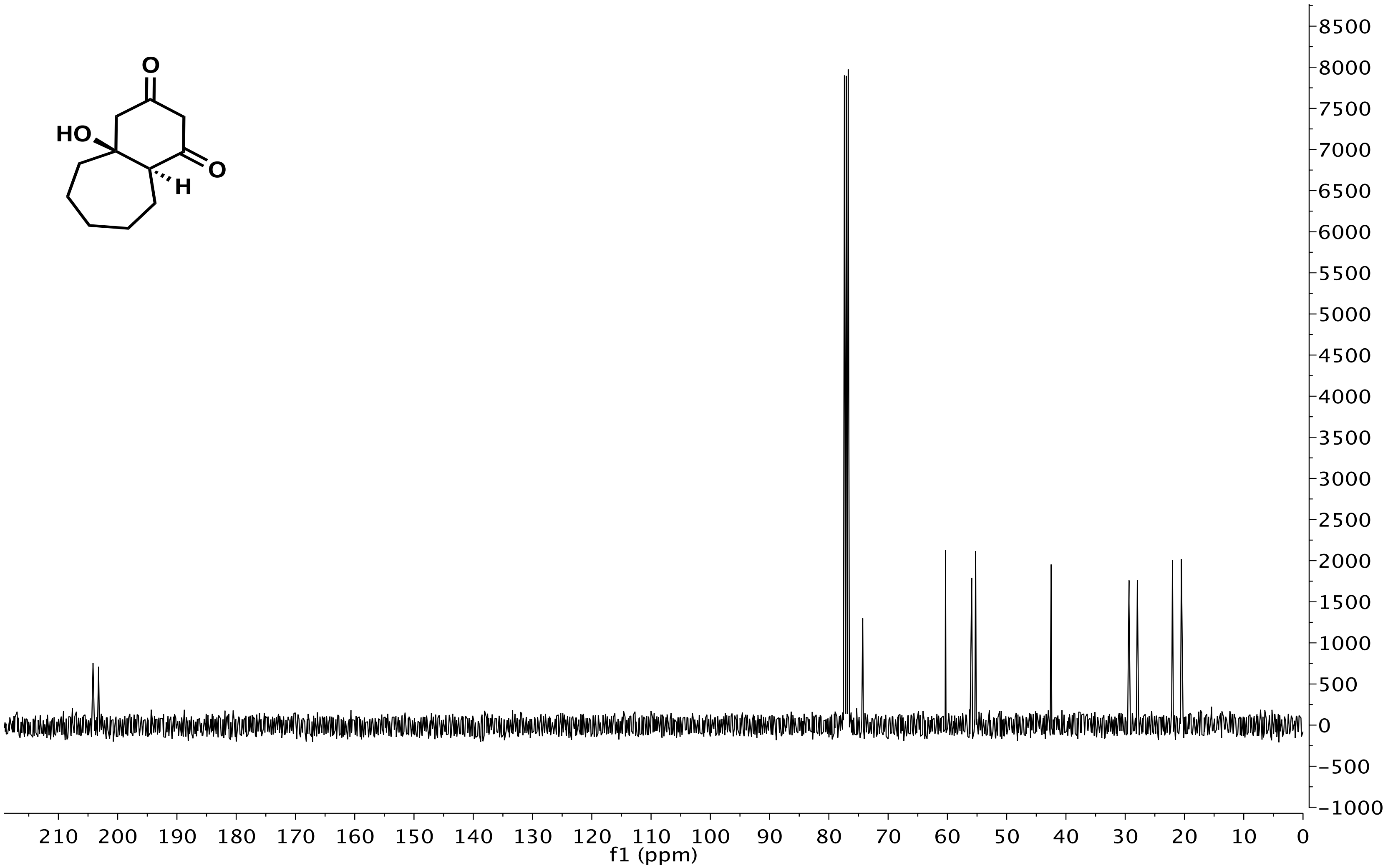

Supplementary Figure 46. ^{13}C NMR of **44**

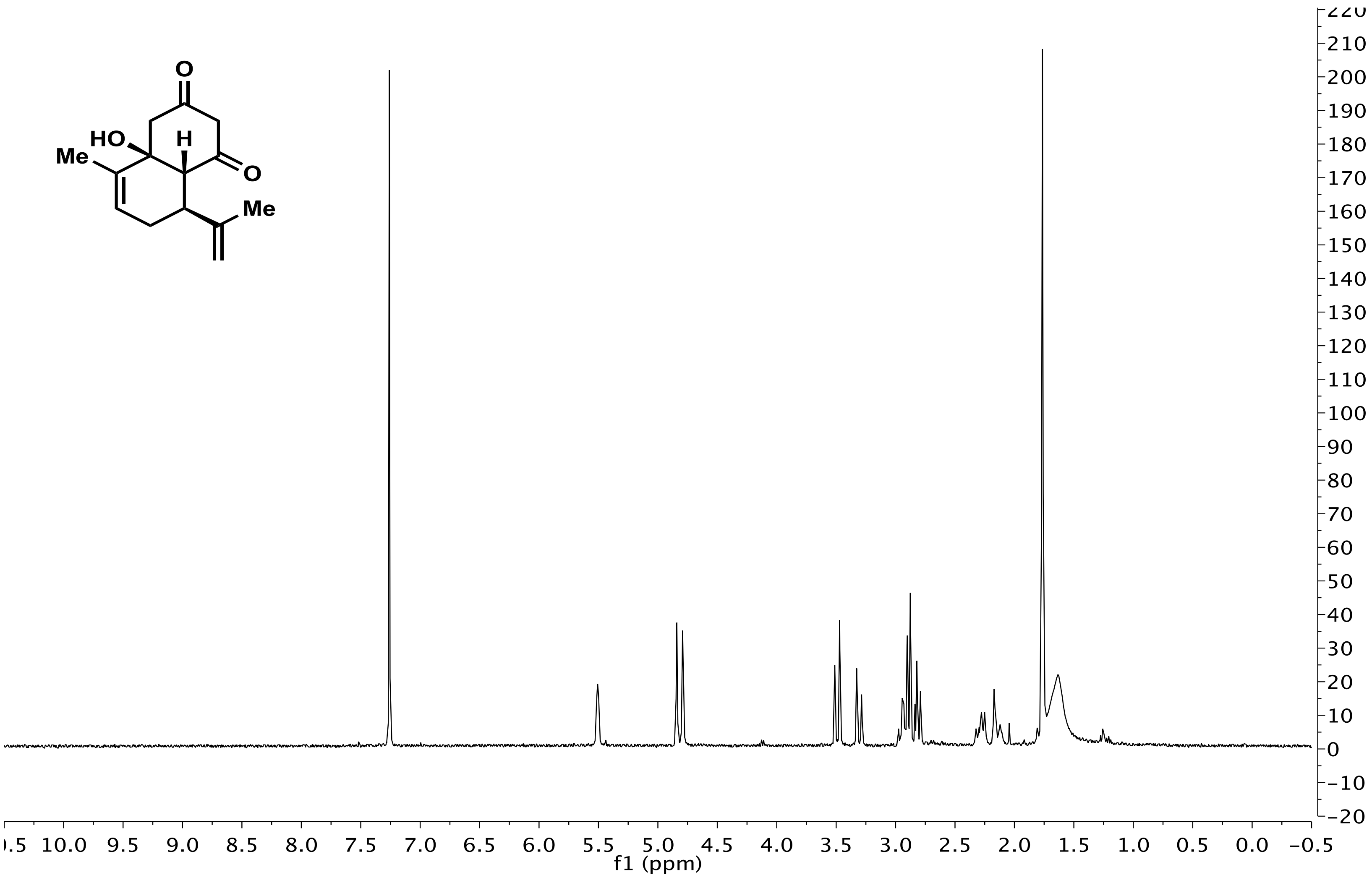

Supplementary Figure 47. ^{1}H NMR of **45**

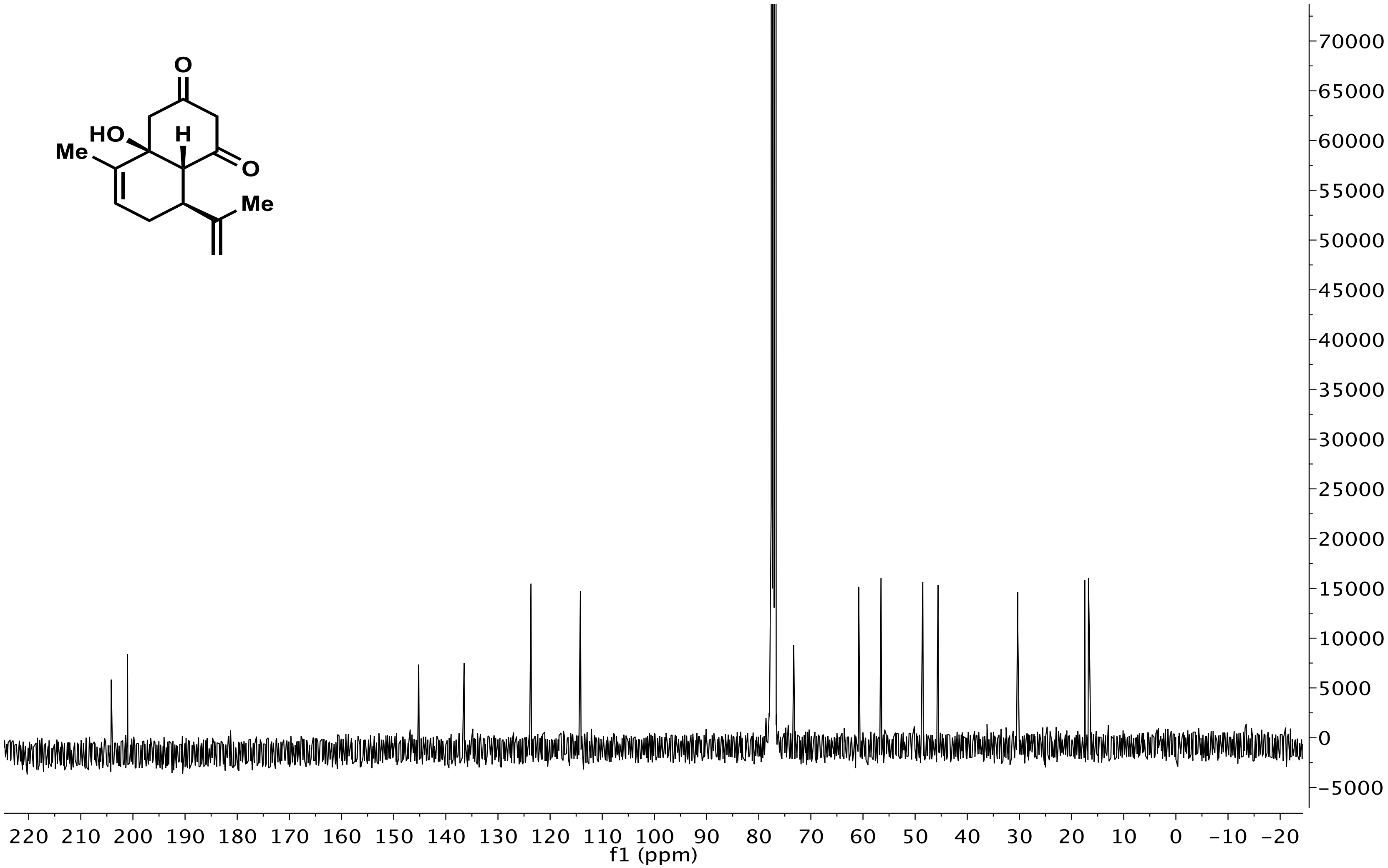

Supplementary Figure 48. ^{13}C NMR of **45**

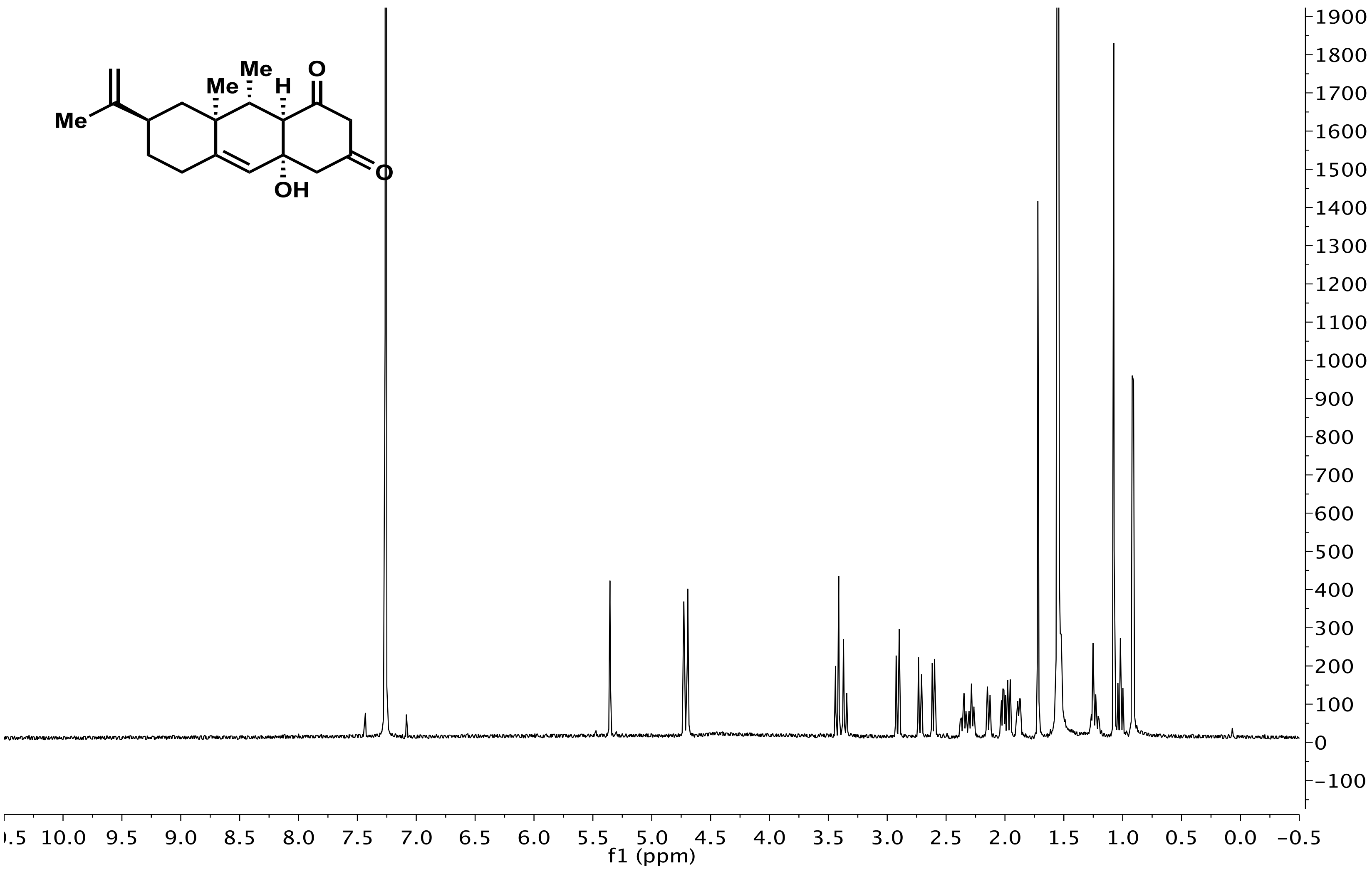

Supplementary Figure 49. ^{1}H NMR of **46**

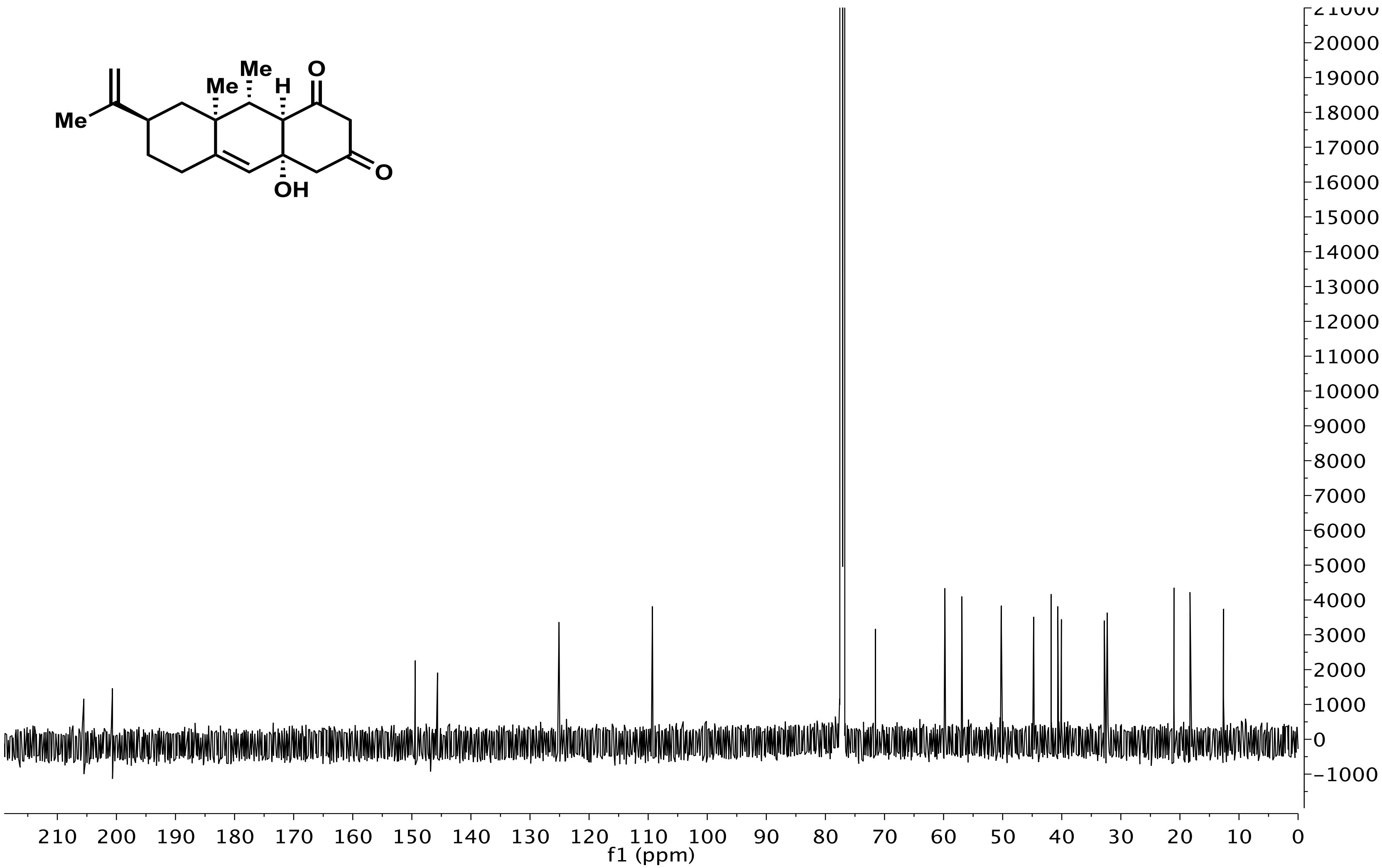

Supplementary Figure 50. ^{13}C NMR of **46**

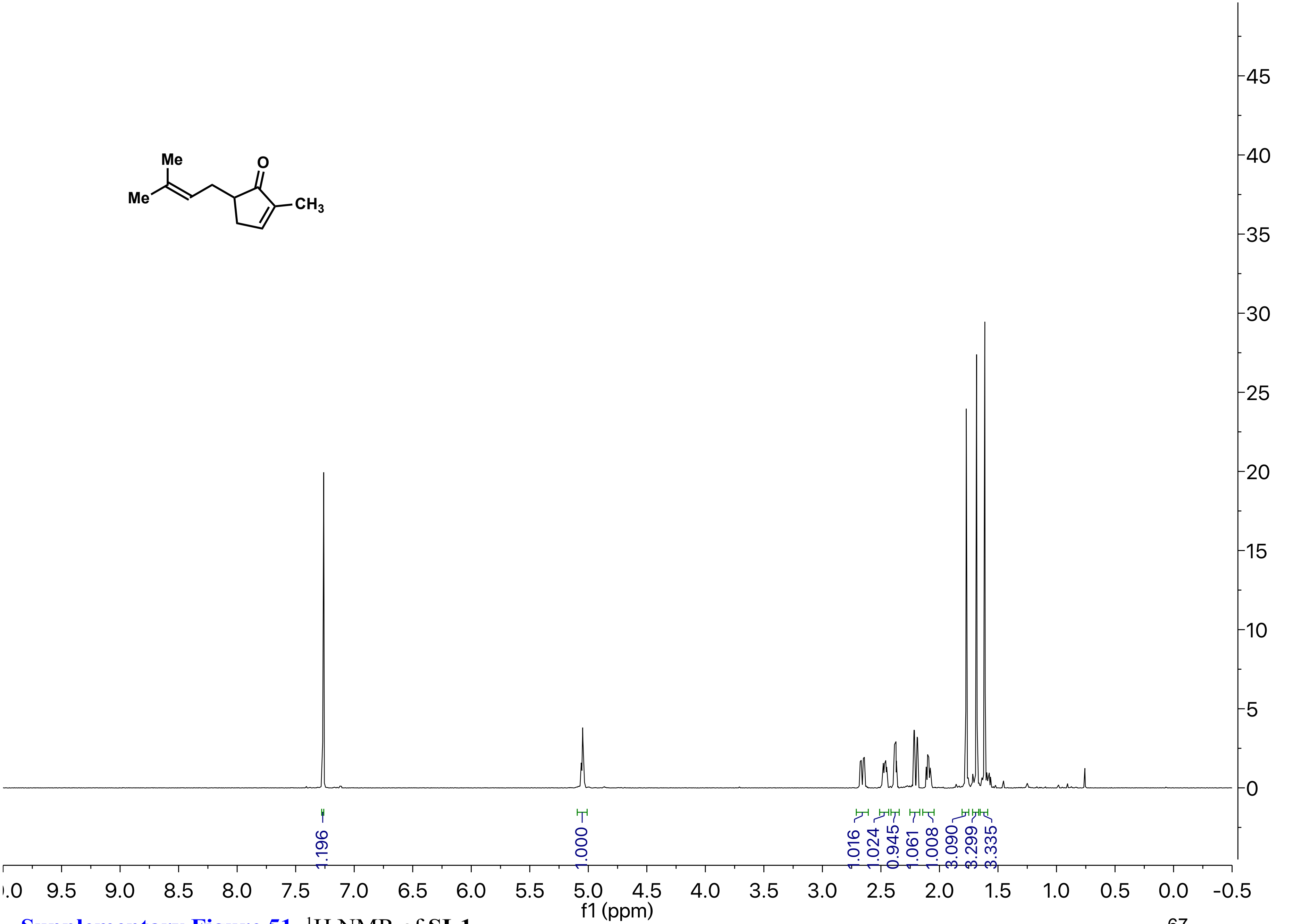

Supplementary Figure 51. ^{1}H NMR of **SI-1**

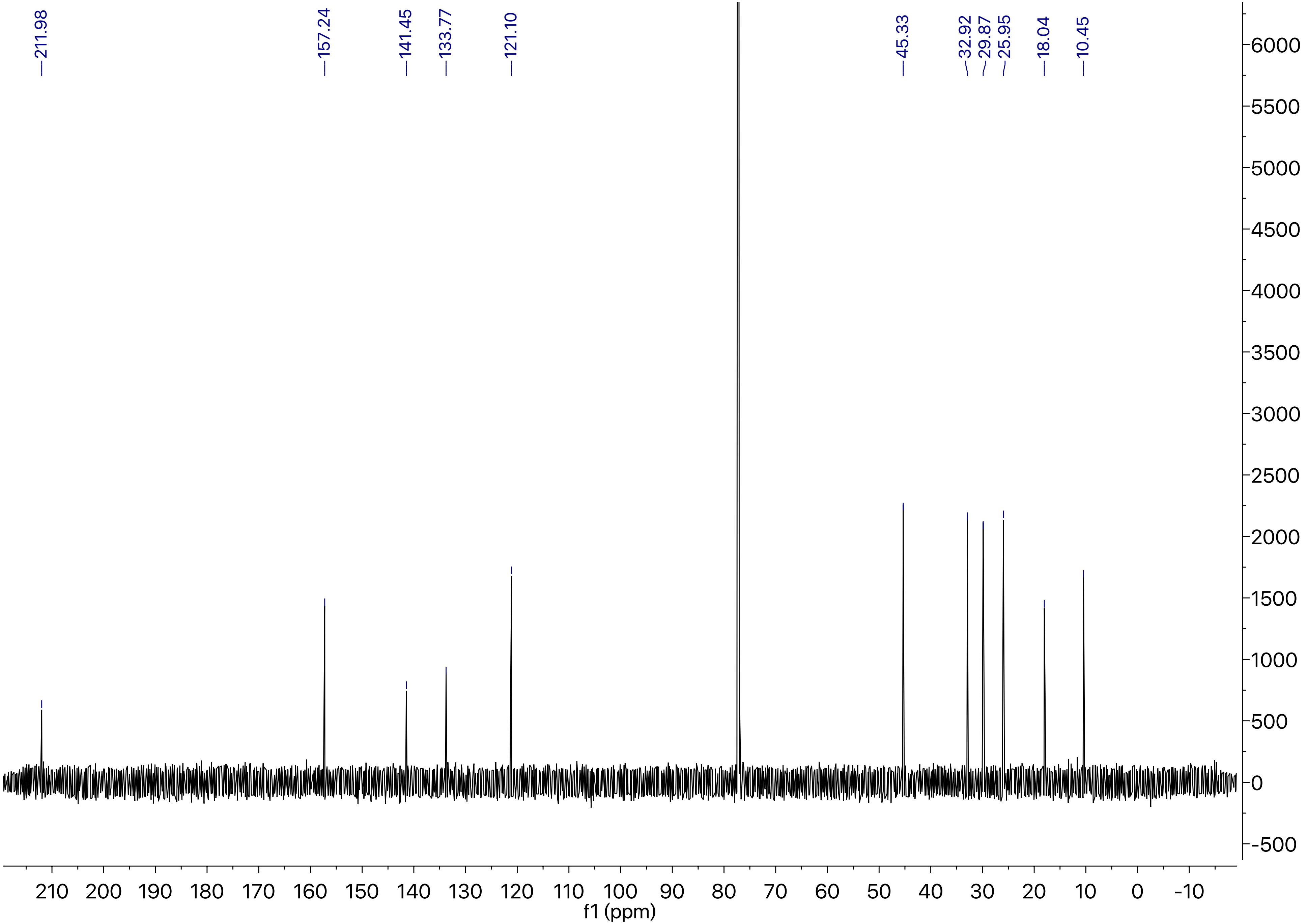

Supplementary Figure 52. ^{13}C NMR of **SI-1**

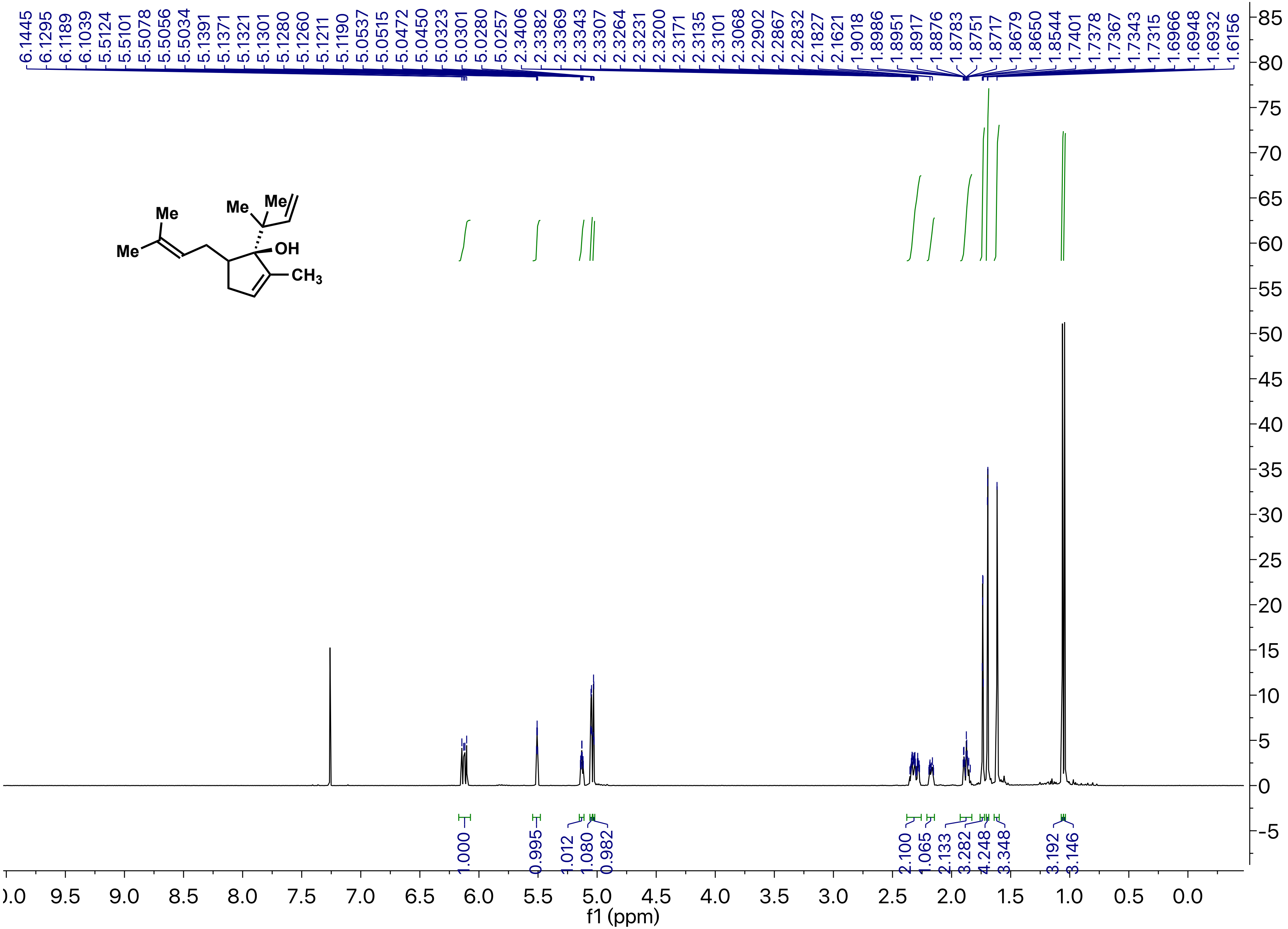

Supplementary Figure 53. ^{1}H NMR of **47**

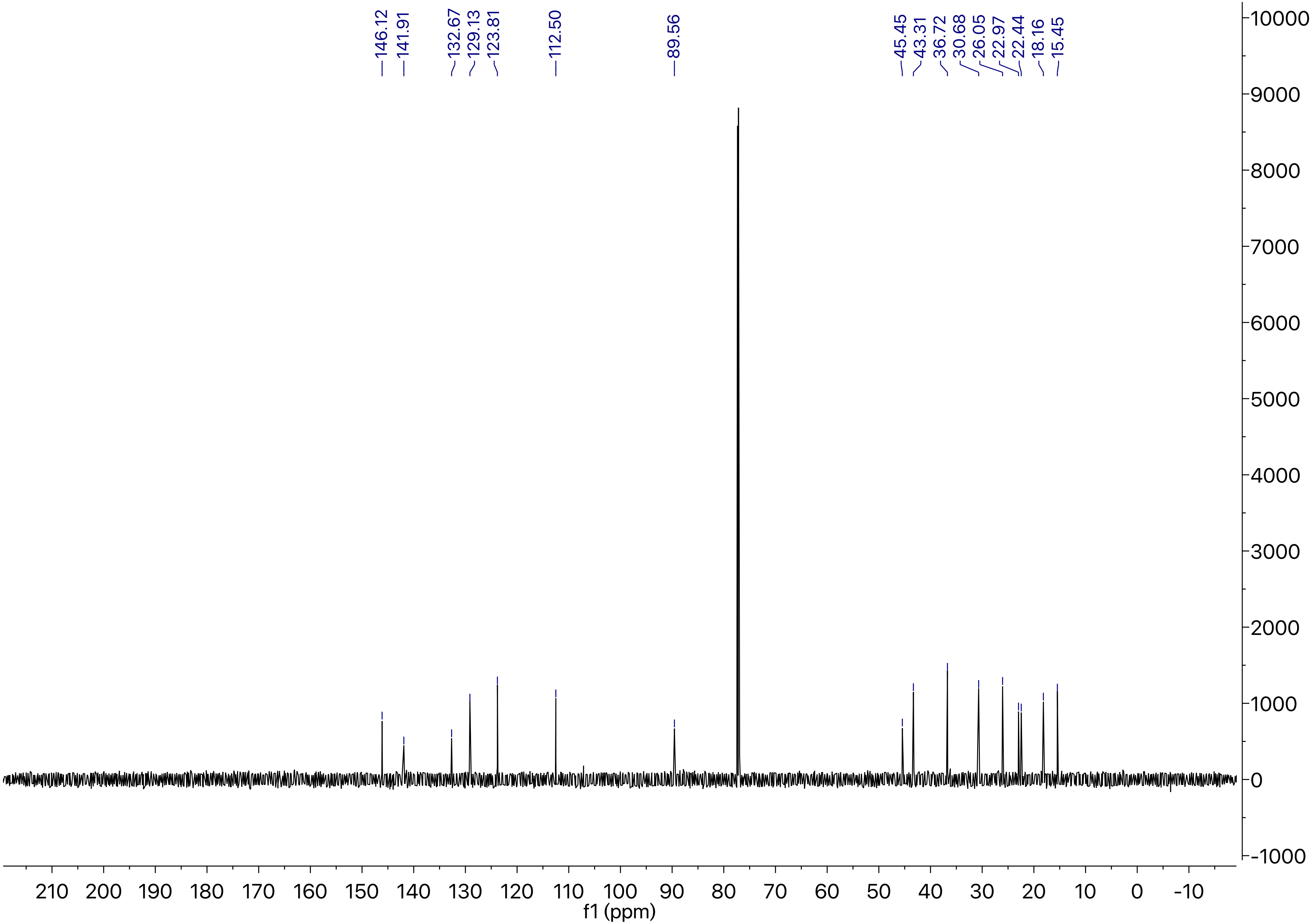

Supplementary Figure 54. ^{13}C NMR of **47**

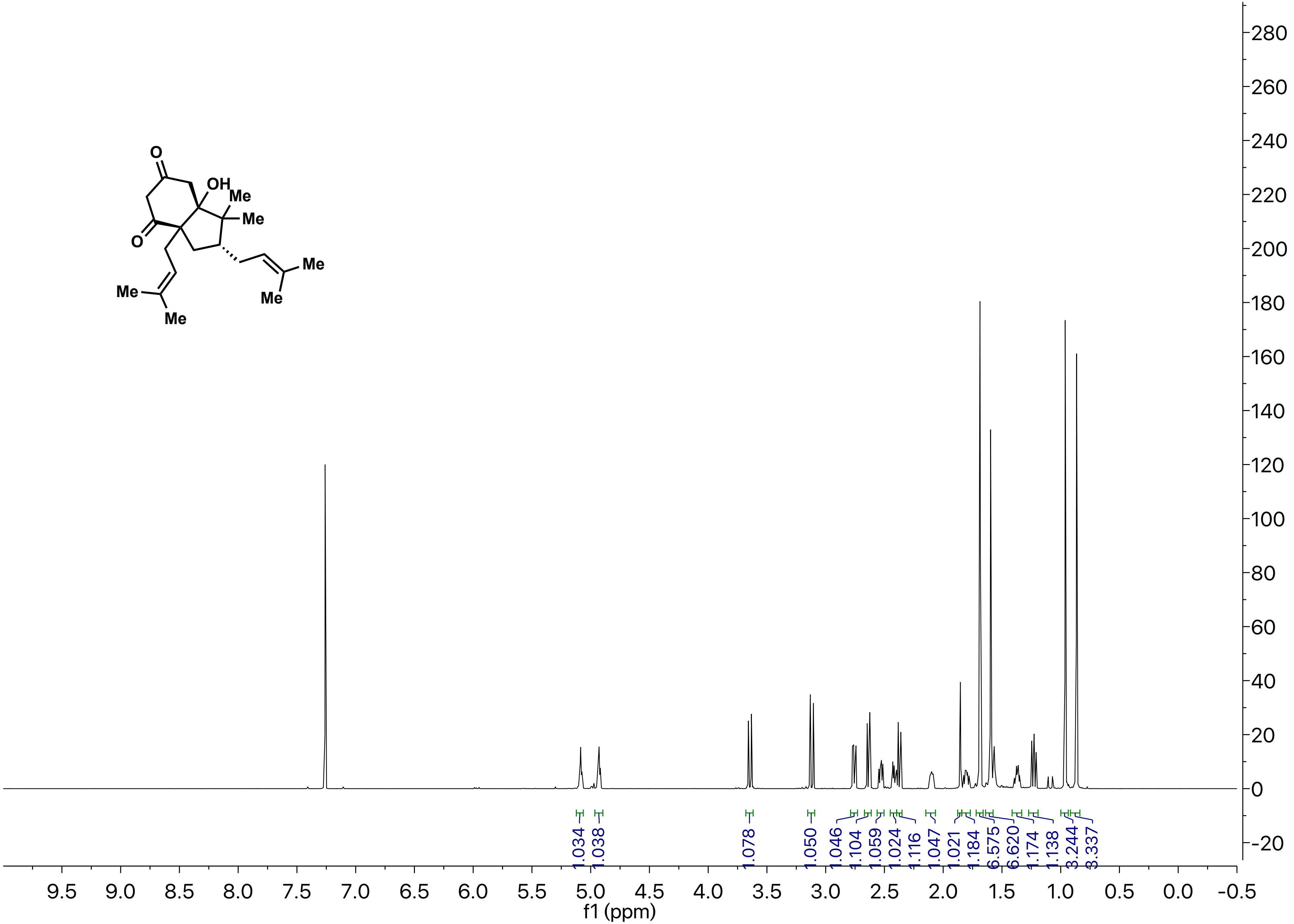

Supplementary Figure 55. ^{1}H NMR of **49**

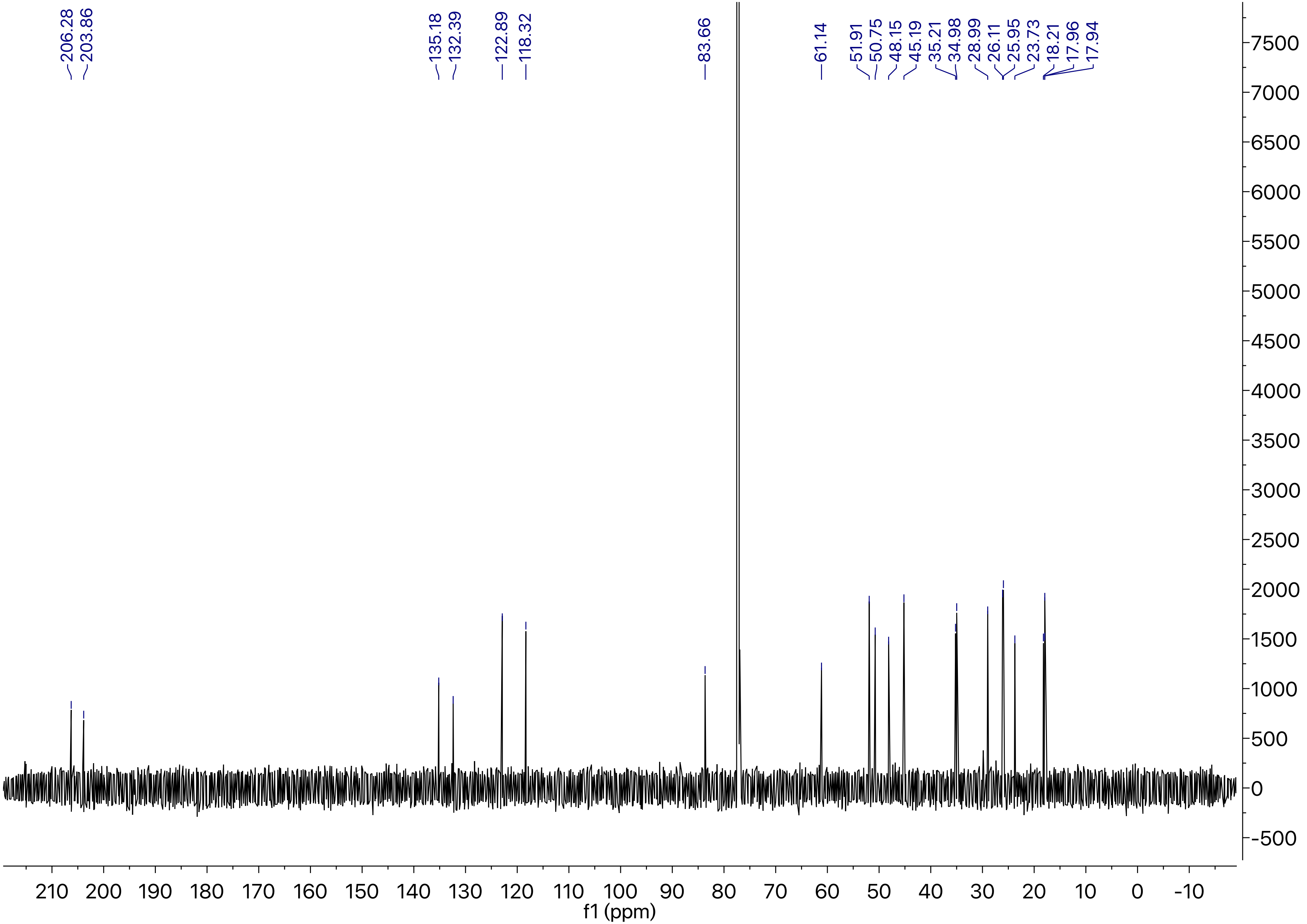

Supplementary Figure 56. ^{13}C NMR of **49**

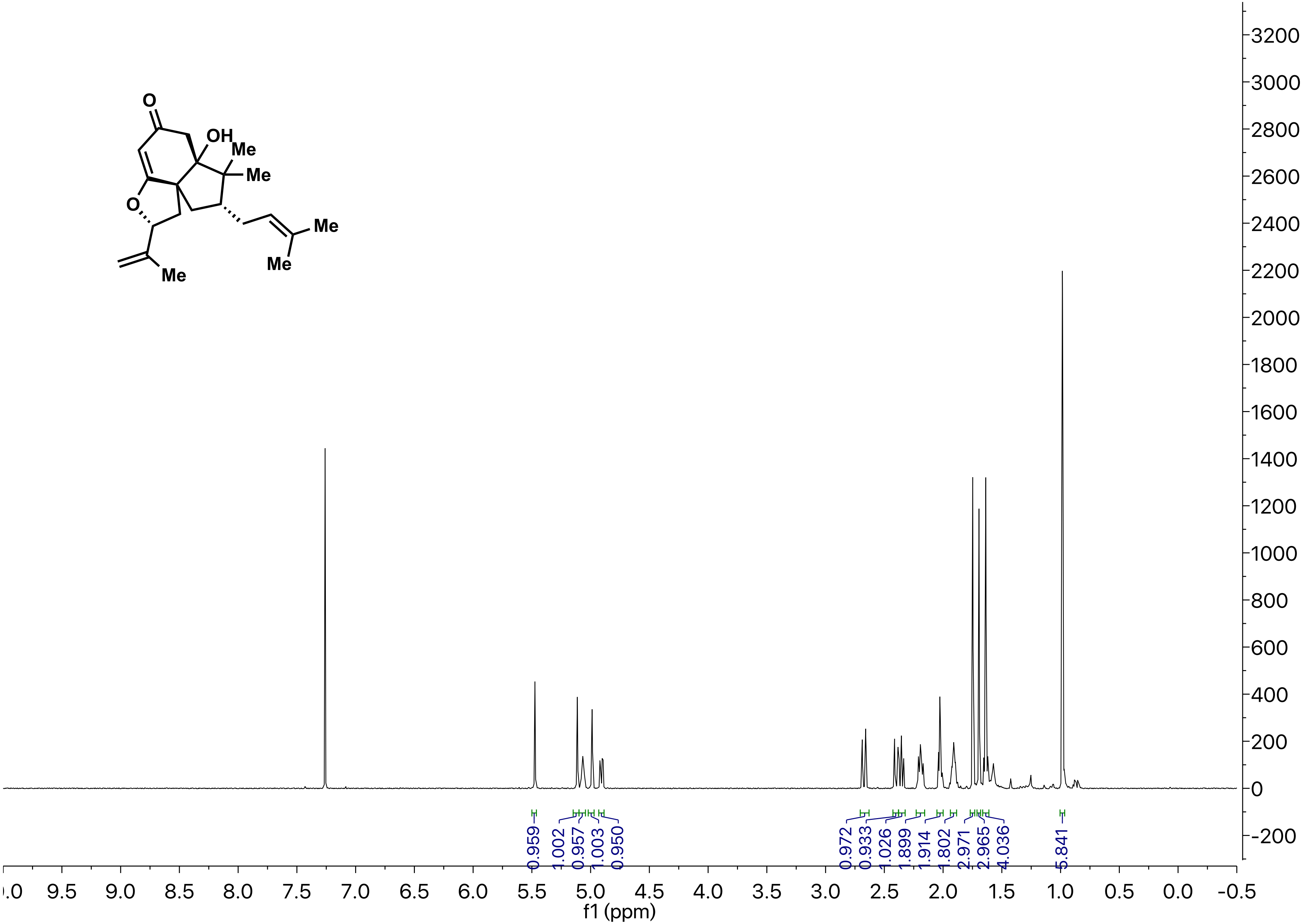

Supplementary Figure 57. ^{1}H NMR of **52**

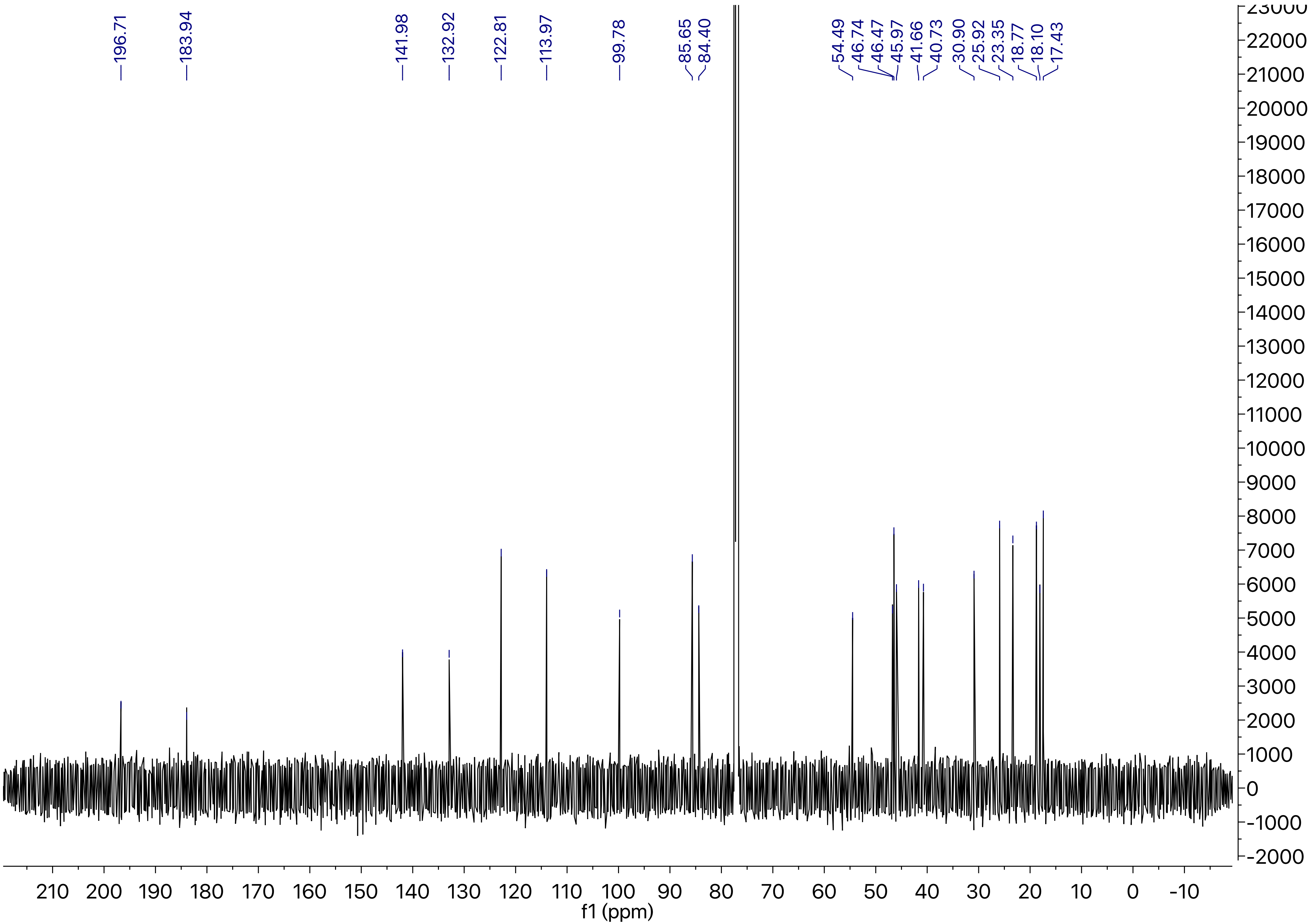

Supplementary Figure 58. ^{13}C NMR of **52**

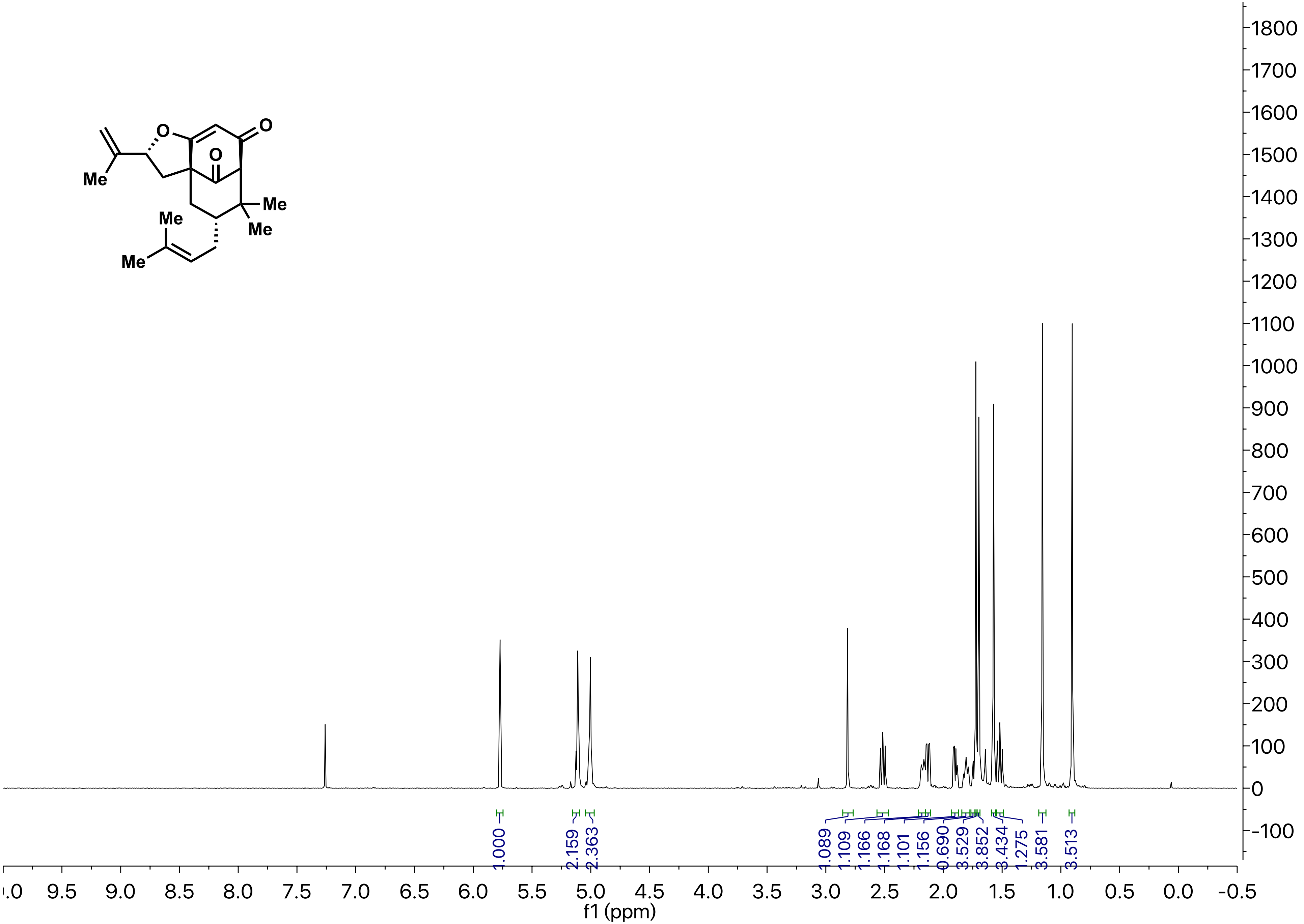

Supplementary Figure 59. ^{1}H NMR of **55**

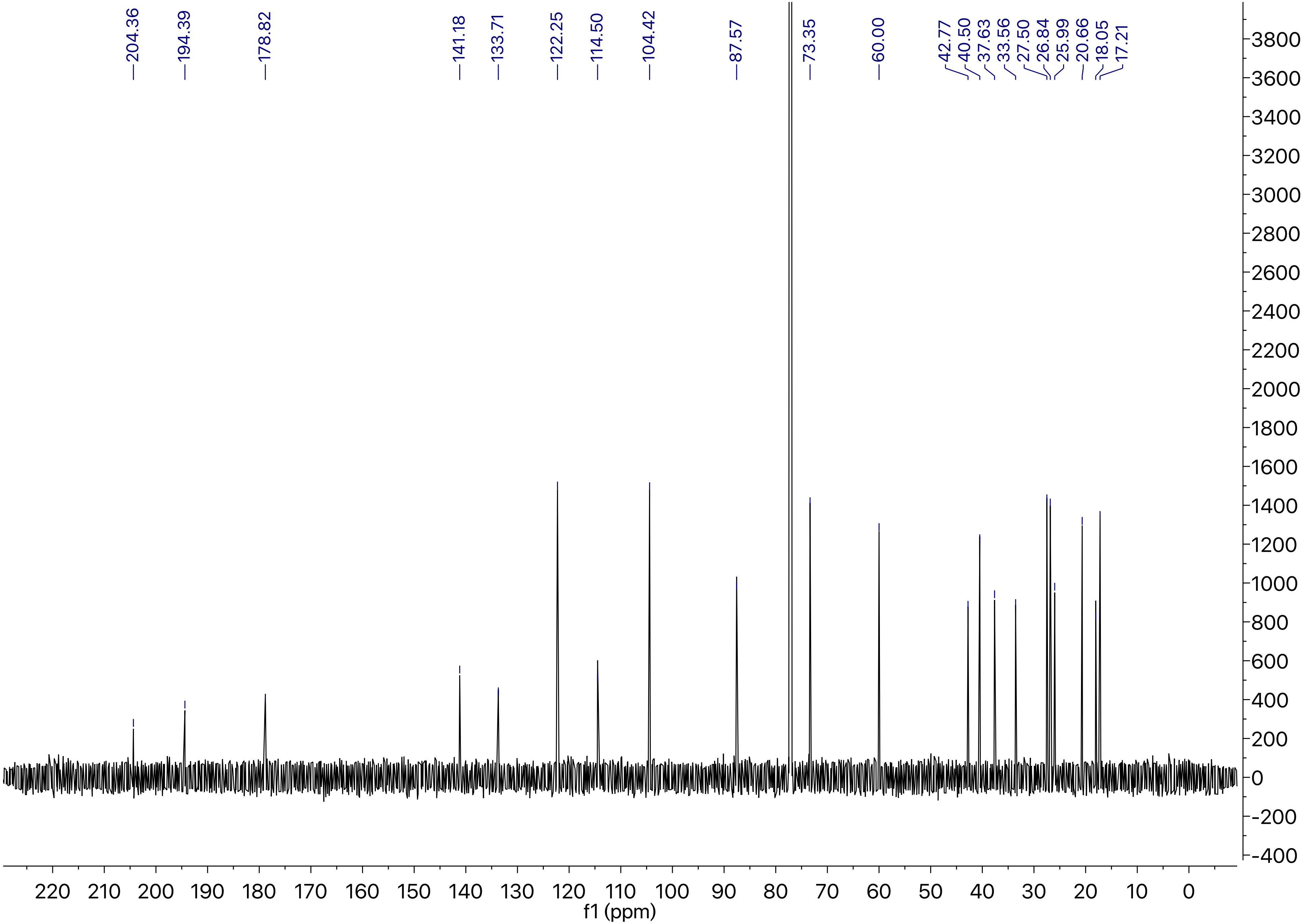

Supplementary Figure 60. ^{13}C NMR of **55**

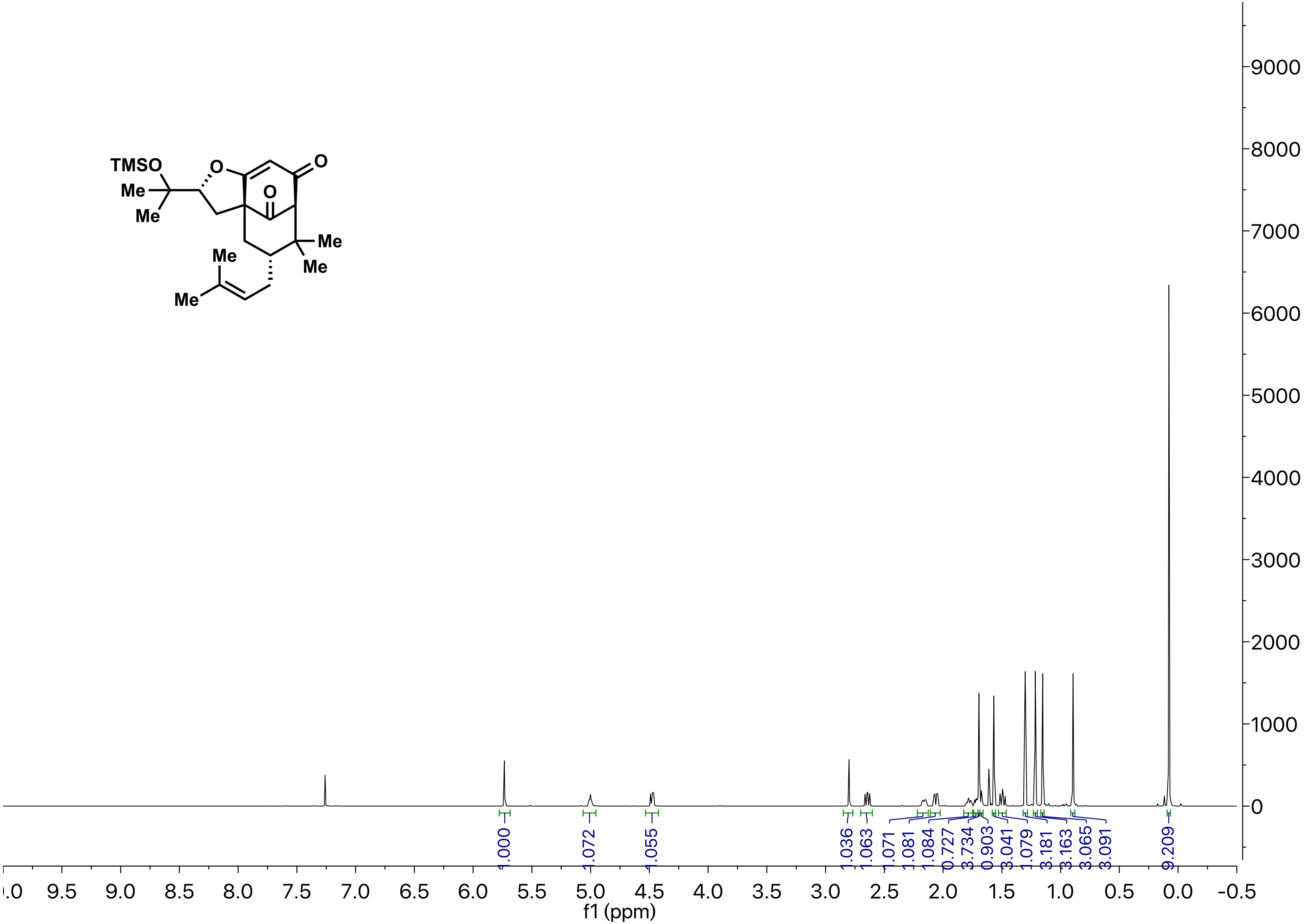

Supplementary Figure 61. ^{1}H NMR of **56**

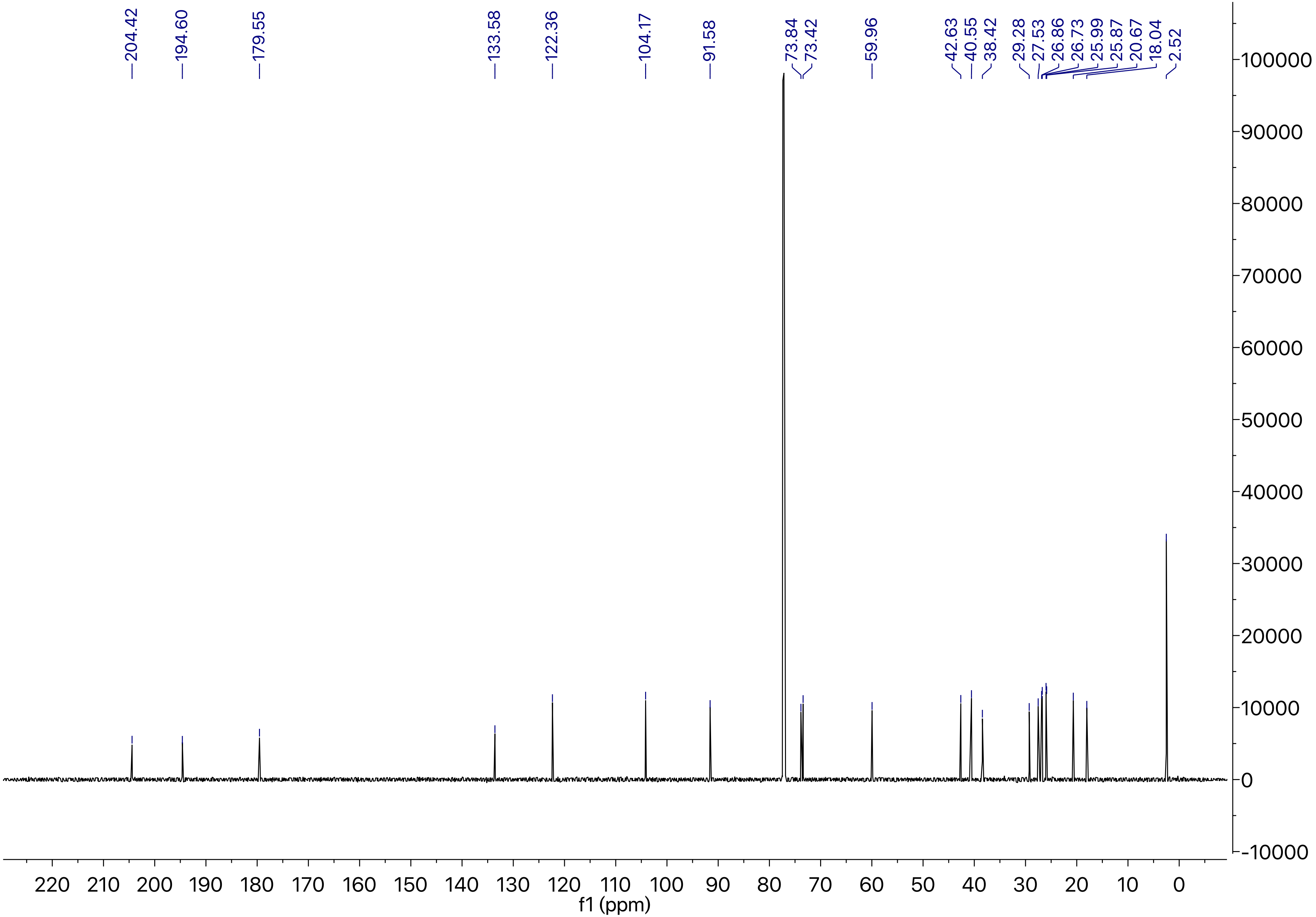

Supplementary Figure 62. ^{13}C NMR of **56**

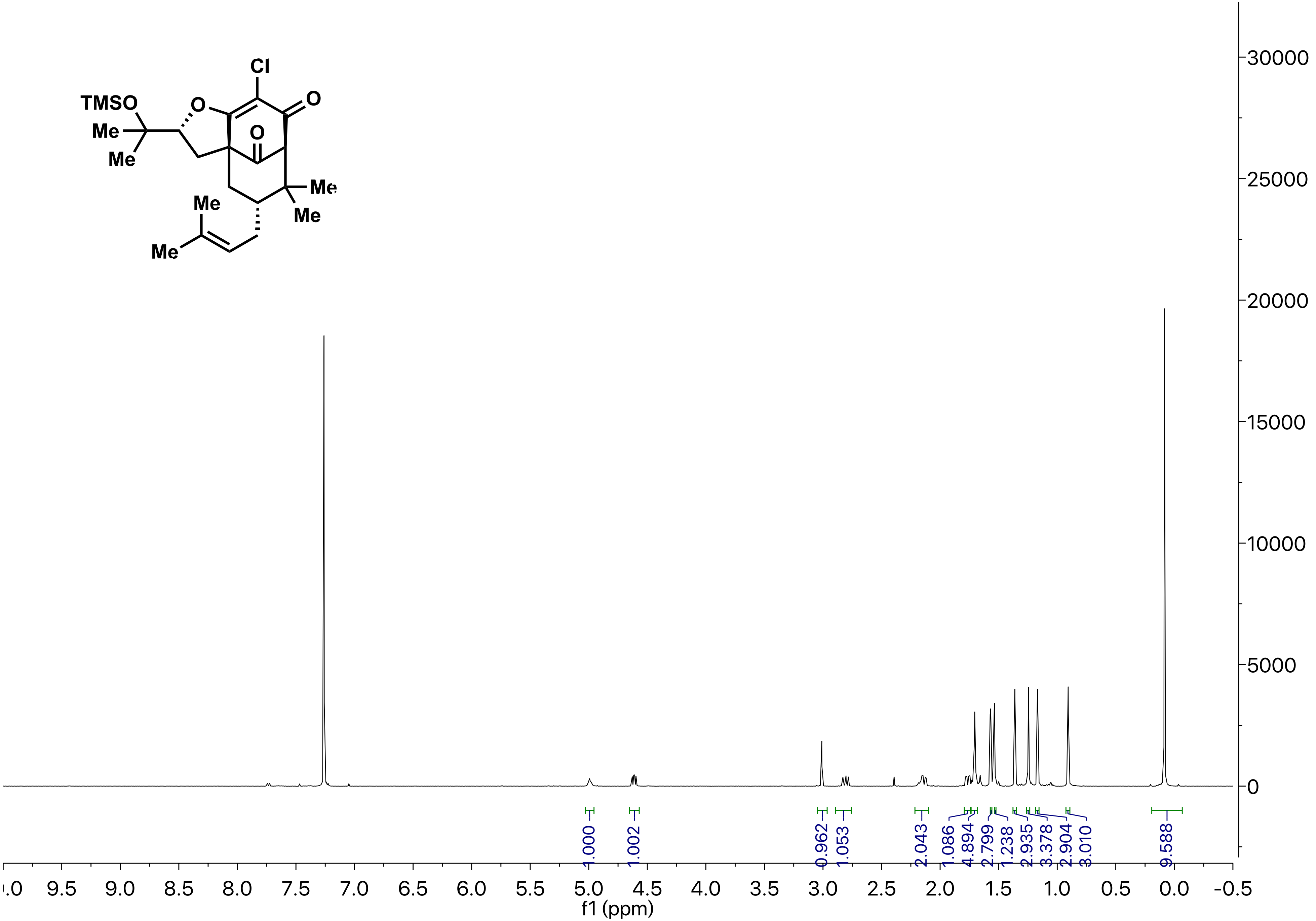

Supplementary Figure 63. ^{1}H NMR of **57**

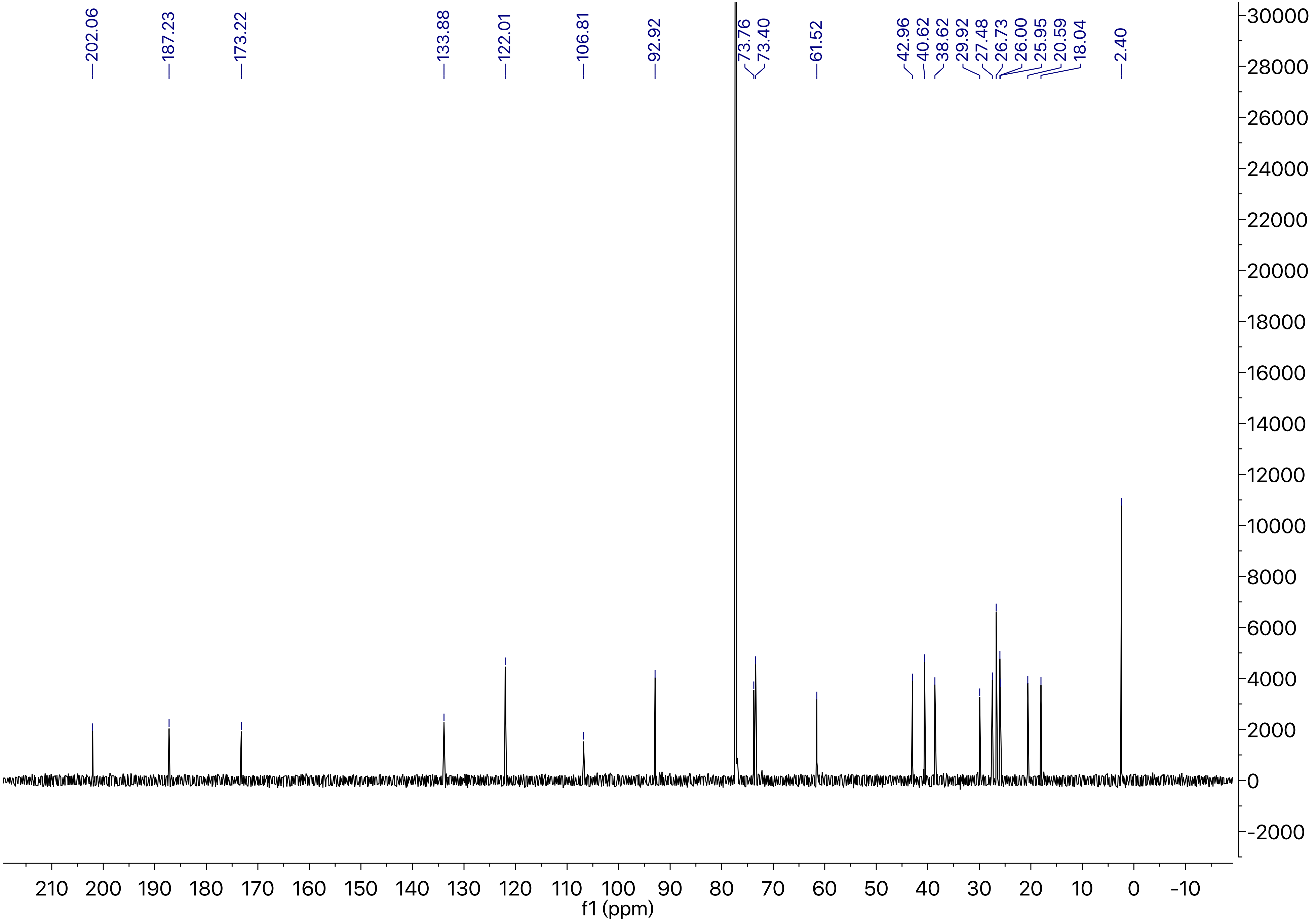

Supplementary Figure 64. ^{13}C NMR of **57**

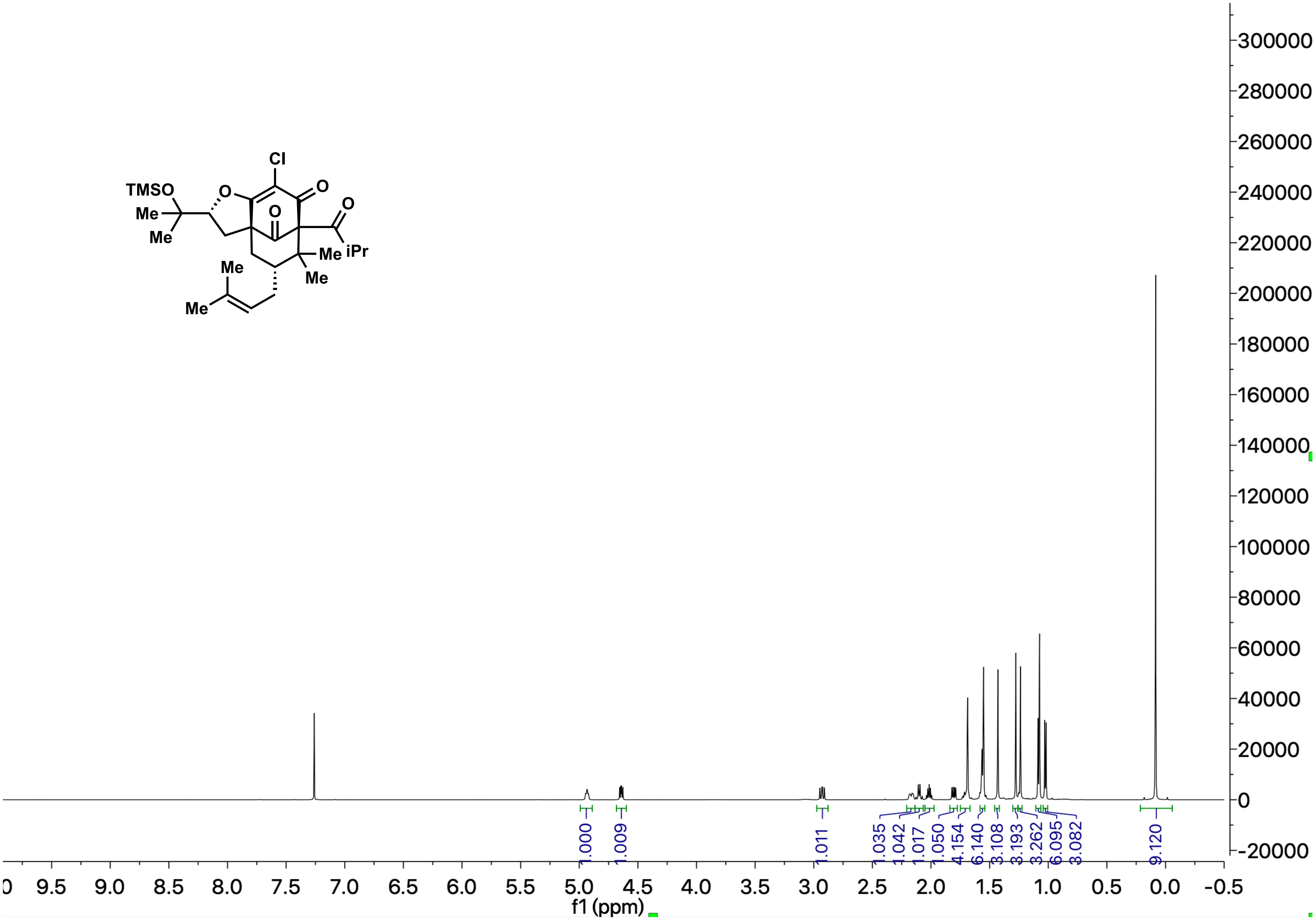

Supplementary Figure 65. ^{1}H NMR of **58**

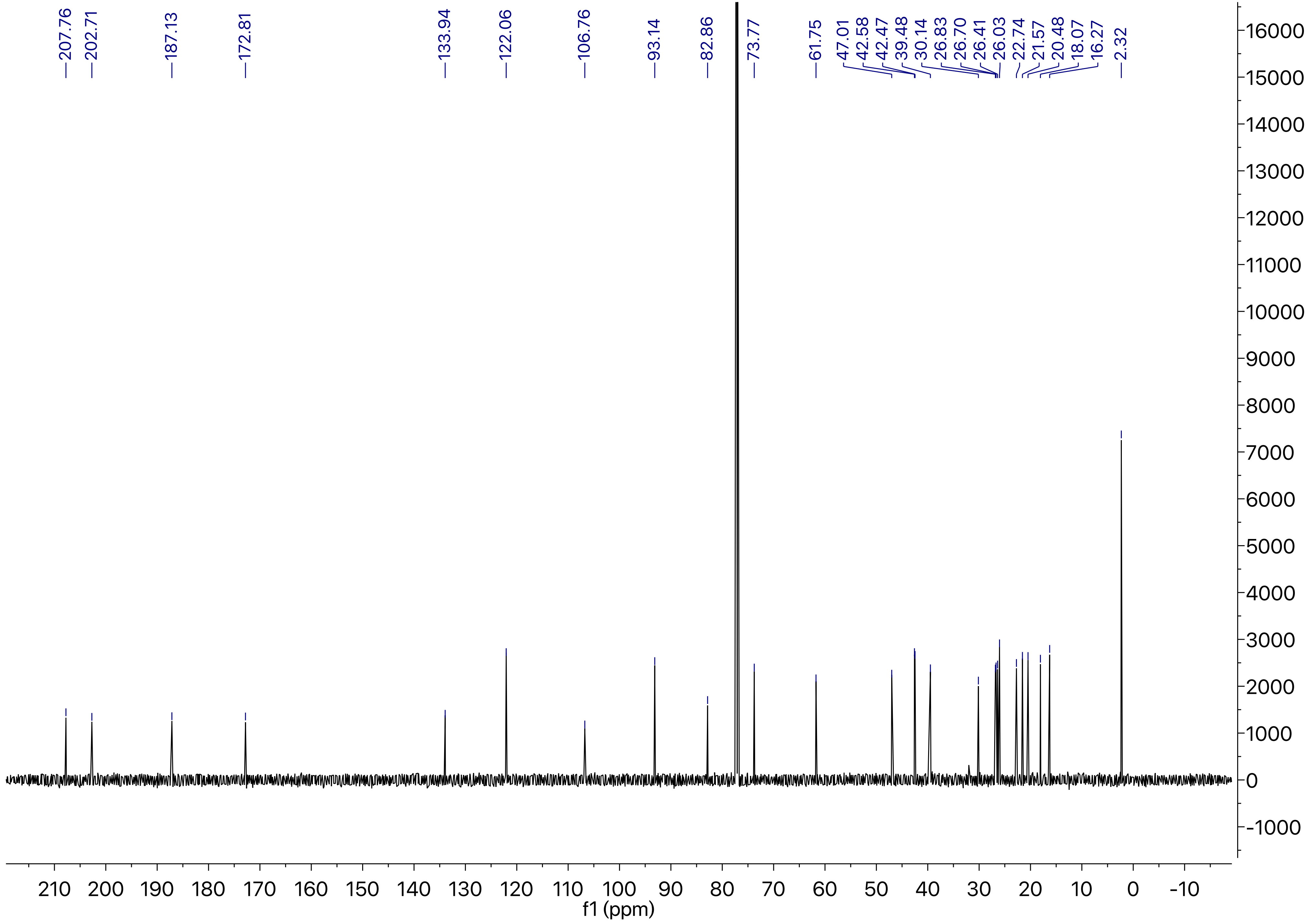

Supplementary Figure 66. ^{13}C NMR of **58**

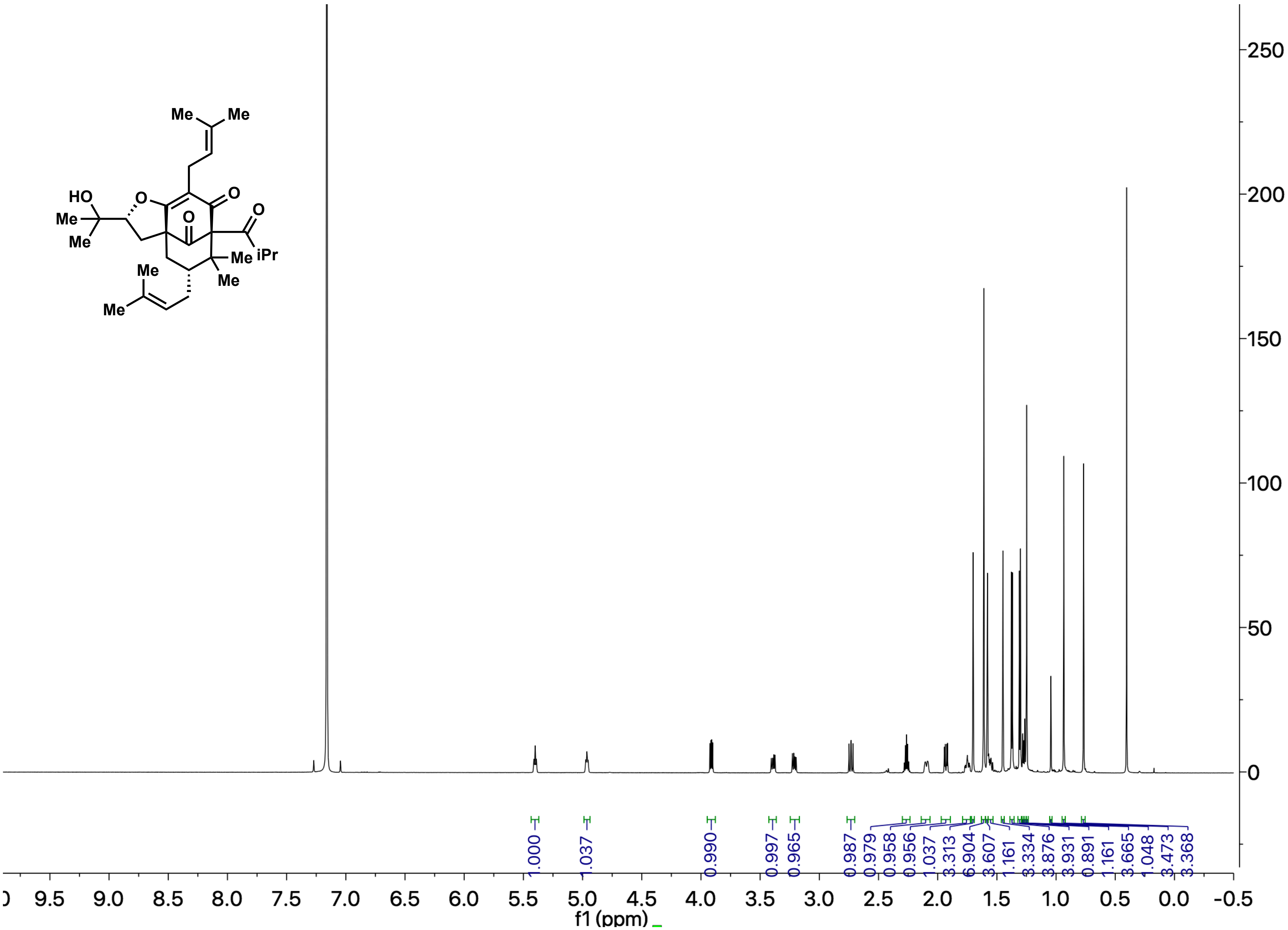

Supplementary Figure 67. ^{1}H NMR of **8**

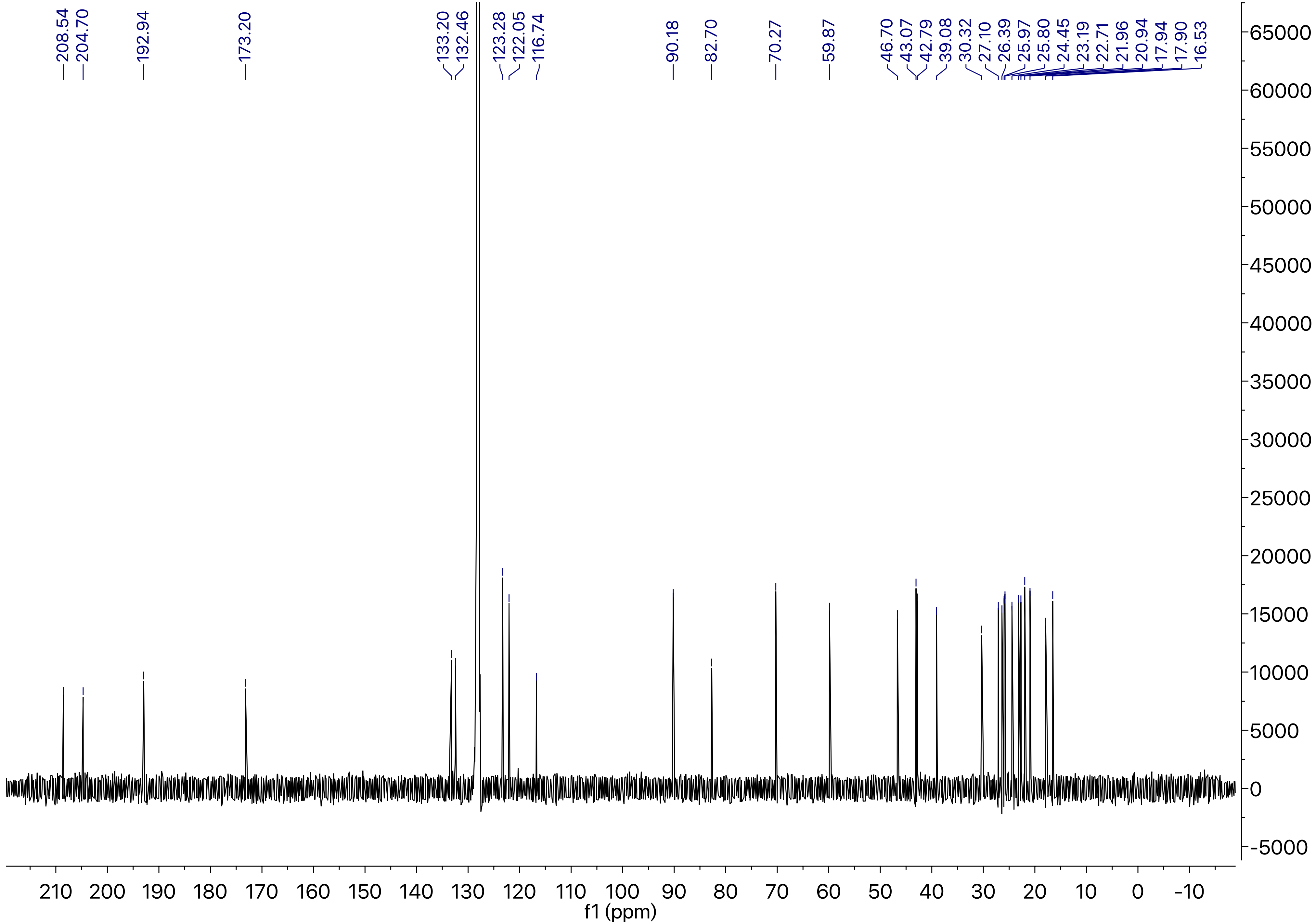

Supplementary Figure 68. ^{13}C NMR of **8**

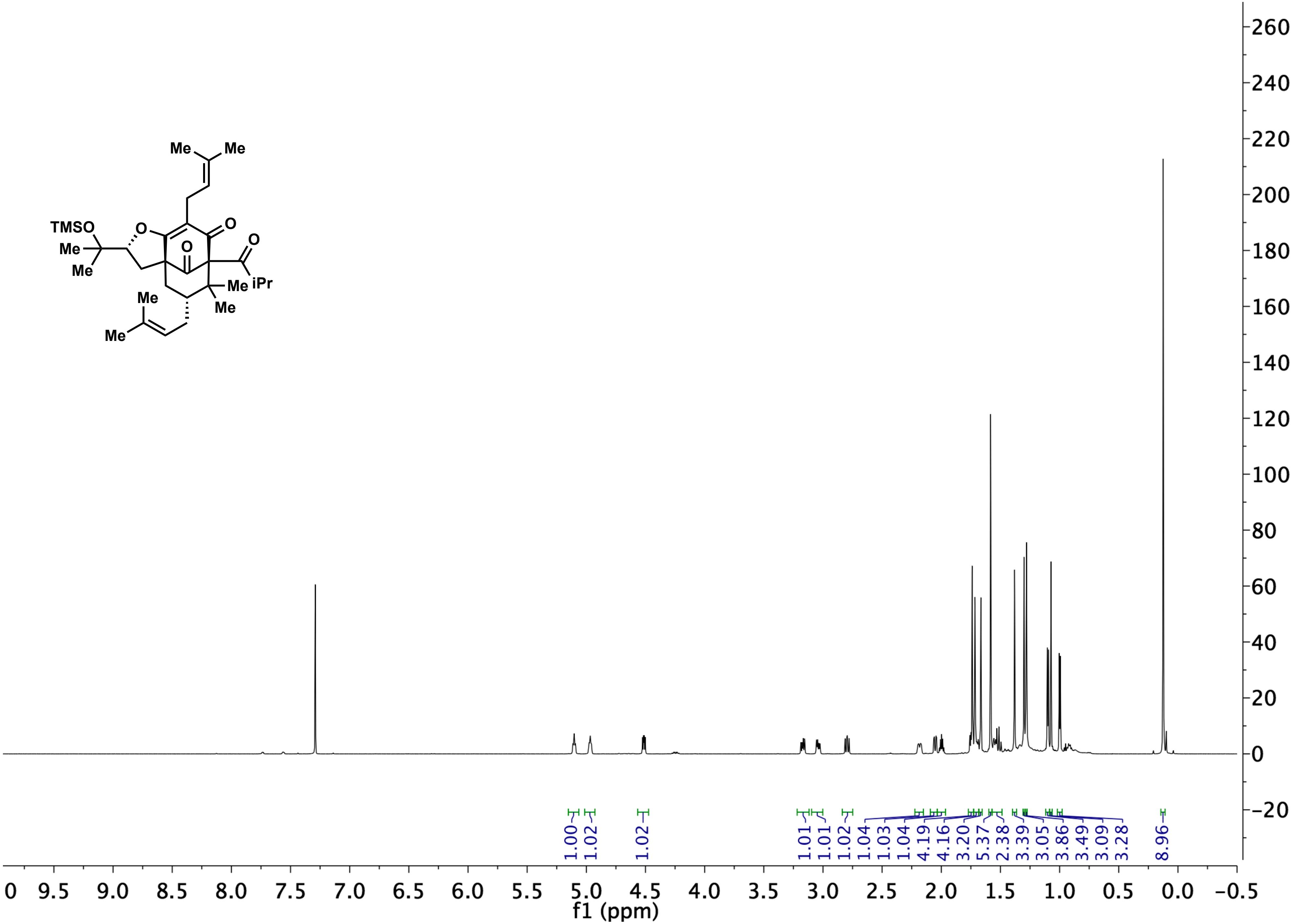

Supplementary Figure 69. ^{1}H NMR of **59**

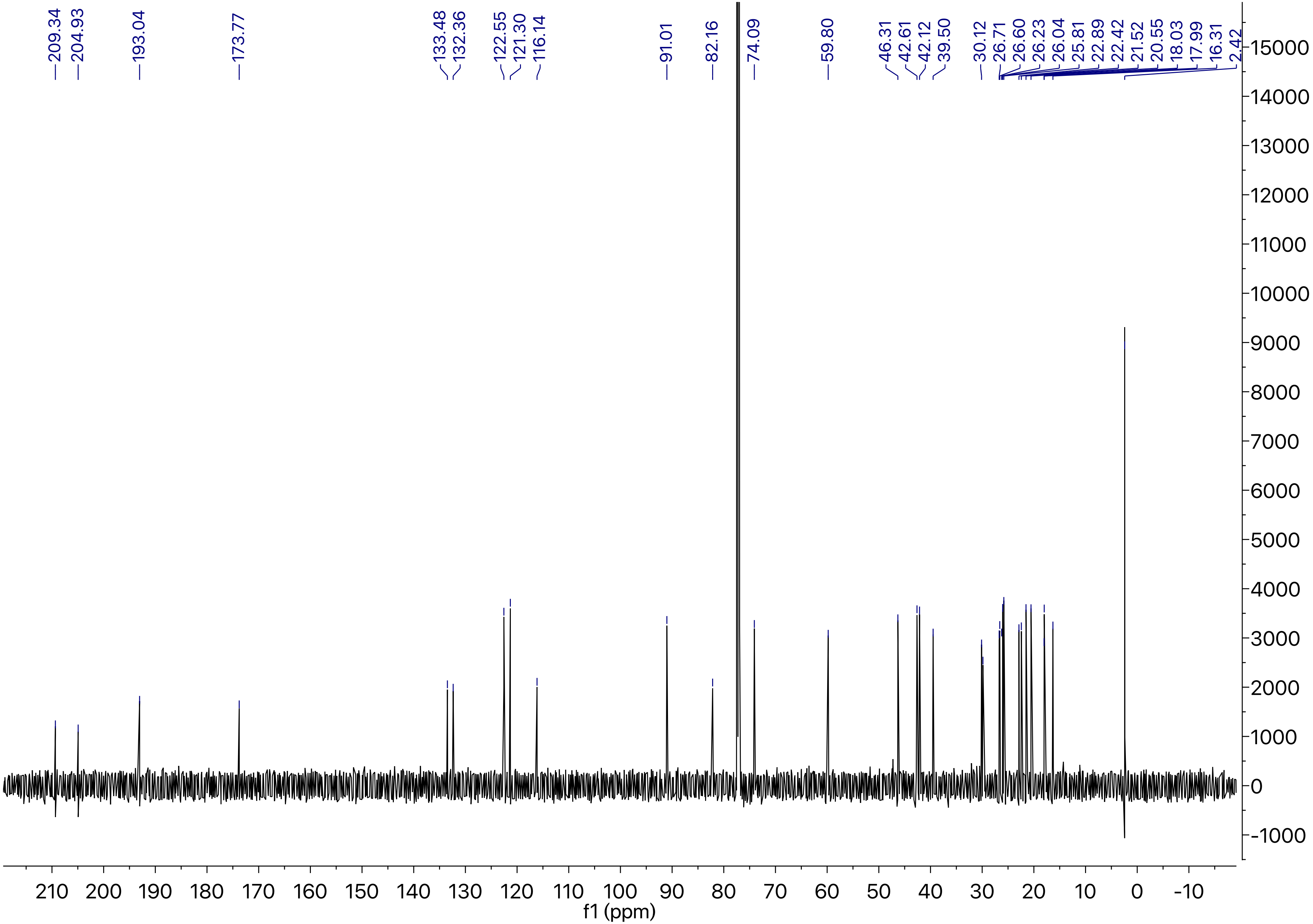

Supplementary Figure 70. ^{13}C NMR of **59**

Supplementary Table 1. ^{13}C NMR comparison of garsubellin A

Garsubellin A Spectral Comparison

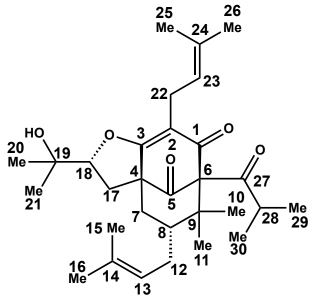

Position	This Work	Danishefsky *et al.*[1]	Nakada *et al.*[2]	Natural[3]
**1**	193.0	193.3	193.0	192.9
**2**	116.7	117.1	116.7	116.7
**3**	173.2	173.6	173.3	173.2
**4**	59.9	60.2	59.9	59.8
**5**	204.7	205.0	204.7	204.7
**6**	82.7	83.0	82.7	82.6
**7**	39.1	39.4	39.1	39.0
**8**	43.1	43.3	43.1	43.0
**9**	46.7	47.0	46.7	46.6
**10**	16.5	16.9	16.5	16.5
**11**	23.2	23.5	23.2	23.1
**12**	27.1	27.4	27.1	27.0
**13**	123.3	123.6	123.3	123.2
**14**	133.2	133.5	133.2	133.2
**15**	17.93	18.2	17.94	17.8
**16**	26.0	26.3	26.0	25.9
**17**	30.3	30.6	30.3	30.3
**18**	90.2	90.5	90.2	90.1
**19**	70.3	70.6	70.3	70.2
**20**	26.4	26.7	26.4	26.3
**21**	24.5	24.8	24.5	24.4
**22**	22.7	23.0	22.7	22.6
**23**	122.1	122.4	122.1	122.0
**24**	132.5	132.8	132.4	132.4
**25**	17.90	18.3	17.90	17.9
**26**	25.8	26.1	25.8	25.7
**27**	208.5	208.9	208.6	208.5
**28**	42.8	43.1	42.7	42.7
**29**	22.0	22.3	21.9	21.9
**30**	20.9	21.3	20.9	20.9

Supplementary Table 2. ^{1}H NMR comparison of garsubellin A

position	This work	Danishefsky group [1]	Shibasaki Group[4]	Nakada group[2]	natural garsubellin a[3]
7	1.26 (m, 1H)	1.32- 1.30 (m, 2H)			1.30 (dd, *J* = 11.3, 13.6 Hz, 1H)
	1.29 – 1.27 (m, 1H)	1.32- 1.30 (m, 2H)			1.32 (dd, *J* = 5.9, 12.9 Hz, 1H)
8	1.75 (dddd, J = 12.5, 10.5, 4.6, 2.8 Hz, 1H)	1.74 (m, 1H)	1.74 (m, 1H)	1.74 (m, 1H)	1.74 (dddd, *J* = 3.6, 4.5, 7.1, 11.3 Hz, 1H)
10	1.25 (s, 3H)	1.24 (s, 3H)	1.24 (s, 3H)	1.23 (s, 3H)	1.24 (s, 3H)
11	1.61 (s, 6H)	1.61 (s, 6H)	1.60 (s, 3H)	1.60 (s, 3H)	1.60 (s, 3H)
12	1.57 – 1.53 (m, 1H)	1.58 (m, 1H)	1.58 (m, 1H)	1.58 (m, 1H)	1.58 (m, 1H)
	2.15 – 2.02 (m, 1H)	2.09 (m, 1H)	2.08 (m, 1H)	2.09 (m, 1H)	2.09 (ddd, *J* = 3.6, 7.1, 13.4 Hz, 1H)
13	5.07 – 4.89 (m, 1H)	4.97 (dd, *J* = 6.9, 7.0 Hz, 1H)	4.97 (dd, *J* = 7.1, 7.1 Hz, 1H)	4.96 (brd, 1H)	4.96 (dd, *J* = 7.1, 7.1 Hz, 1H)
15	1.45 (s, 3H)	1.45 (s, 3H)	1.44 (s, 3H)	1.44 (s, 3H)	1.45 (s, 3H)
16	1.58 (s, 3H)	1.58 (s, 3H)	1.57 (s, 3H)	1.57 (s, 3H)	1.58 (s, 3H)
17	1.93 (dd, J = 13.6, 4.5 Hz, 1H)	1.93 (dd, *J* = 4.5, 13.6 Hz, 1H)	1.92 (dd, *J* = 4.6, 13.7 Hz, 1H)	1.93 (dd, *J* = 4.5, 14.0 Hz, 1H)	1.93 (dd, *J* = 4.5, 13.6 Hz, 1H)
	2.73 (dd, J = 13.0, 10.7 Hz, 1H)	2.73 (dd, *J* = 10.8, 13.1 Hz, 1H)	2.72 (dd, *J* = 10.8, 13.1 Hz, 1H)	2.73 (dd, *J* = 10.5, 13.0 Hz, 1H)	2.73 (dd, *J* = 10.7, 12.9 Hz, 1H)
18	3.91 (dd, J = 10.7, 5.8 Hz, 1H)	3.92 (dd, *J* = 5.8, 10.6 Hz, 1H)	3.91 (dd, *J* = 6.3, 10.8 Hz, 1H)	3.93 (dd, *J* = 5.5, 11.0 Hz, 1H)	3.92 (dd, *J* = 5.9, 10.7 Hz, 1H)
20	0.93 (s, 3H)	0.94 (s, 3H)	0.93 (s, 3H)	0.95 (s, 3H)	0.94 (s, 3H)
21	0.77 (s, 3H)	0.77 (s, 3H)	0.76 (s, 3H)	0.79 (s, 3H)	0.77 (s, 3H)
22	3.21 (dd, J = 14.2, 7.6 Hz, 1H)	3.21 (dd, *J* = 7.6, 14.3 Hz, 1H)	3.20 (dd, *J* = 7.4, 14.3 Hz, 1H)	3.19 (dd, *J* = 8.0, 14.0 Hz, 1H)	3.21 (dd, *J* = 7.3, 14.2 Hz, 1H)
	3.39 (dd, J = 14.2, 7.2 Hz, 1H)	3.39 (dd, *J* = 7.1, 14.1 Hz, 1H)	3.38 (dd, *J* = 7.4, 14.3 Hz, 1H)	3.36 (dd, *J* = 7.5, 14.0 Hz, 1H)	3.39 (dd, *J* = 7.1, 14.2 Hz, 1H)
23	5.40 (ddt, J = 7.4, 5.9, 1.4 Hz, 1H)	5.40 (dd, *J* = 7.3, 7.3 Hz, 1H)	5.39 (dd, *J* = 7.4, 7.4 Hz, 1H)	5.38 (m, 1H)	5.40 (dd, *J* = 7.1, 7.3 Hz, 1H)
25	1.70 (d, J = 1.3 Hz, 3H)	1.70 (s, 3H)	1.69 (s, 3H)	1.69 (s, 3H)	1.70 (s, 3H)
26	1.61 (s, 6H)	1.61 (s, 6H)	1.60 (s, 3H)	1.58 (s, 3H)	1.61 (s, 3H)
28	2.26 (hept, J = 6.5 Hz, 1H)	2.26 (qq, *J* = 6.6, 6.6 Hz, 1H)	2.25 (qq, *J* = 6.8, 6.8 Hz, 1H)	2.24 (qq, *J* = 6.5, 6.5 Hz, 1H)	2.26 (dq, *J* = 6.6 Hz, 1H)
29	1.31 (d, J = 6.5 Hz, 3H)	1.30 (d, *J* = 6.5 Hz, 3H)	1.30 (d, *J* = 6.8 Hz, 3H)	1.28 (d, *J* = 6.5 Hz, 3H)	1.30 (d, *J* = 6.6 Hz, 3H)
30	1.37 (d, J = 6.5 Hz, 3H)	1.37 (d, *J* = 6.5 Hz, 3H)	1.36 (d, *J* = 6.8 Hz, 3H)	1.34 (d, *J* = 6.8 Hz, 3H)	1.37 (d, *J* = 6.6 Hz, 3H)

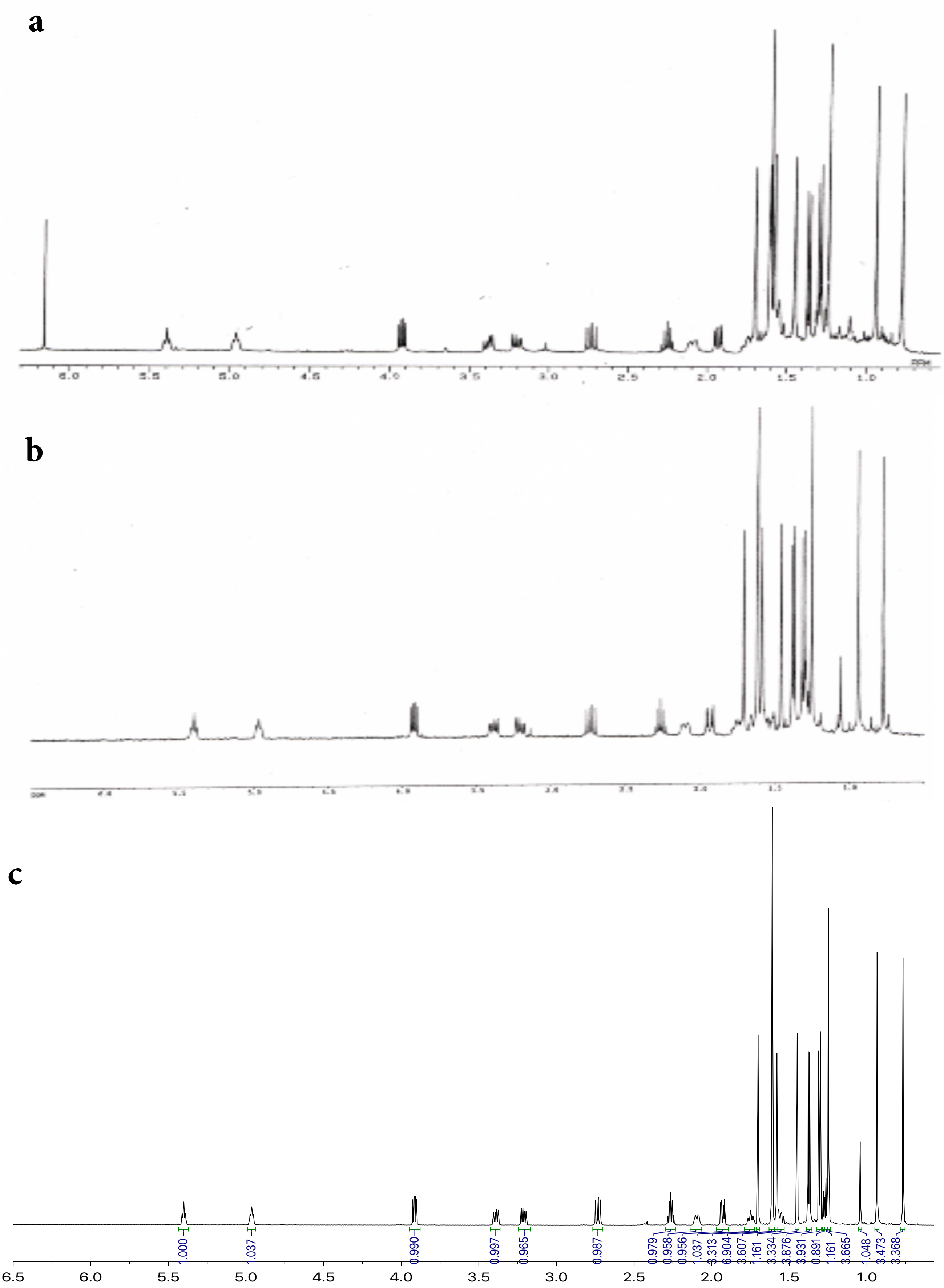

Supplementary Figure 71. Garsubellin A ^{1}H NMR comparison. **a** Natural[3] garsubellin A **b** Danishefsky's[1] synthetic garsubellin A **c** Synthetic garsubellin A prepared in this work.

Supplementary References

1. Siegel, D. R. & Danishefsky, S. J. Total Synthesis of Garsubellin A. *J. Am. Chem. Soc.* **128**, 1048–1049 (2016).
2. Uwamori, M. & Nakada, M. Stereoselective total synthesis of garsubellin A. *J. Antibiot.* **66**, 141–145 (2013).
3. Fukuyama, Y., Kuwayama, A. & Minami, H. Garsubellin A, A Novel Polyprenylated Phloroglucin Derivative, Increasing Choline Acetyltransferase (ChAT) Activity In Postnatal Rat Septal Neuron Cultures. *Chem. Pharm. Bull*. **45**, 947–949 (1997).
4. Kuramochi, A., Usuda, H., Yamatsuga, K., Kanai, M. & Shibasaki, M. Total Synthesis of (±)-Garsubellin A. *J. Am. Chem. Soc*. **127**, 14200–14201 (2005).
